# biotoolsSchema: a formalized schema for bioinformatics software description

**DOI:** 10.1093/gigascience/giaa157

**Published:** 2021-01-27

**Authors:** Jon Ison, Hans Ienasescu, Emil Rydza, Piotr Chmura, Kristoffer Rapacki, Alban Gaignard, Veit Schwämmle, Jacques van Helden, Matúš Kalaš, Hervé Ménager

**Affiliations:** CNRS, UMS 3601, Institut Français de Bioinformatique, IFB-core, 2 rue Gaston Crémieux, F-91000 Evry, France; National Life Science Supercomputing Center, Technical University of Denmark, Building 208, DK-2800 Kongens Lyngby, Denmark; Novo Nordisk Foundation Center for Protein Research, Faculty of Health and Medical Sciences, University of Copenhagen, Blegdamsvej 3B, 2200 København, Denmark; Novo Nordisk Foundation Center for Protein Research, Faculty of Health and Medical Sciences, University of Copenhagen, Blegdamsvej 3B, 2200 København, Denmark; Department of Health Technology, Ørsteds Plads, Building 345C, DK-2800 Kongens, Lyngby, Denmark; CNRS, UMS 3601, Institut Français de Bioinformatique, IFB-core, 2 rue Gaston Crémieux, F-91000 Evry, France; L'institut du Thorax, INSERM, CNRS, University of Nantes, 44007 Nantes, France; Department of Biochemistry and Molecular Biology and VILLUM Center for Bioanalytical Sciences, University of Southern Denmark, Campusvej 55, 5230 Odense, Denmark; CNRS, UMS 3601, Institut Français de Bioinformatique, IFB-core, 2 rue Gaston Crémieux, F-91000 Evry, France; Département de Biologie, Aix-Marseille Université (AMU), 3 place Victor Hugo, 13003 Marseille, France; Computational Biology Unit, Department of Informatics, University of Bergen, N-5008 Bergen, Norway; CNRS, UMS 3601, Institut Français de Bioinformatique, IFB-core, 2 rue Gaston Crémieux, F-91000 Evry, France; Hub de Bioinformatique et Biostatistique–Département Biologie Computationnelle, Institut Pasteur, USR 3756, CNRS, Paris 75015, France

**Keywords:** Bioinformatics software, Tools, Standards, Data sharing

## Abstract

**Background:**

Life scientists routinely face massive and heterogeneous data analysis tasks and must find and access the most suitable databases or software in a jungle of web-accessible resources. The diversity of information used to describe life-scientific digital resources presents an obstacle to their utilization. Although several standardization efforts are emerging, no information schema has been sufficiently detailed to enable uniform semantic and syntactic description—and cataloguing—of bioinformatics resources.

**Findings:**

Here we describe biotoolsSchema, a formalized information model that balances the needs of conciseness for rapid adoption against the provision of rich technical information and scientific context. biotoolsSchema results from a series of community-driven workshops and is deployed in the bio.tools registry, providing the scientific community with >17,000 machine-readable and human-understandable descriptions of software and other digital life-science resources. We compare our approach to related initiatives and provide alignments to foster interoperability and reusability.

**Conclusions:**

biotoolsSchema supports the formalized, rigorous, and consistent specification of the syntax and semantics of bioinformatics resources, and enables cataloguing efforts such as bio.tools that help scientists to find, comprehend, and compare resources. The use of biotoolsSchema in bio.tools promotes the FAIRness of research software, a key element of open and reproducible developments for data-intensive sciences.

## Background

Workers in the life sciences must routinely describe, organize, find, understand, compare, select, use, and connect a large and diverse set of analytical tools and data resources. These tasks can benefit greatly from detailed and consistent resource descriptions that are, when available, human-readable and, ideally, machine-readable. Consider for example the following tasks:

T1: A scientist surveying recently published tools in a general scientific area or for a specific computational task, highlighting those that are freely accessible.
T2: A bioinformatician constructing a data analysis pipeline, and searching for tool alternatives that perform a given operation on a specific type of biological data available in a particular format.
T3: A web developer tasked with building a portal to catalogue and promote the software outputs of a scientific community or consortium.
T4: A project manager assessing the software contributions including scientific impact of a particular project, institution, individual, or research grant.
T5: A software developer wishing to contribute to open source software projects, or seeking to claim credit for and promote their own contributions and productions.

These tasks can be challenging owing to a lack of community-agreed standards or best practices to describe life science software and data resources. Even if open source software developers document their code for better (re)usability, the provided information may address different aspects, with different granularity levels. For instance, T1 would require the tool publication date, as well as its usage license, to be available and machine-readable. In practice, a common strategy is to manually search and browse a large variety of web pages, ranging from software-oriented resources (e.g., GitHub) to scientific literature resources (e.g., PubMed), sometimes through specific form-based search engines. Survey tasks are time consuming and often require repeated and sometimes complex searches. As for T2, searching for tool alternatives is also challenging. In the best cases, software developers/providers precisely describe their contributions. But it is often difficult to compare 2 tools for a similar data analysis task because of the heterogeneity of their description. This would require a tool catalogue (T3) allowing, e.g., tools to be filtered on the basis of their application domain or the type of data processing they provide. Other issues arise when claiming credit for software contributions (T5) or more generally evaluating scientific impact (T4). Citation recommendations are often provided as a paragraph in a tool's documentation, or using the structured Citation File Format (CFF) [1]. Automated retrieval of such citation recommendations would be particularly useful in the context of virtual research environments where life scientists combine bioinformatics tools into data-intensive workflows.

All of these tasks depend on the availability of a shared human-understandable and machine-processable controlled vocabulary and syntax to precisely describe bioinformatics software and data resources. We thus propose biotoolsSchema. Our objective is 2-fold: (i) to provide a technical means to formalize and express rich bioinformatics resource metadata required to achieve at least tasks T1–5 and (ii) to provide incentives for bioinformatics resource providers to enrich their tool metadata for better human/machine accessibility, readability, and reusability. biotoolsSchema is a formalized information model that puts the description of a broad range of bioinformatics resources on a rigorous and consistent syntactic and semantic basis. Our model is developed through a community effort and has evolved steadily since its origin in the BioMedBridges project (concluding in 2015) [2], and more recently during its development for ELIXIR [3], resulting in the latest stable version 3.3.0. In the Comparison to Related Efforts section we introduce and compare biotoolsSchema to various relevant software metadata initiatives, in the context of providing stable solutions to maintain FAIR principles (Findable, Accessible, Interoperable, Reusable) [4] between software providers and consumers.

biotoolsSchema is broadly applicable but optimized to describe bioinformatics tools*—*application software with well-defined data-processing functions (inputs, outputs, and operations). This includes simple tools with 1 or a few closely related functions, and complex, multimodal tools with many functions, available for immediate use as online services, or in a form that users can download, install, configure, and run themselves.

biotoolsSchema defines 50 scientific, technical, and administrative attributes. It concentrates on salient common features, necessary and sufficient for the systematic cataloguing and use of tool information in a variety of contexts. Internally, the EDAM ontology [5] enables rigorous and consistent description of tool functionalities (see “Model of tool function”), such that tools can be readily found, comprehended, and compared by typical software end-users.

biotoolsSchema is available as XML Schema (XSD) and JSON Schema variants and can be used to validate corresponding tool descriptions in XML, JSON, and YAML formats. We summarize the design, methods, and implementation of biotoolsSchema, comparing it to complementary approaches. We also summarize its applications, including the description of a dataset of >17,000 tools registered in the bio.tools registry [6, 7].

## Findings

### Scope

biotoolsSchema is applicable to a nearly complete range of application software, including command-line tools, scripts, libraries, workflows, web applications, database portals, web APIs, web services, SPARQL end points, desktop applications, plug-ins, workbenches, and suites. These tool types and their definitions were settled following an analysis of bio.tools and are included as a controlled vocabulary within biotoolsSchema (see section Controlled vocabularies). They are intended to provide a practical and intuitive designation. In principle, when describing a tool, 1 or more tool types may be assigned, reflecting the different facets of the software being described. biotoolsSchema includes general attributes such as tool description, publication, and license. Execution-layer information, e.g., command-line tool options or web service end points, are out of scope. biotoolsSchema thus complements, e.g., Galaxy [8], the Common Workflow Language (CWL) [9], or Boutiques [10] command-line tool descriptions, and OpenAPI descriptions of web services.

### Software attributes

**Figure 1:**
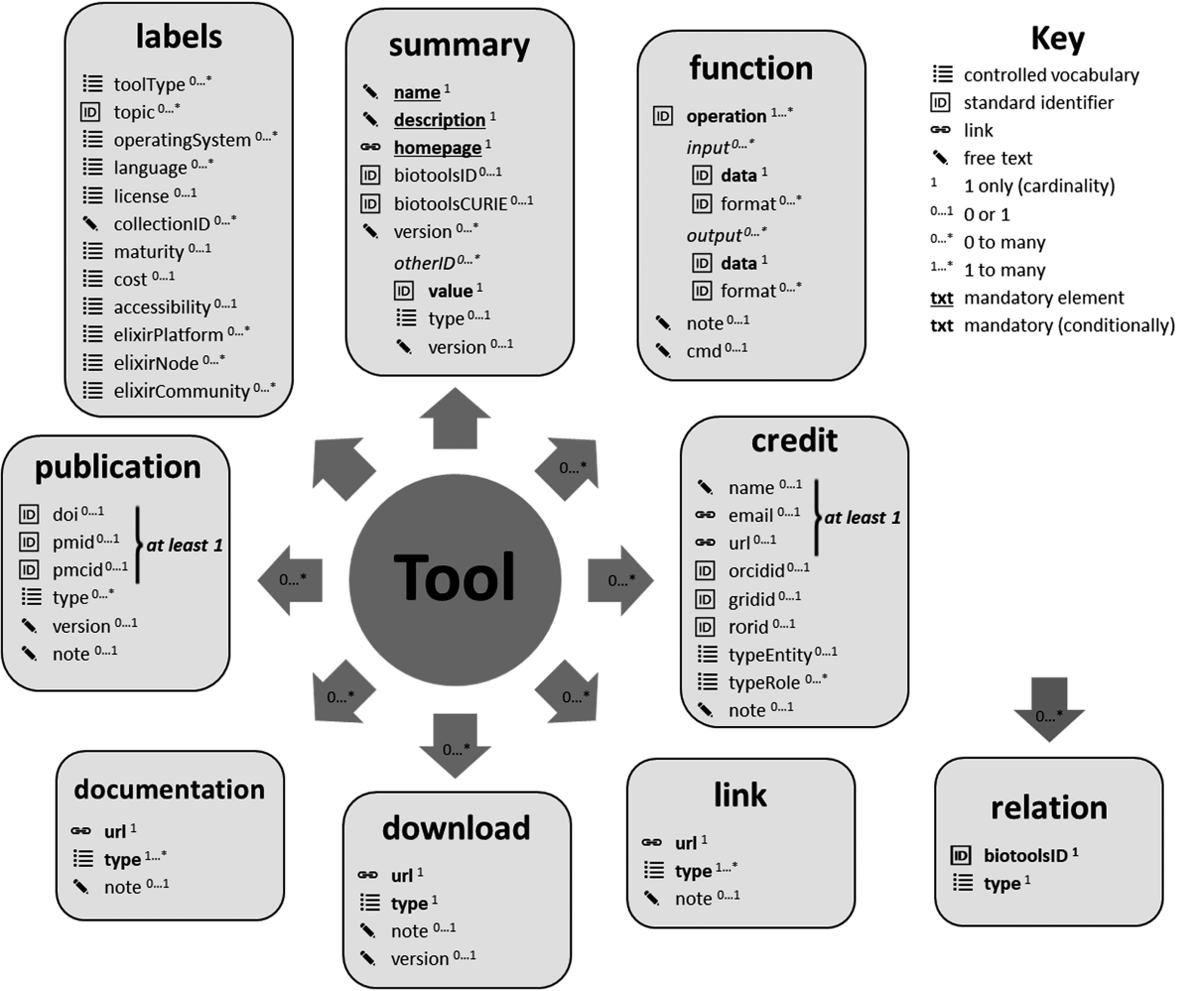
biotoolsSchema overview. Software attributes are organized into 9 groups (in boxes) and include terms from controlled vocabularies defined internally within biotoolsSchema, standard identifiers (including from the EDAM ontology), links, or free text. Cardinality of the groups and attributes is shown in superscript and in the block arrows.

biotoolsSchema covers a total of 50 scientific, technical, or administrative software attributes, organized for convenience into 9 logical groupings (Fig. 1, Table 1). To support the broadest range of applications, only bare-bones metadata (name, short description, and home page) are mandated, the rest of the attributes (Table 2) being conditionally required or optional. The small mandatory core of elements was also a practical necessity for the curation of bio.tools at a very large scale, to facilitate the registration of new entries that can be subsequently improved by the author or the broader community. Element cardinality constraints (1 only, 1 to many, 0 or 1, 0 to many) were chosen to provide flexibility, where applicable. To enable concise information, standard identifiers are used where possible, e.g., digital object identifiers (DOIs) for publications, Open Researcher and Contributor IDs (ORCIDs) for people [11], ontology concept IDs for specialized scientific aspects, and controlled vocabularies for other attributes (see section Controlled vocabularies). Verbose information, e.g., software documentation, terms of use, or citation instructions, is referred to by URL. Regular expression patterns are defined on all applicable elements to support precise syntax validation.

**Table 1:** Software attribute groups

Group	XML element	Description
Summary		Basic information about the software
Labels		Miscellaneous scientific, technical, and administrative details of the software, expressed in terms from controlled vocabularies
Functions	Function	Details of the function(s) (i.e., modes of operation) that the software provides, expressed in concepts from the EDAM ontology
Links	Link	Miscellaneous links for the software, e.g., repository, issue tracker, or mailing list
Downloads	Download	Links to downloads for the software, e.g., source code, virtual machine image, or container
Documentation	documentation	Links to documentation about the software, e.g., user manual, API documentation, or training material
Relationships	relation	Details of a relationship this software has to other software registered in bio.tools
Publications	publication	Publications about the software
Credits	credit	Individuals or organizations that should be credited or can be contacted about the software

Software attributes are grouped within biotoolsSchema. The groups correspond to XML elements with the exception of Summary and Labels groups.

**Table 2:** Software attributes

XML element/JSON property	Description	Type	Cardinality
**Summary group**			
Name	Canonical software name assigned by the software developer or service provider	String	1 only
description	Textual description of the software	String	1 only
homepage	Home page of the software, or some URL that best serves this purpose	URL	1 only
biotoolsID	Unique ID (case insensitive) of the tool that is assigned upon registration of the software in bio.tools, normally identical to tool name	bio.tools tool ID	0 or 1
biotoolsCURIE	bio.tools CURIE (compact URI) based on the bio.tools tool ID	URI	0 or 1
version	Version information (typically a version number) of the software applicable to this bio.tools entry	String	0 or more
otherID	A unique identifier of the software, typically assigned by an ID assignment authority other than bio.tools		0 or more
otherID→value	Value of tool identifier	String	1 only
otherID→type	Type of tool identifier	Enum	0 or 1
otherID→version	Version information (typically a version number) of the software applicable to this identifier	String	0 or 1
**Labels group**			
toolType	A type of application software: a discrete software entity can have >1 type	Enum	0 or more
Topic	General scientific domain the software serves or other general category	EDAM Topic	0 or more
operatingSystem	The operating system that is supported by a downloadable software	enum	0 or more
Language	Name of programming language, e.g., used for the software source code or compatible with an API	enum	0 or more
License	Software or data usage license	enum	0 or 1
collectionID	Tag for a collection that the software has been assigned to within bio.tools	string	0 or more
Maturity	How mature the software product is	enum	0 or 1
Cost	Monetary cost of acquiring the software	enum	0 or 1
accessibility	Whether there are non-monetary restrictions on accessing an online service	enum	0 or 1
elixirPlatform	ELIXIR platform that is credited for developing or providing the software	enum	0 or more
elixirNode	ELIXIR node that is credited for developing or providing the software	enum	0 or more
elixirCommunity	Name of relevant ELIXIR (or associated) community	enum	0 or more
**Function (0 or more)**			
operation	The basic operation(s) performed by this software function	EDAM Operation	1 or more
input|output	Details of primary input/output		0 or more
input|output→data	Type of primary input or output data	EDAM Data	1 only
input|output→format	Allowed format(s) of the input or output data (EDAM Format)	EDAM Format	0 or more
Note	Concise comment about this function, if not apparent from the software description and EDAM annotations	string	0 or 1
Cmd	Relevant command, command-line fragment, or option for executing this function/running the tool in this mode	string	0 or 1
**Link (0 or more)**			
url	A link of some relevance to the software	URL	1 only
Type	The type of data, information, or system that is obtained when the link is resolved	enum	1 or more
Note	Comment about the link	string	0 or 1
**Download (0 or more)**			
url	Link to download (or repository providing a download) for the software	URL	1 only
Type	The type of data, information or system that is obtained when the link is resolved	enum	1 only
Note	Comment about the download	string	0 or 1
version	Version information (typically a version number) of the software applicable to this download	string	0 or 1
**Documentation (0 or more)**			
url	Link to documentation on the web for the tool	URL	1 only
Type	Type of documentation that is linked to	enum	1 or more
Note	Comment about the documentation	string	0 or 1
**Relation (0 or more)**			
biotoolsID	bio.tools ID of an existing bio.tools entry to which this software is related	bio.tools tool ID	1 only
Type	Type of relation between this and another registered software	enum	1 only
**Publication (0 or more)**			
doi*	Digital Object Identifier of a publication about the software (≥1 of DOI, PMID, or PMCID must be specified)	doi	0 or 1*
pmid*	PubMed Identifier	pmid	0 or 1*
pmcid*	PubMed Central Identifier	pmcid	0 or 1*
Type	Type of publication	enum	0 or more
Version	Software version information (typically number) applicable to this publication	string	0 or 1
Note	Comment about the publication	string	0 or 1
**Credit (0 or more)**			
name*	Name of the entity that is credited (≥1 of name, e-mail, or url must be specified)	string	0 or 1*
email*	E-mail address	email address	0 or 1*
url*	URL, e.g., home page of an institute	URL	0 or 1*
orcidid	Unique identifier (ORCID iD) of an entity that is credited	ORCID iD	0 or 1
gridid	Unique identifier (GRID ID) of an organization that is credited	GRID ID	0 or 1
rorid	Unique identifier (ROR ID) of an organization that is credited	ROR ID	0 or 1
fundrefid	Unique identifier (FundRef ID or Funder ID) of a funding organization that is credited	FundRef ID	0 or 1
typeEntity	Type of entity that is credited	enum	0 or 1
typeRole	Role performed by the entity that is credited	enum	0 or more
Note	A comment about the credit	string	0 or 1

biotoolsSchema covers 50 general software attributes grouped for convenience. EDAM concepts may be specified by 1 or both of a URI or term. “enum” indicates a controlled vocabulary defined by biotoolsSchema. Attributes of type xs: token or URL include regular expressions for syntax validation, where applicable.

### Scientific concepts

The EDAM ontology [5] provides the core vocabulary for the scientific description of tools including types of data and data identifiers, data formats, operations, and topics. EDAM organizes these concepts into the EDAM Topic, Operation, Data, and Format subontologies. Concepts may be specified by 1 or both of an EDAM concept URI (e.g., [12]) and/or a term (e.g., “Proteomics”)—a preferred label or synonym of a concept from the appropriate EDAM subontology. It is strongly recommended to specify at least the URI because these persistently identify a concept (labels and synonyms can change).

### Model of tool function

**Figure 2:**
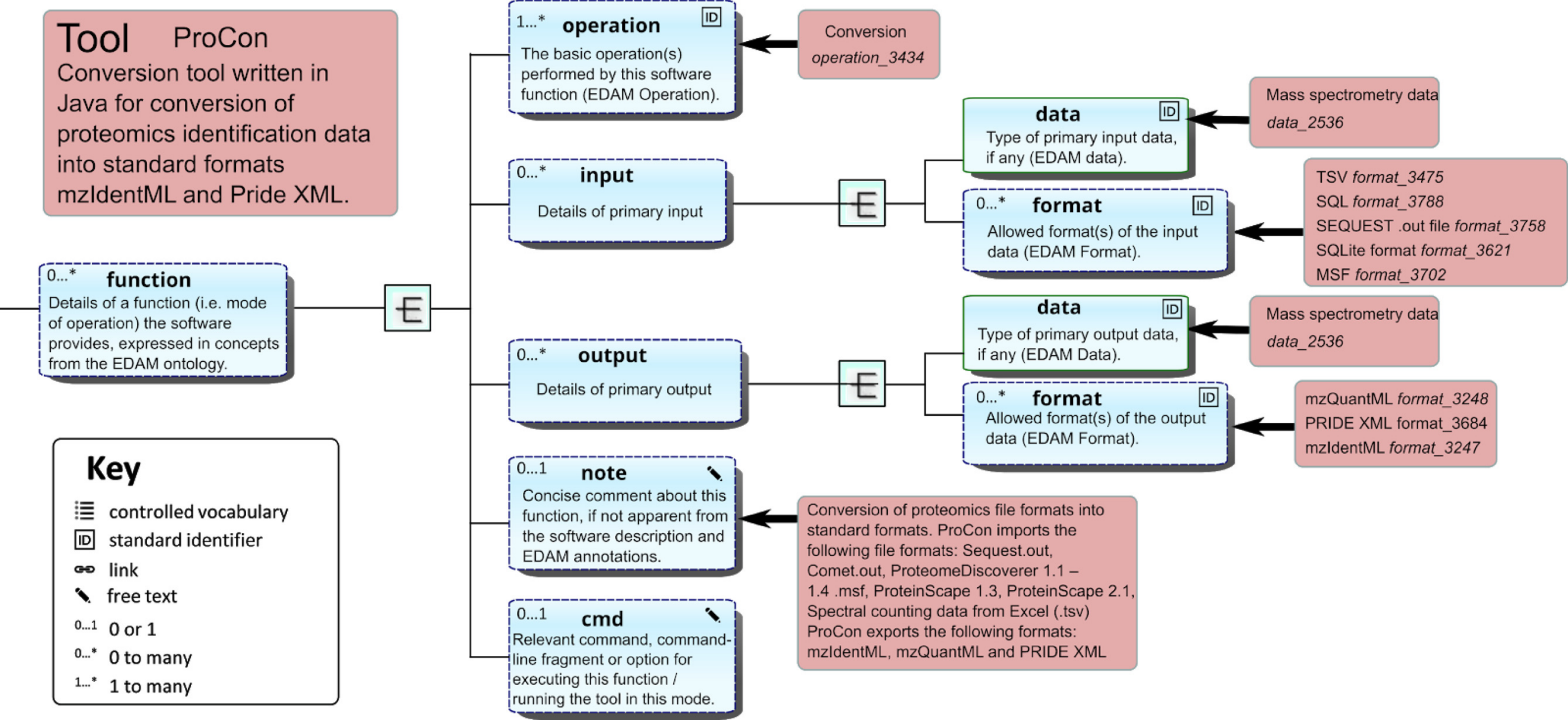
Model of tool function biotoolsSchema follows a simple model of tool function, where each function (mode of operation) performs 1 or more specific operations. Each function may have 1 or more primary inputs and outputs, each of a defined data type and listing supported format(s). Illustration is for the ProCon (biotools:procon) conversion utility.

The model of tool functionality (Fig. 2) is concise and simple. It supports a practical summary of a tool's essential functionality including primary inputs and outputs from the perspective of a typical biologist end-user. Each software entity may have 1 or more functions, each corresponding to a mode of operation that the software provides. In turn, each function performs 1 or more basic operations and has 0 or more primary input and/or output data. Each input or output is of a specified data type and has supported format(s). Operation (e.g., “Sequence alignment"), data type (e.g., “Sequences”), and format (e.g., “FASTA”) are EDAM concepts. An optional comment, and relevant command, command-line fragment, or option for executing the function, may also be specified, mainly to facilitate function identification in tools that can perform multiple operations.

### Auxiliary information

**Figure 3:**
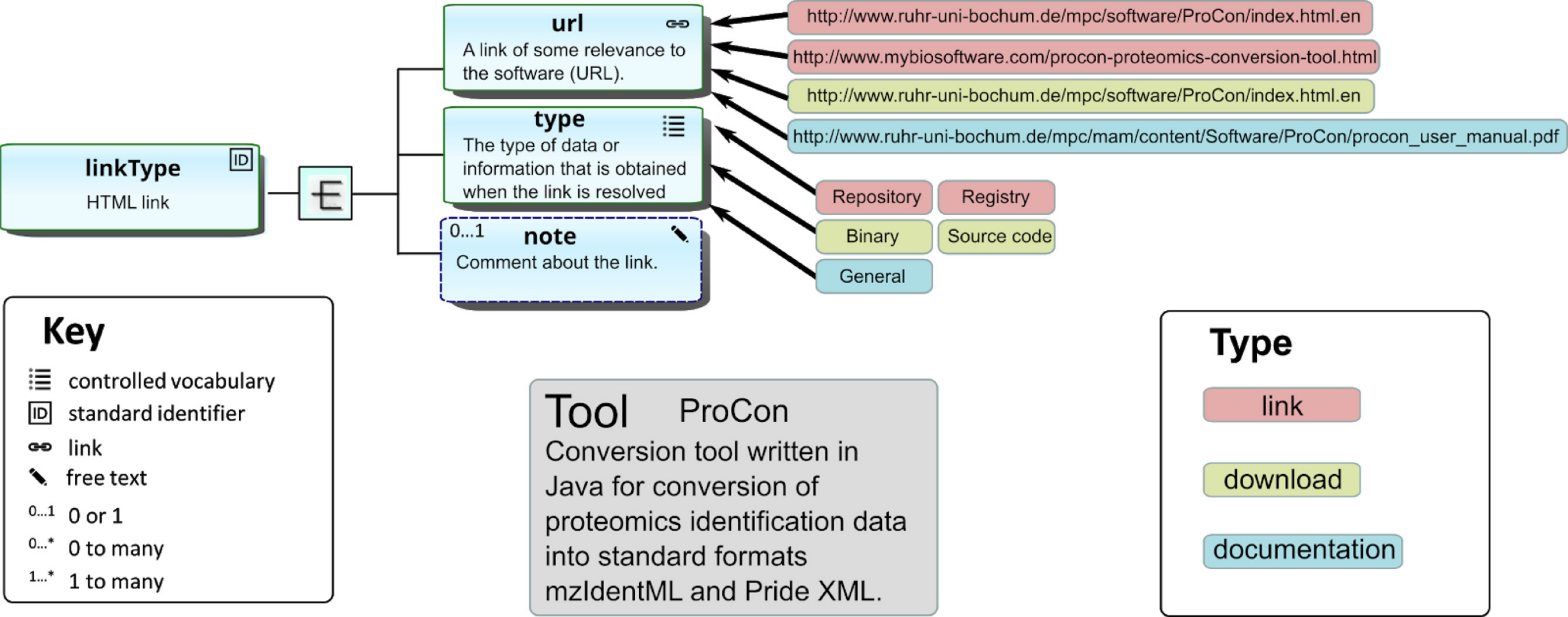
Model of links, downloads, and documentation. Links, downloads, and documentation are modelled in a common way: a URL that is annotated to indicate facets (such as issue tracker or code repository) and an optional comment. Additionally, downloads also allow for associated version information. Illustration is for miscellaneous links for the ProCon (biotools:procon) conversion utility.

Miscellaneous links, downloads, and documentation are modelled in a common way (Fig. 3) including a URL, a type, and an optional comment. Specifying the types of documentation, etc., via controlled vocabularies allows these to be extended in the future, in a way that is non-breaking to schema dependencies.

### Publications

Relevant publications must be specified by (at least) 1 of a DOI, PMID (PubMed reference number), or PMCID (PubMed Central reference number) and may be optionally typed, e.g., “Review.” Use of DOIs—the most generic of these identifiers—is recommended.

### Tool relationships

Relationships between tools that have been registered in bio.tools may be specified by biotoolsID and a term from a controlled vocabulary, which is currently limited to isNewVersion/hasNewVersion (version relationships), uses/usedBy (general functional association), and includes/includedIn (primarily for associating collections such as software suites with their constituent tools). These relationship types will be extended in due course.

### Credits and contact information

Credits and contacts for a tool are handled by a consolidated mechanism. Creditable or contactable entities of various types (e.g., “Person,” “Institute”) and roles (e.g., “Developer,” “Support”) must have ≥1 of a name, e-mail, and URL. ORCIDs provide a persistent reference to information on an individual person. Global Research Identifier Database Identifiers (GRID IDs) and Research Organization Registry Identifiers (ROR IDs) are used for organizations, and Crossref Funder Registry Identifiers (FundRef/Funder IDs) for funding organizations. Specification of these IDs, where available, is strongly recommended because these enable sustainable maintenance and reuse of relevant metadata.

### Controlled vocabularies

In addition to EDAM, a further 18 controlled vocabularies (Table 3) catering for technical aspects are defined internally within biotoolsSchema as "standardized enumerations of terms." Notably the license controlled vocabulary uses identifiers from the industry standard SPDX list [13]. Comprehensive documentation (see Documentation section) including definitions of terms in each vocabulary is available online [14] and is embedded in the chema file (XSD variant only).

**Table 3:** Controlled vocabularies

Controlled vocabulary (#terms)	Description
Identifier type (4)	The type of tool identifier, e.g., “doi”
Tool type (15)	The type of application software, e.g., “Command-line tool”
Operating system (3)	The operating system supported by a downloadable software package, e.g., “Linux”
Programming language (57)	Name of programming language the software source code was written in, e.g., “C”
License (326)	Software or data usage license, e.g., “GPL-3.0”
Maturity (3)	How mature the software product is, e.g., “Mature”
Cost (3)	Monetary cost of acquiring the software, e.g., “Free of charge”
Accessibility (3)	Whether there are non-monetary restrictions on accessing an online service, e.g., “Open access”
Elixir platform (5)	ELIXIR research infrastructure technical platform, e.g., “Tools”
Elixir node (22)	ELIXIR research infrastructure national node, e.g., “France”
Elixir community (11)	Name of relevant ELIXIR (or other) community, e.g., “Galaxy”
Link type (12)	The type of data, information, or system that is obtained when the link is resolved, e.g., “Helpdesk”
Download type (18)	Type of download that is linked to, e.g., “Source code”
Documentation type (15)	Type of documentation that is linked to, e.g., “API documentation”
Publication type (6)	Type of publication, e.g., “Review”
Relation type (6)	Type of tool relationship, e.g., “uses”
Credit entity type (6)	Types of entities that may be credited, e.g., “Person”
Credit entity role (7)	Roles that may be assigned to creditable entities, e.g., “Developer”

biotoolsSchema defines 18 controlled vocabularies catering for technical aspects of software description.

### Implementation of biotoolsSchema in bio.tools

bio.tools [7] provides the means—manually via GUIs and programmatically via a REST API—for a user to browse and search over biotoolsSchema-formatted data and to add to, edit, or download the registry content. Tool description data registered or downloaded via the REST API in a choice of serialization formats (XML, JSON, or YAML) are compatible with biotoolsSchema. bio.tools provides unique, persistent, and immutable tool identifiers (e.g., “signalp,” biotools:signalp). These identifiers are used in persistent bio.tools URLs (e.g., [15]), resolving to Tool Cards, which summarize essential tool information. The bio.tools compact URIs (e.g., “biotools:signalp”) are a convenient short form—simply the identifier in the “biotools” namespace. biotoolsSchema supports other types of identifier, and software version information may be attached to the entire tool description, or to a specific identifier, download, or publication, and specified in a flexible way allowing, e.g., a single version label or a list or range of labels to be annotated. In case a single label annotation reflects a rigorous assignment of software version made by the tool developer, this can be used in conjunction with the bio.tools tool ID to uniquely identify a particular software artefact.

As of September 2020, bio.tools includes 17,370 entries and a total of 301,956 individual annotations, including 101,517 references to concepts from the EDAM ontology, as per attributes defined within biotoolsSchema. Individual tool descriptions vary in richness and are being progressively improved, through an initiative that engages the community with the curation process [16, 17], e.g., producing a high-quality tool description corpus for proteomics data analysis [18]. The bio.tools content, user interfaces, and API will be described in more detail in a future publication.

### Serialization formats and transformations

**Figure 4:**
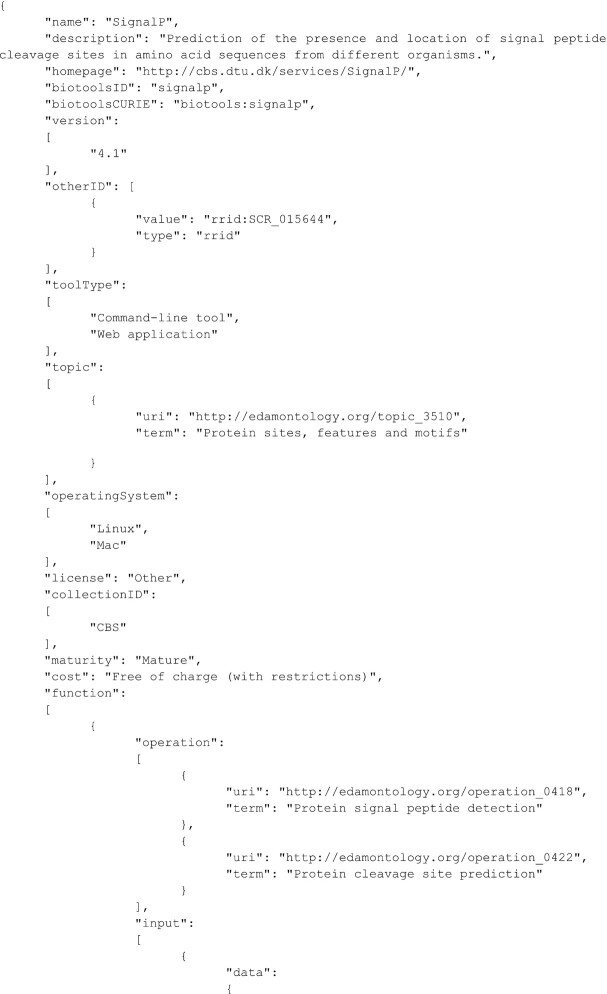

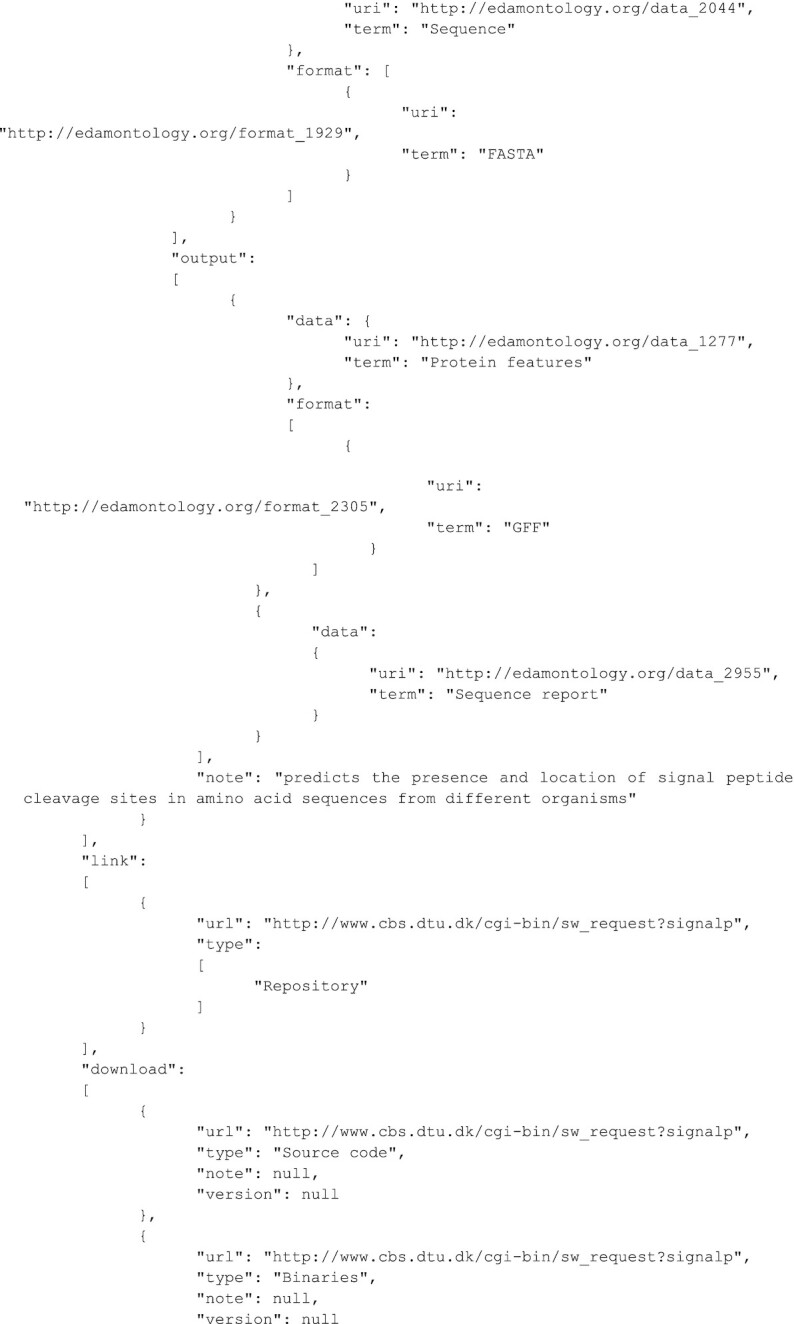

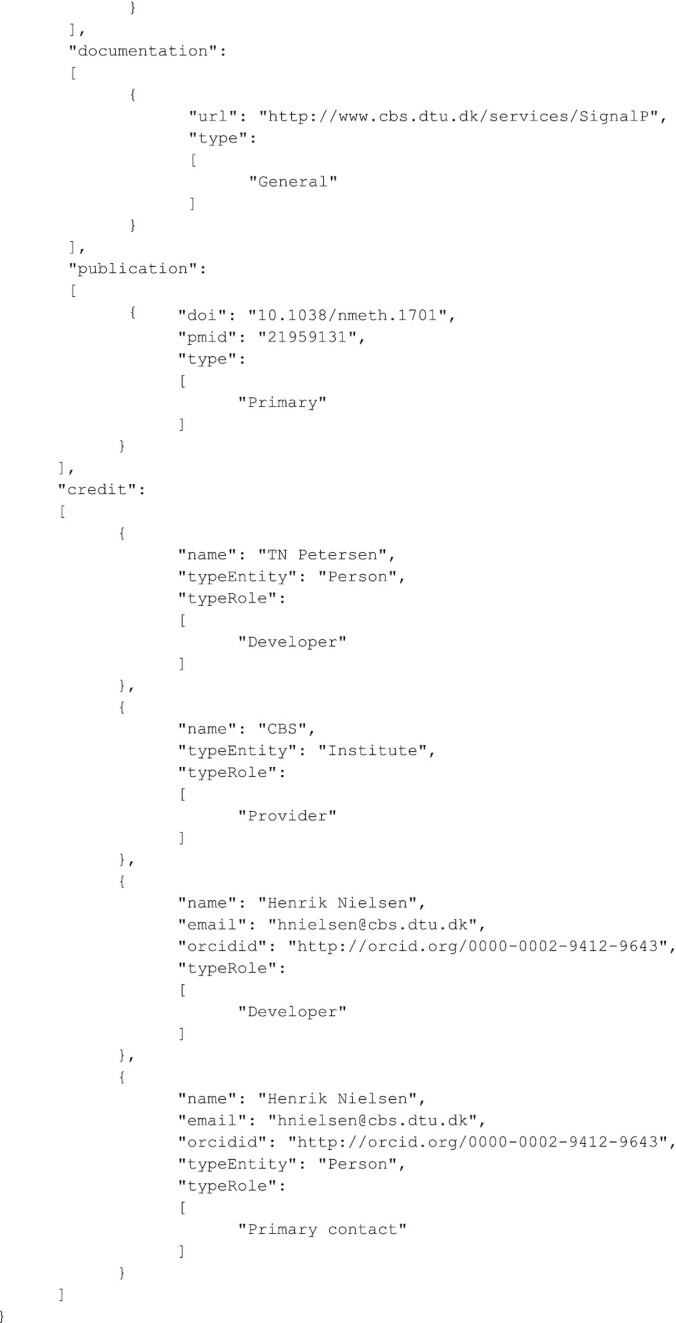
Sample JSON file for signalp tool.

bio.tools supports upload and download of biotoolsSchema-formatted data in a choice of serialization formats (XML, JSON, or YAML). XML support in bio.tools was developed using XSLT transformations to support 2-way, lossless interconversions between biotoolsSchema-formatted XML files and the JSON, YAML, and generic XML formats that are natively supported by the Django web framework used by bio.tools. This offers maximum flexibility to providers and consumers of biotoolsSchema-formatted data, allowing for rigorous validation (against the XSD) irrespective of favoured format. The transforms are freely available [19]. For illustration purposes, a sample JSON file for the SignalP command-line tool (biotools:signalp) (Fig. 4) is shown. We also recently created conversion code to support the lightweight JSON-LD format of the Bioschemas [20] Tool profile, which is now available through the bio.tools API.

## Comparison to Related Efforts

Various research infrastructure or community-led initiatives (Table 4) have defined, or are in the process of defining, sets of information fields to describe bioinformatics software application metadata. The Health Care and Life Sciences (HCLS) Community Profile [21] was an early effort of the Semantic Web Health Care and Life Sciences Interest Group [22]. It specifies dataset descriptions using the Resource Description Framework (RDF), using 24 core metadata elements, and recommends reuse of various well-established, general-purpose RDF controlled vocabularies including Dublin Core [23], Friend-of-a-Friend [24], and PROV [25]. We will describe in a future publication biotoolsRDF [26], an application ontology that defines the OWL2 Web Ontology Language encoding of biotoolsSchema. Like the HCLS Community Profile, it reuses other well-established vocabularies wherever possible, but provides a richer set of attributes and is specifically geared towards application software metadata rather than datasets in general. Tangential to these is the Schema.org vocabulary, founded by the major web search engine providers. It is an exhaustive controlled vocabulary used to annotate contents of web pages and is organized into a hierarchy of a broad range of conceptual classes. It includes concepts relevant to software such as SoftwareApplication and CreativeWork and is well suited for general-purpose, lightweight markup of web pages for discovery purposes. This is in contrast to biotoolSchema, which is tailored specifically to detailed descriptions of application software especially, and mandates stricter syntax and semantics. A Tool Profile [27] currently under development for the Bioschemas project [20] will provide guidelines on the consistent adoption of Schema.org markup for the description of software tools in life sciences, including, e.g., recommending the use of EDAM ontology for scientific aspects. The emerging Tool Profile is fully compatible with biotoolsSchema, and we have implemented it in bio.tools, which now serves via the API a Bioschemas serialization of the bio.tools content.

**Table 4:** Software metadata initiatives

Initiative	Description
HCLS Community Profile	Specification for dataset description using RDF [21]
eInfraCentral Service Description Template	Information model for European eInfrastructure services (including software services) [28]
DataCite Metadata Schema	XML schema and guidelines of core metadata properties for resource identification, citation, and retrieval [29]
OpenAIRE Application Profile	Guidelines for software repository managers [30]
CodeMeta Metadata Crosswalk	Vocabulary for software metadata concepts and crosswalk between software metadata projects [31]
Schema.org Vocabulary	Controlled vocabulary for marking up web pages [32]
Bioschemas Tool Profile	Schema.org specification for tools in the life sciences [33]
FORCE11 Software Citation Principles	Basic metadata requirements for software citation [34]

Various initiatives for software metadata of relevance to biotoolsSchema are described.

Parallel to initiatives depending upon Semantic Web technologies are efforts reflecting existing practice or the requirements of various research infrastructures. CFF [1] is a YAML format for general-purpose software annotations. Its focus is to support all citation-specific use cases for the citation of software and thus promote attribution and credit of research software. It provides more detail in this area than biotoolsSchema but lacks attributes, e.g., around tool functionality, which are a focus of biotoolsSchema. The DataCite Metadata Schema from DataCite [35]—a non-profit organization that provides persistent identifiers (DOIs) for research data—includes core metadata properties primarily for resource identification, citation, and retrieval, encapsulated in an XML schema with usage guidelines. The Application Profile included in the Guidelines for Software Repository Managers from OpenAIRE [36]—a European project supporting Open Science—is based on DataCite and covers 23 software attributes, primarily to make software products citable. Similarly, the Software Citation Principles produced by FORCE11 community initiative [37] define 11 basic metadata requirements for software citation. The SciCrunch registry [38] shares a similar purpose to bio.tools but uses an RDF-based data model. Their scope is similar, but biotoolsSchema enables, through its use of EDAM, the end-user to drill down to fine-grained aspects of tool functionality such as specific inputs, outputs, and operations. SciCrunch uses persistent Research Resource Identifiers (RRIDs) [39], which in case of computational tools are equivalent to bio.tools tool IDs. The eInfraCentral Service Description Template produced by the European E-Infrastructure Services Gateway, eInfraCentral [40], is (as a work in progress) defining the information requirement for a common E-Infrastructures service catalogue, which will describe and offer services to end-users in a harmonized way, through the European Open Science Cloud (EOSC) portal [41]. ELIXIR is involved in the Tools Collaboratory for the EOSC-Life project [42], which will drive the development of an environment enabling the cloud deployment of workflows for the analysis and integration of life science data. We anticipate that biotoolsSchema-formatted descriptions of tool functionality will contribute to this environment, especially to workflow composition and the production of applicable registries.

The CodeMeta specification [43] is a more generalized approach and was developed as a lightweight format to describe scientific software, based on an extension of Schema.org using JSON-LD. A major component is the CodeMeta Metadata Crosswalk, produced by the CodeMeta community project [44], which is a table reflecting a comparison of software metadata used across multiple code repositories and systems. The crosswalk (a work in progress) yields an exhaustive set of 65 software metadata concepts (mostly mapped to Schema.org concepts) and can inform efforts to produce a minimal concept vocabulary for software reflecting a consensus in the mappings. biotoolsSchema has been submitted as a CodeMeta crosswalk (see below) and covers many of the common concepts, despite these not always being explicitly mapped owing to technical limitations of how the crosswalk is currently represented.

This summary of initiatives is not exhaustive. Others include the DOE CODE initiative [45] for code archiving by the U.S. Department of Energy (DOE), guidelines [46] for rich search results for software from Google, and specialized ontologies for software including SWO [47] and OntoSoft [48], each serving a different use case. We have thus a plethora of different recommendations and ways to annotate and share software metadata. The diversity reflects a wide range of perspectives, use cases, and contexts but brings the challenge of curating software metadata and sharing it between systems whilst avoiding inconsistencies and duplication of efforts. We ameliorate this interoperability issue, at least so far as sharing and reusing bio.tools metadata, through an exhaustive crosswalk (Table 5) between biotoolsSchema elements and key software metadata initiatives including CodeMeta, Schema.org, OpenAire, DataCite, HCLS, eInfraCentral, FORCE11, and miscellaneous RDF vocabularies. Each element in biotoolsSchema was mapped, where possible, to the corresponding field used by these initiatives, and the mappings aggregated, discussed, and reviewed, resulting in a consolidated crosswalk (Table 5) for biotoolsSchema, which has been submitted to the CodeMeta. The crosswalk thus provides a framework useful to any engineer integrating software metadata provided in these contexts.

**Table 5:** Comparison of biotoolsSchema and other software metadata initiatives

bio.tools	CodeMeta/Schema.org 36/65^1^	OpenAIRE 18/23^2^	DataCite 11/19^2^	Miscellaneous RDF vocabularies	HCLS 12/24^3^	eInfraCentral 23/24^4^	FORCE11 10/11
**Summary**							
name	name	Name	Title	rdfs:label, dct:title	Title	Service Name	Software name
description	description	Description	Description	rdfs:comment, dct:description	Description	Service Tagline, Service Description	Description
homepage	url	Landing page (datacite:alternateIdentifier)		foaf:page	HTML page	Service URL, Service Order	
biotoolsID	identifier	Identifier	Identifier	dct:identifier		Service ID	Unique identifier
biotoolsCURIE	identifier	Identifier	Identifier	dct:identifier		(Service ID)	
version	softwareVersion	Version	Version	pav:version pav:hasCurrentVersion pav:previousVersion	Version identifier	Service Version	Version number
otherID	identifier	Alternate identifier	AlternateIdentifier, alternateIdentifierType	rdfs:seeAlso, dct:identifier			
**Function**							
operation				dcat:keyword		Service Tags	
input|output→data				dcat:keyword			
input|output →format				dcat:keyword			
note				rdfs:comment			
cmd				rdfs:comment			
**Labels**							
toolType	applicationSubCategory	Software Type	ResourceType	rdfs:comment		(Service Category, Service Subcategory)	
Topic	keywords	Subject	Subject, valueURI	dcat:keywords	Keywords	Service Tags, Service Coverage	Keywords
operatingSystem	operatingSystem			dct:medium dct:mediator			
language	programmingLanguage	Programming Language (datacite:format)		dct:language	Language		
license	license	License Condition	Rights, rightsURI	dct:licence	License		Software license
collectionID				dct:identifier			
maturity						Service Life Cycle Status^5^	
cost	isAccessibleForFree^6^					Service Cost	
**Link**							
any type	relatedLink						
“Repository”	codeRepository	Repository (datacite:publisher)					Location/repository
“Helpdesk”						Service Helpdesk, Service Feedback	
“Mailing list”						Service Feedback	
“Issue tracker”	codemeta:issueTracker						
**Download**							
any type	downloadUrl			dcat:downloadURL, prov:atLocation			
“Source code,” “Software package” or “Binaries”		Distribution location (datacite:alternateIdentifier)		dcat:mediaType			
‶Icon”					Logo	Service Symbol	
“Screenshot”						Service Multimedia	
**Documentation**							
‶General″	codemeta:readme	Documentation (datacite:alternateIdentifier)		dcat:landingPage	Documentation		
‶User manual″	softwareHelp					Service User Manual	
‶Terms of use″		Access Rights (datacite:rights)	Rights, rightsURI	dct:rights	Rights	Service Terms of Use	
‶Training material″						Service Training Information	
**Publication**							
doi|pmid|pmcid	referencePublication	-	-	dct:references	References		Index citations
**Credit**							
name	givenName, familyName, affiliation, name		creatorName, givenName, familyName, affiliation,	foaf:name		,	
email	email			foaf:mbox			
url	url			foaf:page prov:atLocation			
orcidid	identifier		nameIdentifier	dct:identifier			
typeEntity			nameType				
typeRole				foaf:providedBy pav:createdBy pav:authoredBy pav:curatedBy pav:contributedBy			
typeEntity == ‶Funding agency″	funder, codemetada:funding	Funding Reference	funderName, FundingReference			Service Funding	
typeRole == ‶Developer″	author, creator	Author	Creator	dct:creator	Creators		Author(s)
typeRole == ‶Contributor″	contributor, editor		Contributor				Contributor role
typeRole == ‶Provider″	provider, producer					Service Provider Name	
typeRole == ‶Maintainer″	codemeta:maintainer						
typeRole == ‶Primary contact″		Contact Person, Contact Group		dct:contributor	Contributors		

Elements in biotoolsSchema are mapped to equivalent elements from various software metadata initiatives. Only those elements that could be mapped are shown.Numbers divides by slash in the header row e.g. 36/65 indicate the proportion of all elements in a metadata initiative that were mapped.

1. Fifty-five of which are Schema.org properties and 10 of which are specific to CodeMeta.

2. Disregarding DataCite subproperties.

3. HCLS core metadata elements.

4. Disregarding service level targets and performance information (out of biotoolsSchema scope).

5. eInfraCentral uses a different but compatible controlled vocabulary.

6. True where cost==”Free of charge”.

## Discussion

The efficiency of workers using scientific software across the spectrum of the life sciences depends, in large part, on high-quality and convenient bioinformatics software metadata. biotoolsSchema, in combination with EDAM, provides a formalized, rigorous, and consistent specification of the syntax and semantics for these metadata. This enables software developers and service providers to define their productions in a consistent way (tasks T4, T5), cataloguers to communicate clearly what is available (tasks T1, T3), and software end-users to more efficiently use these resources (task T2). For example, a recent study [49] demonstrated the usefulness of biotoolsSchema-formatted data for automated workflow composition in mass spectrometry–based proteomics data analysis. Here, biotoolsSchema, through its use of the EDAM ontology, enabled the precise annotation of tool inputs, outputs, and operations that was critical for workflow synthesis. biotoolsSchema thus encompasses diverse use cases, from provenance through to query and discovery, and can help to standardize the curation and exchange of metadata across software projects, repositories, initiatives, and organizations.

In biotoolsSchema, a great complexity of information—including tool functionalities, fields of use, interfaces, deployments, distributions, documentation, and so on—is reduced to a manageable and practical level. The model is applicable to nearly all technical types of tool, and supports the uniform description of key scientific, technical, and administrative attributes. Specifically, in line with tasks T1 and T3 addressing tool surveys and community-oriented registries, it allows for a presentation and comparison of tool information, which often cannot conveniently be obtained from a Google search or cursory inspection of a provider's website. With progressive development, biotoolsSchema applications such as bio.tools will help to make complex tool functions more easily understood and render tools more accessible, usable, and interoperable, i.e., more FAIR [4]. For example, a study [50] showed how formalized tool descriptions could be reused and bridged to workflow provenance to provide user- and machine-oriented data summaries. This complements efforts such as Boutiques [10], which have been applied to the neuroimaging analysis domain. We plan to generically evaluate the FAIRness of tools registered in bio.tools once there is broad community agreement on a suitable set of metrics. For a start, we examined the criteria defined by Lamprecht et al. [51] with respect to the features of bio.tools and biotoolsSchema. This comparison (Table 6) shows how in practice the use of biotoolsSchema through bio.tools already helps to improve the FAIRness of tools. The table also informs possible FAIR metrics, which can be encapsulated using our emerging Tool Information Profile system [52] and used to provide an objective, transparent, and flexible framework to evaluate tool FAIRness.

**Table 6:** Role of bio.tools and biotoolsSchema in the evaluation of software FAIRness

Criterion	FAIR principle for software	Provided by bio.tools	Provided by biotoolsSchema
F1	Software and its associated metadata have a global, unique, and persistent identifier for each released version.	bio.tools assigns persistent and unique identifiers to registered software.	Attributes for bio.tools-specific software identifiers (“biotoolsID” and “biotoolsCURIE”) and other identifiers (“otherID”) including doi, rrid, and cpe.
F2	Software is described with rich metadata.		biotoolsSchema defines >50 important scientific, technical, and administrative attributes that support cataloguing, discovery, use, and interoperability of software.
F3	Metadata clearly and explicitly include identifiers for all the versions of the software they describe.		biotoolsSchema supports the annotation of all versions of the software applicable to a bio.tools entry. Version information can also be attached to specific identifier, download, or publication attributes.
F4	Software and its associated metadata are included in a searchable software registry.	bio.tools information includes all metadata supported by biotoolsSchema.	biotoolSchema supports links to where software source code and binaries can be downloaded.
A1	Software and its associated metadata are accessible by their identifier using a standardized communications protocol.	Metadata can be retrieved from bio.tools using an API.	
A1.1	The protocol is open, free, and universally implementable.	bio.tools API is a fully documented REST API (see [53]).	
A1.2	The protocol allows for an authentication and authorization procedure, where necessary.	Authentication and authorizations management are handled by the REST API (see, e.g., [54]).	
A2	Software metadata are accessible, even when the software is no longer available.	Curation practice for bio.tools is to set the maturity to “legacy” instead of removing an entry; entries are never deleted.	biotoolsSchema supports annotation of software as “legacy” (“maturity” attribute).
I1	Software and its associated metadata use a formal, accessible, shared, and broadly applicable language to facilitate machine readability and data exchange.	Schema.org semantic markup is available through the API.	biotoolsSchema is specified as both XSD and JSON Schema and is compatible with Schema.org.
I2S.1	Software and its associated metadata are formally described using controlled vocabularies that follow the FAIR principles.		biotoolsSchema makes extensive use of controlled vocabularies, all of which are rendered FAIR through ontology portals such as OLS or are publicly available and documented (see Table 3).
I2S.2	Software uses and produces data in types and formats that are formally described using controlled vocabularies that follow the FAIR principles.		biotoolsSchema supports the data consumed and produced by software to be described using the EDAM ontology.
I4S	Software dependencies are documented and mechanisms to access them exist.		biotoolsSchema provides a controlled vocabulary of documentation types (e.g., Installation instructions), which should describe software dependencies in detail. It also provides a controlled vocabulary for describing dependencies between software resources as relationships between tools (using, e.g., “uses” and “usedBy” of ”relation” attribute).
R1.1	Software and its associated metadata have independent, clear, and accessible usage licenses compatible with the software dependencies.	bio.tools entries are available under CC BY-4.0 license.	biotoolsSchema itself is licensed under CC BY-SA 4.0. Individual software licenses are documented by the “license” attribute.
R1.2	Software metadata include detailed provenance; detail level should be community agreed.		biotoolsSchema supports links to the software repository where provenance information such as version history, releases, and contributors should be hosted. biotoolsSchema also includes a detailed credit model, which provides contact details for various types of contributing entities (e.g., Person, Institute) and roles (e.g., Developer, Maintainer).
R1.3	Software metadata and documentation meet domain-relevant community standards.		biotoolsSchema attribute “documentation” supports links to various documentation resources, and specification of documentation type (e.g., “Citation instructions”).

For each FAIRness criterion for software (in column 1) as proposed in [51], the role of bio.tools (column 2) and biotoolsSchema (column 3) are summarized. Some of these criteria, such as the assignment of an identifier, are satisfied by a bio.tools registration, while other criteria such as the license depend upon the curation of a biotoolsSchema attribute within bio.tools.

Our stand-alone schema allows for community development of the model to be loosely coupled to applications such as bio.tools, and provides a means for an end-user to validate content external to any system, ensuring correct syntax, structure, and completeness (T3). biotoolsSchema must evolve to keep pace with developments in the field and support new applications and integration scenarios. This may include richer modelling of the complex relationships between resources, and support for specialized biological ontologies such as Gene Ontology [55] for molecular function, cellular component, and biological process, Sequence Ontology [56] for genomic elements, and NCBI Taxonomy [57] for taxa. biotoolsSchema will thus provide a means to relate a large set of tools such as in bio.tools to a broader and flourishing ecosystem of workflows, databases, and ontologies.

Future changes will be pragmatic, driven by community use cases, and in light of practical experience of what data are useful and readily available. No single model, registry, or initiative can hope to cover all bases. biotoolsSchema can be augmented by (and will not duplicate) the functionalities of related well-maintained models provided by more specialized initiatives, e.g., execution-layer information about command-line tools provided by CWL, or information about service end points supported by OpenAPI [58]. More specifically, we plan to build upon previous work [59–62] to improve the cross-linking and cross-enrichment of execution-oriented tool descriptions with biotoolsSchema data. Volatile attributes, or attributes that must be frequently recalculated, such as metrics of usage, technical performance data, links to similar tools, software dependencies, hardware requirements, and so on, will remain out of scope.

Different software metadata use cases have different information requirements. biotoolsSchema supports very minimal or much more comprehensive information specifications, according to needs, without imposing a high curation burden and thus a barrier to adoption. It provides the basis for, but cannot in itself specify, a flexible information requirement suited to diverse purposes and contexts, e.g., curation of registries such as bio.tools, required information for service delivery plans, publication of software articles, or metrics for software metadata or project quality. For such purposes, a framework [63] for tool information requirements is under development (“Tool Information Profiles”), which is based on biotoolsSchema but goes beyond the syntactic/semantic constraints that can conveniently be defined in XML schema. Human-readable guidelines for curation of software metadata are also being developed as part of an emerging Curators Guide [64].

biotoolsSchema, with progressive development and adoption, can benefit the whole bioinformatics community. It can help to support best practices promoted by various research infrastructures that emphasize the value of bioinformatics software registries for findability [65, 66], and bridge the gap between technology-oriented developers and service-oriented research infrastructures and organizations. The field of software metadata management is socially and technically very complex and includes many more stakeholders and perspectives than are summarized here, with multiple projects serving different but overlapping needs, use cases, and contexts. We encourage all such efforts and warmly welcome collaborations for the continued development of biotoolsSchema, its applications, and integration into the broader bioinformatics ecosystem.

## Methods

### Design considerations

Requirements were established during a series of community-led workshops resulting in 10 founding principles and design considerations, now implemented as characteristics of biotoolsSchema:

- Practical***—***focus on salient attributes of practical value in everyday use, especially to support the discovery, use, and practical interoperability of software; superfluous details are excluded.
- General***—***generally applicable, i.e., to all manner of bioinformatics resources (see Scope).
- Consistent***—***use ontologies and standardized enumerations of terms (see Controlled Vocabularies) where possible, to support precise searches over biotoolsSchema-formatted data and return of consistent and therefore comparable information.
- Concise***—***mandate URLs or standard identifiers where possible, helping to ensure the sustainable upkeep of biotoolsSchema-formatted data and support future integrations, applications, and cross-linking with other resources.
- Simple***—***biotoolsSchema is as flat (unstructured) as is practicable, ensuring ease of use whilst preserving essential structure, e.g., a meaningful model of tool function.
- Compatible***—***it is inevitable that tool providers, integrators, and cataloguers will continue to use a variety of models, methods, and formats for tool descriptions; biotoolsSchema is broadly compatible (see Comparison to Related Efforts) to support future integration scenarios.
- Extensible***—*** to cater for emerging requirements, and remain adaptable by others for their own purposes (see Development process and status).
- Stable***—***the maintenance of software dependencies on mutating schema is expensive. Backwards-incompatible changes are only made if absolutely required (see Development process and status).
- Free and open source***—***to encourage reuse and new applications.
- Community-driven development—to ensure that end-user needs are satisfied.

### Development process and status

The model is an evolution of an early prototype developed for BioMedBridges that began in 2012. Its evolution has been tightly linked with that of EDAM and bio.tools, as these projects together are the key components of an ecosystem [17] that enables communities, projects, and individuals to describe and share their own bioinformatics resources. biotoolsSchema had multiple successive development iterations, most of which were based on community events such as workshops and hackathons (the online documentation of bio.tools includes a non-exhaustive list of such events [67]). Such events typically involved the gathering of developers from the ecosystem described, together with members from domain (e.g., proteomics) or project-specific (e.g., Debian Med) communities. The events allowed us to combine contributions such as the addition of content by domain experts with the collection of feedback and requirements on EDAM, biotoolsSchema, and bio.tools. Whenever possible, we also included downstream activities, including request management using agile techniques such as priority poker, and development and debugging activities.

In parallel, development was informed by growth in bio.tools: major content providers and other end-users have helped to validate the model, with the registry itself providing a valuable dataset for this purpose. Thus, we consider the stable version (3.3.0) to satisfy major community requirements and provide a solid foundation upon which the content, functions, integration, and applications of portals such as bio.tools can be built. The model must be subject to future improvements, and the schema is extensible; both the number and type of attributes can evolve, according to end-user requirements. To provide stability for developers and software dependencies, major changes are restricted to approximately yearly cycles in released stable versions. Future versions will not depart fundamentally from the attributes or structure described in this article. From version 3.0.0, version numbers follow the SemVer 2.0.0 scheme [68]. All developments are tracked at GitHub [69]; feedback, contributions, and collaboration are welcome.

biotoolsSchema is a community-driven project governed under the leadership of the French ELIXIR Node (Jacques van Helden, Joint Head of Node) in collaboration with partners within and beyond ELIXIR, ensuring its sustainability. For further information see [70].

### Documentation

The schema is comprehensively and consistently documented:

- textual (human-readable) description of each schema element
- additional, highly concise element descriptions, suitable, e.g., as tips in user interfaces
- definition of terms in all controlled vocabularies
- mapping of schema elements including controlled vocabularies to other relevant models and vocabularies
- usage information including technical details (such as syntax and use of bio.tools API) and curation guidelines (good practice on using biotoolsSchema to describe tools)
- information about the project and community

The documentation is, where possible, encoded within the XSD and JSON schemas but also made available online in a more user-friendly form:

- [71] (project docs)
- [72] (technical docs)
- [73] (technical docs for the JSON schema variant)
- [74] (bio.tools API user guide)
- [75] (bio.tools curation guide)

## Availability of Source Code and Requirements

biotoolsSchema is licensed under a Creative Commons Attribution-ShareAlike 4.0 International License (CC BY-SA 4.0) [69].

The bio.tools content is freely available to all under the Creative Commons Attribution licence (CC BY-4.0) and can be downloaded from bio.tools [7].

An archival copy of supporting data and documentation for biotools schema is also available via the *GigaScience* repository, GigaDB [76].

## Data Availability

Availability and implementation: https://github.com/bio-tools/biotoolsschema

Supplementary Information: http://biotoolsschema.readthedocs.io/

## Abbreviations

API: Application Programming Interface; DOE: Department of Energy; DOI: digital object identifier; EOSC: European Open Science Cloud; FAIR: Findable, Accessible, Interoperable, Reusable; GRID ID: Global Research Identifier Database Identifier; GUI: graphical user interface; HCLS: Health Care and Life Sciences; JSON: JavaScript object notation; NCBI: National Center for Biotechnology Information; ORCID: Open Researcher and Contributor ID; PMID: PubMed Identifier; PMCID: PubMed Central Identifier; RDF: Resource Description Framework; REST: representational state transfer; ROR ID: Research Organization Registry Identifier; RRID: Research Resource Identifier; URL: Uniform Resource Locator.

## Competing Interests

The authors declare that they have no competing interests.

## Funding

This work was supported by funding from the Institut Français de Bioinformatique (IFB/ELIXIR France), the Danish Ministry of Higher Education and Science (ELIXIR Denmark), and from the European Union's Horizon 2020 research and innovation programme (grant agreement No. 676 559, ELIXIR-EXCELERATE).

## Authors' Contributions

J.I. led the development of the schema, and the article with contributions from all authors. J.I., M.K. and K.R. designed and prototyped the schema. M.K, H.I, A.G., V.S. J.H. and H.M. contributed to the schema development. H.I., E.R., and P.C. implemented the schema in bio.tools.

## Supplementary Material

giaa157_GIGA-D-20-00206_Original_Submission

giaa157_GIGA-D-20-00206_Revision_1

giaa157_Response_to_Reviewer_Comments_Original_Submission

giaa157_Reviewer_1_Report_Original_Submission Gregory Kiar -- 8/6/2020 Reviewed

giaa157_Reviewer_1_Report_Revision_1 Gregory Kiar -- 11/13/2020 Reviewed

giaa157_Reviewer_2_Report_Original_Submission Jeff Christiansen -- 8/6/2020 Reviewed

giaa157_Supplemental_Figures

## Notes

These authors contributed equally to this work.
