# Supplementary material for: biotoolsSchema: a formalized schema for bioinformatics software description
Source: Gigascience. 2021 Jan 27;10(1):giaa157. doi: 10.1093/gigascience/giaa157 (PMC7842104; doi:10.1093/gigascience/giaa157)
Supplement: giaa157_GIGA-D-20-00206_Original_Submission [file giaa157_giga-d-20-00206_original_submission.pdf]

GigaScience

biotoolsSchema : a formalised schema for bioinformatics software description

--Manuscript Draft--

**Manuscript Number:**	GIGA-D-20-00206
**Full Title:**	biotoolsSchema : a formalised schema for bioinformatics software description
**Article Type:**	Technical Note
**Funding Information:**	H2020 European Research Council (676559) — Not applicable<br>Danish Ministry of Higher Education and Science — Dr Jon Ison
**Abstract:**	Life scientists routinely face massive and heterogeneous data analysis tasks, and must find and access the most suitable databases or software in a jungle of web-accessible resources. The diversity of information used to describe life science digital resources presents an obstacle to their utilisation. Although several standardisation efforts are emerging, no information schema has been sufficiently detailed to enable uniform semantic and syntactic description - and cataloguing - of bioinformatics resources. Here we describe biotoolsSchema, a formalised information model which balances the needs of conciseness for rapid adoption, while still providing rich technical information and scientific context. biotoolsSchema results from a series of community-driven workshops, and is deployed in the bio.tools registry, providing the scientific community with more 17,000 machine-readable and human-understandable descriptions of software and other digital life-science resources. We compare our approach to related initiatives and provide syntactic and semantic alignments to foster interoperability and reusability. The work supports the production of FAIR scientific software; a key element of open and reproducible developments for data-intensive sciences.
**Corresponding Author:**	Jon Ison, PhD<br>French Institute of Bioinformatics (IFB-Core)<br>Évry, FRANCE
**Corresponding Author Secondary Information:**	
**Corresponding Author's Institution:**	French Institute of Bioinformatics (IFB-Core)
**Corresponding Author's Secondary Institution:**	
**First Author:**	Jon Ison, PhD
**First Author Secondary Information:**	
**Order of Authors:**	Jon Ison, PhD
	Hans Ienasescu
	Emil Rydza
	Piotr Chmura
	Kristoffer Rapacki
	Alban Gaignard
	Veit Schwämmle
	Jacques van Helden
	Matúš Kalaš
	Hervé Ménager
**Order of Authors Secondary Information:**	
**Additional Information:**	

Powered by Editorial Manager® and ProduXion Manager® from Aries Systems Corporation

Question	Response
Are you submitting this manuscript to a special series or article collection?	No
**Experimental design and statistics**<br><br>Full details of the experimental design and statistical methods used should be given in the Methods section, as detailed in our Minimum Standards Reporting Checklist. Information essential to interpreting the data presented should be made available in the figure legends.<br><br>Have you included all the information requested in your manuscript?	Yes
**Resources**<br><br>A description of all resources used, including antibodies, cell lines, animals and software tools, with enough information to allow them to be uniquely identified, should be included in the Methods section. Authors are strongly encouraged to cite Research Resource Identifiers (RRIDs) for antibodies, model organisms and tools, where possible.<br><br>Have you included the information requested as detailed in our Minimum Standards Reporting Checklist?	Yes
**Availability of data and materials**<br><br>All datasets and code on which the conclusions of the paper rely must be either included in your submission or deposited in publicly available repositories (where available and ethically appropriate), referencing such data using a unique identifier in the references and in the "Availability of Data and Materials" section of your manuscript.	Yes

Powered by Editorial Manager® and ProduXion Manager® from Aries Systems Corporation

Have you have met the above requirement as detailed in our Minimum Standards Reporting Checklist?	

Manuscript

Click here to access/download;Manuscript;biotoolsSchema paper - SUBMISSION (GigaScience).docx

biotoolsSchema : a formalised schema for bioinformatics software description

running head: biotoolsSchema : bioinformatics software schema

Jon Ison[1],* [1], Hans Ienasescu[1], [2], Emil Rydza [3], Piotr Chmura [3], Kristoffer Rapacki [4], Alban Gaignard [5], Veit Schwämmle [6], Jacques van Helden [7], Matúš Kalaš [8], Hervé Ménager [9]

(*) to whom correspondence should be addressed

[1] CNRS, UMS 3601, Institut Français de Bioinformatique, IFB-core, 2 rue Gaston Crémieux, F-91000, Evry, France

[2] National Life Science Supercomputing Center, Technical University of Denmark, Building 208, DK-2800 Kongens Lyngby, Denmark

[3] Novo Nordisk Foundation Center for Protein Research, Faculty of Health and Medical Sciences, University of Copenhagen, Blegdamsvej 3B, 2200 København, Denmark

[4] Department of Health Technology, Ørsteds Plads, Building 345C, DK-2800 Kongens, Lyngby, Denmark

[5] L'institut du Thorax, INSERM, CNRS, University of Nantes, 44007, Nantes, France

[6] Department of Biochemistry and Molecular Biology and VILLUM Center for Bioanalytical Sciences, University of Southern Denmark, Campusvej 55, 5230 Odense, Denmark

[7] Département de Biologie, Aix-Marseille Université (AMU), 3 place Victor Hugo - 13003 Marseille, France

[8] Computational Biology Unit, Department of Informatics, University of Bergen, N-5008 Bergen, Norway

[9] Hub de Bioinformatique et Biostatistique – C3BI, Institut Pasteur, USR 3756, CNRS, Paris (75015), France

[1] These authors contributed equally to this work.

Abstract

Life scientists routinely face massive and heterogeneous data analysis tasks, and must find and access the most suitable databases or software in a jungle of web-accessible resources. The diversity of information used to describe life science digital resources presents an obstacle to their utilisation. Although several standardisation efforts are emerging, no information schema has been sufficiently detailed to enable uniform semantic and syntactic description - and cataloguing - of bioinformatics resources. Here we describe biotoolsSchema, a formalised information model which balances the needs of conciseness for rapid adoption, while still providing rich technical information and scientific context. biotoolsSchema results from a series of community-driven workshops, and is deployed in the *bio.tools* registry, providing the scientific community with more 17,000 machine-readable and human-understandable descriptions of software and other digital life-science resources. We compare our approach to related initiatives and provide syntactic and semantic alignments to foster interoperability and reusability. The work supports the production of FAIR scientific software; a key element of open and reproducible developments for data-intensive sciences.

Availability and implementation: https://github.com/bio-tools/biotoolsschema
Contact: jon.c.ison@gmail.com
Supplementary Information: http://biotoolsschema.readthedocs.io/

Background

Workers in the life sciences must routinely describe, organise, find, understand, compare, select, use and connect a large and diverse set of analytical tools and data resources. These tasks can benefit greatly from detailed and consistent resource descriptions which are, when available, human-readable and, ideally, machine-readable. Consider for example the following tasks:

T1: A scientist surveying recently published tools in a general scientific area or for a specific computational task, highlighting those which are freely accessible.

T2: A bioinformatician constructing a data analysis pipeline, and searching for tool alternatives which perform a given operation on a specific type of biological data available in a particular format.

T3: A web developer tasked with building a portal to catalogue and promote the software outputs of a scientific community or consortium.

T4: A project manager assessing the software contributions including scientific impact of a particular project, institution, individual or research grant.

T5: A software developer wishing to contribute to open-source software projects, or seeking to claim credit for and promote their own contributions and productions.

These tasks can be challenging due to a lack of community-agreed standards or best practices to describe life science software and data resources. Even if open source software developers document their code for better (re)usability, the provided information may address very different aspects, with very different granularity levels. For instance, T1 would require the tool publication date, as well as its usage license, to be available and machine-readable. In practice, a common strategy is to manually search and browse a large variety of web pages, ranging from software-oriented resources (*e.g.* GitHub) to scientific literature resources (*e.g.* PubMed), sometimes through specific form-based search engines. Survey tasks are time consuming and often require repeated, and sometimes complex searches. As for T2, searching for tool alternatives is also challenging. In the best cases, software developers/providers precisely describe their contributions. But it is often difficult to compare two tools for a similar data analysis task because of the heterogeneity of their description. This would require a tool catalogue (T3) allowing for instance to filter tools based on their application domain, or the type of the data processing they provide. Other issues arise when claiming credit for software contributions (T5) or more generally evaluating scientific impact (T4). Citation recommendations are often provided as a paragraph in a tool's documentation, or using the structured Citation File Format - CFF [1]. Automated retrieval of such citation recommendations would be particularly useful in the context of virtual research environments where life scientists combine bioinformatics tools into data-intensive workflows.

All of these tasks depend highly on the availability of a shared human-understandable and machine-processable controlled vocabulary and syntax to precisely describe bioinformatics software and data resources. We thus propose biotoolsSchema. Our objective is twofold: (1) provide a technical means to formalize and express rich bioinformatics resource metadata required to achieve at least tasks T1-5, and (2) provide incentives for bioinformatics resource providers to enrich their tool metadata for better human/machine accessibility, readability and reusability. biotoolsSchema is a formalised information model that puts the description of a broad range of bioinformatics resources on a rigorous and consistent syntactic and semantic basis. Our model is developed through a community effort and has evolved steadily since its origin in the BioMedBridges [2] project (concluding in 2015), and more recently during its development for ELIXIR [3], resulting in the latest stable version 3.3.0. In *Comparison to related efforts* we introduce and compare biotoolsSchema to various relevant software metadata initiatives, in context of providing stable solutions to maintain FAIR principles [4] between software providers and consumers.

biotoolsSchema is broadly applicable, but optimised to describe bioinformatics *tools* - application software with well-defined data processing functions (inputs, outputs and operations). This includes simple tools with one or a few closely related functions, and complex, multimodal tools with many functions, available for immediate use as online services, or in a form which users can download, install, configure and run themselves.

biotoolsSchema defines 50 scientific, technical and administrative attributes. It concentrates upon salient common features, necessary and sufficient for the systematic cataloguing and use of tool information in a variety of contexts. It is available as XML Schema (XSD) and JSON Schema variants, and can be used to validate corresponding tool descriptions in XML, JSON and YAML formats. We summarise the design, methods and implementation of biotoolsSchema, comparing it to complementary approaches. We also summarise its applications, including the description of a dataset of over 17,000 tools registered in the *bio.tools* [5] registry (https://bio.tools).

Findings

Software attributes

biotoolsSchema covers a total of 50 scientific, technical or administrative software attributes, organised for convenience into 9 logical groupings (Figure 1, Table 1). To support the broadest range of applications, only bare-bones metadata (name, short description and homepage) are mandated, the rest of the attributes (Table 2) being conditionally required or optional. Element cardinality constraints (1 only, 1 to many, 0 or 1, 0 to many) were chosen to provide flexibility, where applicable. To enable concise information, standard identifiers are used where possible, *e.g.* DOIs for publications, ORCIDs [6] for people, ontology concept IDs for specialised scientific aspects, and controlled vocabularies for other attributes (see *Controlled vocabularies*). Verbose information, for example, software documentation, terms of use or citation instructions, are referred to by URL. Regular expression patterns are defined on all applicable elements to support precise syntax validation.

Figure 1. biotoolsSchema overview

Software attributes are organised into 9 groups (in boxes), and include terms from controlled vocabularies defined internally within biotoolsSchema, standard identifiers (including from the EDAM ontology), links or free text. Cardinality of the groups and attributes is shown in superscript and in the block arrows.

Table 1. Software attribute groups

Group	XML element	Description
Summary	–	Basic information about the software.

Labels	–	Miscellaneous scientific, technical and administrative details of the software, expressed in terms from controlled vocabularies.
Functions	`function`	Details of the function(s) (*i.e.* modes of operation) the software provides, expressed in concepts from the EDAM ontology.
Links	`link`	Miscellaneous links for the software *e.g.* repository, issue tracker or mailing list.
Downloads	`download`	Links to downloads for the software, *e.g.* source code, virtual machine image or container.
Documentation	`documentation`	Links to documentation about the software *e.g.* user manual, API documentation or training material.
Relationships	`relation`	Details of a relationship this software shares with other software registered in *bio.tools*.
Publications	`publication`	Publications about the software.
Credits	`credit`	Individuals or organisations that should be credited, or may be contacted about the software.

Software attributes are grouped within biotoolsSchema. The groups correspond to XML elements with the exception of 'Summary' and Labels' group.

Table 2. Software attributes

<<< SEE END OF DOCUMENT >>>

biotoolsSchema covers 50 general software attributes grouped for convenience. EDAM concepts may be specified by one or both of an URI or term. "enum" indicates a controlled vocabulary defined by biotoolsSchema. Attributes of type xs:token or URL include where applicable regular expressions for syntax validation.

Scientific concepts

The EDAM ontology [7] provides the core vocabulary for the scientific description of tools including types of data and data identifiers, data formats, operations and topics. EDAM organises these concepts into the EDAM Topic, Operation, Data and Format sub-ontologies. Concepts may be specified by one or both of an EDAM concept URI (*e.g.* http://edamontology.org/topic_0121) and/or a term (*e.g.* "Proteomics") - a preferred label or synonym of a concept from the appropriate EDAM sub-ontology. It is strongly recommended to specify at least the URI, as these persistently identify a concept (labels and synonyms can change).

Model of tool function

The model of tool functionality (Figure 2) is concise and simple. It supports a practical summary of a tool's essential functionality including primary inputs and outputs from the perspective of a typical biologist end-user. Each software entity may have one or more functions, each corresponding to a mode of operation that the software provides. In turn, each function performs one or more basic operations, and has zero or more primary input and/or output data. Each input or output is of a specified data type and has supported format(s). Operation (*e.g.* "Sequence alignment"), data type (*e.g.* "Sequences") and format (*e.g.* "FASTA") are EDAM concepts. An optional comment, and relevant command, command-line fragment or option for executing the function, may also be specified.

Figure 2. Model of tool function

biotoolsSchema follows a simple model of tool function, where each function (mode of operation) performs one or more specific operations. Each operation may have one or more primary inputs and outputs, each of a defined data type and listing supported format(s). Illustration is for the ProCon (biotools:procon) conversion utility.

Auxiliary information

Miscellaneous links, downloads and documentation are modelled in a common way (Figure 3) including a URL, type, and an optional comment. Specifying the types of documentation *etc.* via controlled vocabularies allows these to be extended in the future, in a way that is non-breaking to schema dependencies.

Figure 3. Model of links, downloads and documentation

Links, downloads and documentation are modelled in a common way; a URL which is annotated to indicate facets (such as issue tracker, code repository etc.) and an optional comment. Additionally, downloads also allow for associated version information. Illustration is for miscellaneous links.

Publications

Publications of relevance to software must be specified by (at least) one of a DOI (Digital Object Identifier), PMID (PubMed reference number) or PMCID (PubMed Central reference number), and may be optionally typed, *e.g.* "Review". Use of DOIs - the most generic of these identifiers - is recommended.

Tool relationships

Relationships between tools that have been registered in *bio.tools* may be specified by biotoolsID and a term from a controlled vocabulary which is currently limited to *isNewVersion*/*hasNewVersion* (version relationships), *uses*/*usedBy* (general functional association) and *includes*/*includedIn* (primarily for associating collections such as software suites with their constituent tools). These relationship types will be extended in due course.

Credits and contact information

Credits and contacts for a tool are handled by a consolidated mechanism. Creditable or contactable entities of various types ("Person", "Institute" *etc.*) and roles ("Developer", "Support" *etc.*) must have at least one or more of a name, email and URL. Open Researcher and Contributor IDs (ORCID ID) provide a persistent reference to information on an individual person. Global Research Identifier Database Identifiers (GRID IDs) and Research Organization Registry Identifiers (ROR IDs) are used for organisations, and Crossref Funder Registry Identifiers (FundRef /Funder IDs) for funding organisations. Specification of these IDs, where available, is strongly recommended as these enable sustainable maintenance and reuse of relevant metadata.

Controlled vocabularies

In addition to EDAM, a further 16 controlled vocabularies (Table 3) catering for technical aspects are defined internally within biotoolsSchema as *standardised enumerations of terms*. Notably the license controlled vocabulary uses identifiers from the industry standard SPDX list [8]. Comprehensive documentation (see *Documentation*) including definitions of term in each vocabulary is available online (*https://biotoolsschema.readthedocs.io/en/latest/controlled_vocabularies.html*) and is embedded in the XSD schema file.

Table 3. Controlled vocabularies

**Controlled vocabulary (#terms)**	**Description**
identifier type (4)	The type of tool identifier, *e.g.* "doi"
tool type (15)	The type of application software, *e.g.* "Command-line tool"
operating system (3)	The operating system supported by a downloadable software package, *e.g.* "Linux"
programming language (57)	Name of programming language the software source code was written in, *e.g.* "C"
license (326)	Software or data usage license, *e.g.* "GPL-3.0"
maturity (3)	How mature the software product is, *e.g.* "Mature"
cost (3)	Monetary cost of acquiring the software, *e.g.* "Free of charge"
accessibility (3)	Whether there are non-monetary restrictions on accessing an online service, *e.g.* "Open access"
elixirPlatform (5)	ELIXIR research infrastructure technical platform, *e.g.* "Tools"
elixirNode (22)	ELIXIR research infrastructure national node, *e.g.* "France"
elixirCommunity (11)	Name of relevant ELIXIR (or other) community, *e.g.* "Galaxy"
link type (12)	The type of data, information or system that is obtained when the link is resolved, *e.g.* "Helpdesk"
download type (18)	Type of download that is linked to, *e.g.* "Source code"
documentation type (15)	Type of documentation that is linked to, *e.g.* "API documentation"
publication type (6)	Type of publication, *e.g.* "Review"
relation type (6)	Type of tool relationship, *e.g.* "uses"
credit entity type (6)	Types of entities that may be credited, *e.g.* "Person"
credit entity role (7)	Roles that may be assigned to creditable entities, *e.g.* "Developer"

biotoolsSchema defines 16 controlled vocabularies catering for technical aspects of software description.

Implementation of biotoolsSchema in *bio.tools*

bio.tools (https://bio.tools) provides the means - manually via graphical user interfaces and programmatically via a REST API - for a user to browse and search over biotoolsSchema-formatted data, and to add to, edit or download the registry content. Tool description data registered or downloaded via the REST API in a choice of serialisation formats (XML, JSON or YAML) are compatible with biotoolsSchema. *bio.tools* unique tool identifiers (*e.g.* "signalp", biotools:signalp) are used in persistent *bio.tools* URLs (*e.g.* https://bio.tools/signalp) resolving to Tool Cards summarising essential tool information. The *bio.tools* compact URIs (*e.g.* "biotools:signalp") are a convenient short form; simply the identifier in the "biotools" namespace. biotoolsSchema supports other types of ID, and software version information may be attached to the entire tool description, or to a specific ID, download or publication in a flexible way, allowing for example a single version label, or a list or range of labels to be annotated. In case a single label annotation reflects a rigorous assignment of software version made by the tool developer, this can be used in conjunction with the *bio.tools* tool ID to uniquely identify a particular software artefact.

As of June 2020, *bio.tools* includes 17,263 entries and a total of 300,216 individual annotations, including 100,854 terms from the EDAM ontology, as per attributes defined within biotoolsSchema. Individual tool descriptions vary in richness, and are being progressively improved, through an initiative [9] that engages the community with the curation process [10] ; for example producing a high-quality tools description corpus for proteomics data analysis [11]. The *bio.tools* content, user interfaces and API will be described in more details in a future publication.

Serialisation formats and transformations

bio.tools supports upload and download of biotoolsSchema-formatted data in a choice of serialisation formats (XML, JSON or YAML). XML support in *bio.tools* was developed using XSLT transformations to support two-way, lossless interconversions between biotoolsSchema-formatted XML files and the JSON, YAML and generic XML formats that are natively supported by the Django web framework used by *bio.tools*. This offers maximum flexibility to providers and consumers of biotoolsSchema-formatted data, allowing for rigorous validation (against the XSD) irrespective of favoured format. The transforms are freely available from https://github.com/bio-tools/biotoolsShim/. For illustration purposes, a sample JSON file for the SignalP command-line tool (biotools:signalp) (Figure 4) is shown. As a future work, other serialisation formats will be supported, including JSON-LD to support Linked Data applications.

Figure 4. Sample JSON file for signalp tool


```
{
	"name": "SignalP",
```

"description": "Prediction of the presence and location of signal peptide cleavage sites in amino acid sequences from different organisms.",
"homepage": "http://cbs.dtu.dk/services/SignalP/",
"biotoolsID": "signalp",
"biotoolsCURIE": "biotools:signalp",
"version":
[
"4.1"
],
"otherID": [
{
"value": "rrid:SCR_015644",
"type": "rrid"
}
],
"toolType":
[
"Command-line tool",
"Web application"
],
"topic":
[
{
"uri": "http://edamontology.org/topic_3510",
"term": "Protein sites, features and motifs"
}
],
"operatingSystem":
[
"Linux",
"Mac"
],
"license": "Other",
"collectionID":
[
"CBS"
],
"maturity": "Mature",
"cost": "Free of charge (with restrictions)",
"function":
[
{
"operation":
[
{
"uri": "http://edamontology.org/operation_0418",
"term": "Protein signal peptide detection"
},
{
"uri": "http://edamontology.org/operation_0422",
"term": "Protein cleavage site prediction"
}
],
"input":
[
{

```
                              "data":
                              {
                                    "uri": "http://edamontology.org/data_2044",
                                    "term": "Sequence"
                              },
                              "format": [
                                    {
                                          "uri":
"http://edamontology.org/format_1929",
                                          "term": "FASTA"
                                    }
                              ]
                        }
                  ],
                  "output":
                  [
                        {
                              "data": {
                                    "uri": "http://edamontology.org/data_1277",
                                    "term": "Protein features"
                              },
                              "format":
                              [
                                    {
                                          "uri":
"http://edamontology.org/format_2305",
                                          "term": "GFF"
                                    }
                              ]
                        },
                        {
                              "data":
                              {
                                    "uri": "http://edamontology.org/data_2955",
                                    "term": "Sequence report"
                              }
                        }
                  ],
                  "note": "predicts the presence and location of signal peptide
cleavage sites in amino acid sequences from different organisms"
            }
      ],
      "link":
      [
            {
                  "url": "http://www.cbs.dtu.dk/cgi-bin/sw_request?signalp",
                  "type":
                  [
                        "Repository"
                  ]
            }
      ],
      "download":
      [
            {
                  "url": "http://www.cbs.dtu.dk/cgi-bin/sw_request?signalp",
```

```
            "type": "Source code",
            "note": null,
            "version": null
        },
        {
            "url": "http://www.cbs.dtu.dk/cgi-bin/sw_request?signalp",
            "type": "Binaries",
            "note": null,
            "version": null
        }
    ],
    "documentation":
    [
        {
            "url": "http://www.cbs.dtu.dk/services/SignalP",
            "type":
            [
                "General"
            ]
        }
    ],
    "publication":
    [
        {
            "doi": "10.1038/nmeth.1701",
            "pmid": "21959131",
            "type":
            [
                "Primary"
            ]
        }
    ],
    "credit":
    [
        {
            "name": "TN Petersen",
            "typeEntity": "Person",
            "typeRole":
            [
                "Developer"
            ]
        },
        {
            "name": "CBS",
            "typeEntity": "Institute",
            "typeRole":
            [
                "Provider"
            ]
        },
        {
            "name": "Henrik Nielsen",
            "email": "hnielsen@cbs.dtu.dk",
            "orcidid": "http://orcid.org/0000-0002-9412-9643",
            "typeRole":
            [
```

```
                        "Developer"
                  ]
            },
            {
                  "name": "Henrik Nielsen",
                  "email": "hnielsen@cbs.dtu.dk",
                  "orcidid": "http://orcid.org/0000-0002-9412-9643",
                  "typeEntity": "Person",
                  "typeRole":
                  [
                        "Primary contact"
                  ]
            }
      ]
}
```


Comparison to related efforts

Various research infrastructure or community-led initiatives (Table 4) have defined, or are in the process of defining, sets of information fields to describe bioinformatics software application metadata. The *HCLS Community Profile* [12] was an early effort of the Semantic Web Health Care and Life Sciences Interest Group [13]. It specifies dataset descriptions using the Resource Description Framework (RDF), using 24 core metadata elements and recommends re-use of various well established, general-purpose RDF controlled vocabularies including Dublin Core [14], Friend-of-a-Friend [15] and PROV [16]. In contrast, the Citation File Format, CFF [1] is a YAML format for general-purpose software annotations. Tangential to these is the *Schema.org vocabulary*, founded by the major web search engine providers. It is an exhaustive controlled vocabulary for things on the internet, organised into a hierarchy of a very broad range of conceptual classes. It includes concepts relevant to software such as *SoftwareApplication* and *CreativeWork,* and is well suited for general purpose, lightweight mark-up of web pages for discovery purposes. A *Tool Profile* under development for the Bioschemas project [17] will provide guidelines on the consistent adoption of schema.org markup for the description of software tools in the life sciences, including for example recommending the use of EDAM ontology for scientific aspects.

Parallel to initiatives depending upon *Semantic Web* technologies, are efforts reflecting existing practice or the requirements of various research infrastructures. The *CodeMeta Metadata Crosswalk* produced by the CodeMeta community project [18] is a table reflecting a comparison of software metadata used across multiple code repositories and systems. The crosswalk (a work in progress) yields an exhaustive set of 65 software metadata concepts (mostly mapped to schema.org concepts), and can inform efforts to produce a more minimal concept vocabulary for software, reflecting a consensus in the mapping. The *DataCite Metadata Schema* from DataCite [19] - a non-profit organisation

that provides persistent identifiers (DOIs) for research data - includes core metadata properties primarily for resource identification, citation and retrieval, encapsulated is an XML schema with usage guidelines. The *Application Profile* included in the *Guidelines for Software Repository Managers* from OpenAIRE [20] - a European project supporting Open Science - is based on DataCite and covers 23 software attributes, primarily to make software products citable. Similarly, the *Software Citation Principles* produced by Force 11 [21] community initiative, defines 11 basic metadata requirements for software citation. The *eInfraCentral Service Description Template* produced by the European E-Infrastructure Services Gateway, eInfraCentral [22], is (as a work in progress) defining the information requirement for a common E-Infrastructures service catalogue, that will describe and offer services to end-users in a harmonised way, through the European Open Science Cloud (EOSC) portal [23].

Table 4. Software metadata initiatives

Initiative	Description
HCLS Community Profile	Specification for dataset description using RDF. https://www.w3.org/TR/hcls-dataset/
eInfraCentral Service Description Template	Information model for European eInfrastructure services (including software services). https://jnp.gitbooks.io/service-description-template-v1-12/basic_service_information/
DataCite Metadata Schema	XML schema and guidelines of core metadata properties for resource identification, citation and retrieval. https://schema.datacite.org/
OpenAIRE Application Profile	Guidelines for software repository managers. https://software-guidelines.readthedocs.io/en/latest/application_profile.html
CodeMeta Metadata Crosswalk	Vocabulary for software metadata concepts and crosswalk between software metadata projects. https://github.com/codemeta/codemeta/blob/master/crosswalk.csv
Schema.org Vocabulary	Controlled vocabulary for marking up web pages. https://schema.org
Bioschemas Tool Profile	Schema.org specification for tools in the Life Sciences. http://bioschemas.org/devSpecs/Tool/
Force 11 Software Citation Principles	Basic metadata requirements for software citation. https://www.force11.org/software-citation-principles

Various initiatives for software medata of relevance to biotoolsSchema are shown.

This summary of initiatives is not exhaustive. Others include the DOE CODE initiative [24] for code archiving by the U.S. Department of Energy (DOE), and guidelines [25] for rich search results for software from Google, and specialised

ontologies for software including SWO [26] and OntoSoft [27], each serving a different use-case. We have thus a plethora of different recommendations and ways to annotate and share software metadata. The diversity reflects a wide range of perspectives, use-cases and contexts, but brings the challenge of curating software metadata and sharing it between systems, whilst avoiding inconsistencies and duplication of efforts. We ameliorate this interoperability issue, at least so far as sharing and re-using *bio.tools* metadata, through an exhaustive crosswalk (Table 5) between biotoolsSchema elements and key software metadata initiatives including CodeMeta, schema.org, OpenAire, DataCite, HCLS, eInfraCentral, Force11 and miscellaneous RDF vocabularies. Each element in biotoolsSchema was mapped, where possible, to the corresponding field used by these initiatives, and the mappings aggregated, discussed and reviewed, resulting in a consolidated crosswalk (Table 5) for biotoolsSchema, that has been submitted to the CodeMeta. The crosswalk thus provides a framework useful to any engineer integrating software metadata provided in these contexts.

Table 5. Comparison of biotoolsSchema and other software metadata initiatives
<<< SEE END OF DOCUMENT >>>
Elements in biotoolsSchema are mapped to equivalent elements from various software metadata initiatives. Only those elements which could be mapped are shown.

Discussion

The efficiency of workers utilising scientific software across the spectrum of the Life Sciences depends, in a large part, upon high quality and convenient bioinformatics software metadata. biotoolsSchema, in combination with EDAM, provides a formalised, rigorous and consistent specification of the syntax and semantics for these metadata. This enables software developers and service providers to define their productions in a consistent way, cataloguers to communicate clearly what is available, and software end-users to more efficiently use these resources. biotoolsSchema thus encompasses diverse use-cases, from provenance through to query and discovery, and can help to standardize the curation and exchange of metadata across software projects, repositories, initiatives and organizations.

In biotoolsSchema, a great complexity of information - including tool functionality, fields of use, interfaces, deployments, distributions, documentation and so on - is reduced to a manageable and practical level. The model is applicable to nearly all technical types of tool, and supports the uniform description of key scientific, technical and administrative attributes. Specifically, it allows for a presentation and comparison of tool information, which often cannot conveniently be obtained from a Google search or cursory inspection of a provider's website. With progressive development, biotoolsSchema applications such as *bio.tools* will help to make complex tool functions more easily understood, and render tools more accessible, usable and interoperable, *i.e.* more FAIR [4]. For example, a recent study

[28] demonstrated the usefulness of biotoolsSchema-formatted data for automated workflow composition in mass spectrometry-based proteomics data analysis.

Our standalone schema allows for community development of the model to be loosely coupled to applications such as *bio.tools*, and provides a means for an end-user to validate content external to any system, ensuring correct syntax, structure and completeness. biotoolsSchema must evolve to keep pace with developments in the field and support new applications and integration scenarios. This may include richer modelling of the complex relationships between resources, and support for specialised biological ontologies such as Gene Ontology [29] for molecular function, cellular component and biological process, Sequence Ontology [30] for genomic elements, and NCBI Taxonomy [31] for taxa. biotoolsSchema will thus provide a means to relate a large set of tools such as in *bio.tools* to a broader and flourishing ecosystem of workflows, databases and ontologies.

Future changes will be pragmatic, driven by community use-cases, and in light of practical experience of what data is useful and readily available. No single model, registry or initiative can hope to cover all bases. biotoolsSchema can be augmented by (and will not duplicate) the functionalities of related well-maintained models provided by more specialised initiatives, *e.g.* execution-layer information about command-line tools provided by CWL [32], or information about service endpoints supported by OpenAPI [33]. Volatile attributes, or attributes which must be frequently recalculated, such as metrics of usage, technical performance data, links to similar tools, software dependencies, hardware requirements, and so on, will remain out of scope.

Different software metadata use-cases have different information requirements. biotoolsSchema supports very minimal or much more comprehensive information specifications, according to needs, without imposing a high curation burden and thus a barrier to adoption. It provides the basis for, but cannot in itself specify, a flexible information requirement suited to diverse purposes and contexts, *e.g.* curation of registries such as *bio.tools*, required information for service delivery plans, publication of software articles, or metrics for software metadata or project quality. For such purposes, a framework [34] for tool information requirements is under development, which is based on biotoolsSchema, but goes beyond the syntactic / semantic constraints that can conveniently be defined in XML schema. Human-readable guidelines for curation of software metadata are also being developed as part of an emerging Curators Guide [35].

biotoolsSchema, with progressive development and adoption, can benefit the whole bioinformatics community. It can help to support best practices promoted by various research infrastructures which emphasize the value of bioinformatics software registries for findability [36, 37], and bridge the gap between technology-oriented developers and service-oriented research infrastructures and organisations. The field of software metadata management is socially and technically very complex, and includes many more stakeholders and perspectives than are summarised here, with

multiple projects serving different but overlapping needs, use-cases, and contexts. We encourage all such efforts and warmly welcome collaborations for the continued development of biotoolsSchema, its applications and integration into the broader bioinformatics ecosystem.

Methods

Design considerations

Requirements were established during a series of community-led workshops resulting in ten founding principles and design considerations, now implemented as characteristics of biotoolsSchema:

- ***Practical*** - focus on salient attributes of practical value in everyday use; especially to support the discovery, use and practical interoperability of software; superfluous details are excluded.
- ***General*** - generally applicable, *i.e.* to all manner of bioinformatics resources (see *Scope*).
- ***Consistent*** - use ontologies and standardised enumerations of terms (see *Controlled Vocabularies*) where possible, to support precise searches over biotoolsSchema-formatted data and return of consistent and therefore comparable information.
- ***Concise*** - mandate URLs or standard identifiers where possible, helping to ensure the sustainable upkeep of biotoolsSchema-formatted data and support future integrations, applications and cross-linking with other resources.
- ***Simple*** - biotoolsSchema is as flat (unstructured) as is practicable, ensuring ease of use, whilst preserving essential structure, *e.g.* a meaningful model of tool function.
- ***Compatible*** - it is inevitable that tool providers, integrators, and cataloguers will continue to use a variety of models, methods and formats for tool descriptions; biotoolsSchema is broadly compatible (see *Comparison to related efforts*) to support future integration scenarios.
- ***Extensible*** - to cater for emerging requirements, and adaptable by others for their own purposes.
- ***Stable*** - the maintenance of software dependencies on mutating schema is expensive. Backwards incompatible changes are only made if absolutely required (see *Development process and status*).
- ***Free and open source*** - to encourage reuse and new applications.
- ***Community-driven development*** - to ensure end-user needs are satisfied.

Scope

biotoolsSchema is applicable to a nearly complete range of application software, including command-line tools, scripts, libraries, workflows, web applications, database portals, web APIs, web services, SPARQL endpoints, desktop applications, plug-ins, workbenches and suites. These tool types and their definitions were settled following an analysis of *bio.tools* and are included as a controlled vocabulary within biotoolsSchema (see *Controlled vocabularies*). They are intended to provide a practical and intuitive designation. In principle, when describing a tool, one or more tool types may be assigned, reflecting the different facets of the software being described. biotoolsSchema includes general attributes such as tool description, publication and license. Execution-layer information, for example command-line tool options or Web service endpoints, are for now at least out of scope. biotoolsSchema thus complements for example Galaxy [38] or Common Workflow Language (CWL) command line tool descriptions, and OpenAPI descriptions of web services.

Development process and status

The model is an evolution of an early prototype developed for BioMedBridges, that began in 2012. It has undergone many iterations of intensive community critique and development, primarily during ELIXIR workshops. In parallel, development was informed by growth in *bio.tools*: major content providers and other end-users have helped to validate the model, with the registry itself providing a valuable dataset for this purpose. Thus, we consider the stable version (3.3.0) to satisfy major community requirements, and provide a solid foundation upon which the content, functions, integration and applications of portals such as *bio.tools* can be built. The model must be subject to future improvements, but major changes are restricted to approximately yearly in released stable versions, to provide stability for developers and software dependencies. Future versions will not depart fundamentally from the attributes or structure described in this article. From version 3.0.0, version numbers follow the SemVer 2.0.0 scheme (https://semver.org/). Developments can be followed at GitHub (https://github.com/bio-tools/biotoolsschema).

Documentation

The schema is comprehensively and consistently documented:

- textual (human-readable) description of each schema element
- additional, highly concise element descriptions, suitable for example as tips in user interfaces
- definition of terms in all controlled vocabularies
- mapping of schema elements including controlled vocabularies to other relevant models and vocabularies

- usage information including technical details (such as syntax and use of *bio.tools* API) and curation guidelines (good practice on using biotoolsSchema to describe tools)
- information about the project and community

The documentation is, where possible, encoded within the XSD and JSON schemas, but also made available online in a more user-friendly form:

- https://biotoolsschema.readthedocs.io/ (project docs)
- http://bio-tools.github.io/biotoolsSchema/ (technical docs)
- https://bio-tools.github.io/biotoolsSchemaJ/ (technical docs, JSON schema variant)
- https://biotools.readthedocs.io/en/latest/api_usage_guide.html (technical usage guidelines)
- http://biotools.readthedocs.io/en/latest/curators_guide.html (curator guidelines)

Availability and requirements

biotoolsSchema is licensed under a Creative Commons Attribution-ShareAlike 4.0 International License (CC BY-SA 4.0):

- https://github.com/bio-tools/biotoolsschema

The *bio.tools* content is freely available to all under the Creative Commons Attribution licence (CC BY 4.0) and can be downloaded from *bio.tools*:

- https://bio.tools

Funding

This work was supported by funding from the Danish Ministry of Higher Education and Science (ELIXIR Denmark) and from the European Union's Horizon 2020 research and innovation programme (grant agreement no 676559, ELIXIR-EXCELERATE).

Acknowledgements

Jon Ison warmly acknowledges the support of the Institut Français de Bioinformatique. Thanks to Dmitry Repchevsky, Wojtek Dabrowsk, and other attendees of ELIXIR workshops for their suggestions.

Author contributions

JI developed the schema and wrote the article with contributions from all authors. HI, ER and PC implemented the schema in *bio.tools*.

Competing interests

The authors declare no competing interests.

References

[1] Stephan D, Morane G, Neil CH, Raniere S, Bast, Radovan; Michael RC. Citation File Format - CFF. https://zenodo.org/record/1117789#.XtVWBhbRbJU (2017).

[2] Suhr S. et al. REPORT: BioMedBridges workshop on e-Infrastructure support for the life sciences – Preparing for the data deluge. Preprint at http://doi.org/10.5281/zenodo.13942 (2015).

[3] Crosswell LC & Thornton JM. ELIXIR: a distributed infrastructure for European biological data. *Trends in Biotechnology*, **30**(5), 241–242 (2012).

[4] Wilkinson MD et al. The FAIR Guiding Principles for scientific data management and stewardship. *Scientific Data*, **3**, 160018 (2016).

[5] Ison J. et al. The bio.tools registry of software tools and data resources for the life sciences. Genome Biology **20**, 164 (2019)

[6] ORCiD website. https://orcid.org/. Accessed 6 June 2020.

[7] Ison J. et al. EDAM: an ontology of bioinformatics operations, types of data and identifiers, topics and formats. *Bioinformatics*, **29**(10), 1325–1332 (2013).

[8] Lovejoy J, Odence P, & Lamons S. Advancing the Software Package Data Exchange: An update on SPDX. *International Free and Open Source Software Law Review*, **5**(2), 145–152 (2013).

[9] Ison J. et al. Tools and data services registry: a community effort to document bioinformatics resources. *Nucleic Acids Research*, **44**(D1), D38–D47 (2016).

[10] Ison J. et al. Community curation of bioinformatics software and data resources. *Briefings in Bioinformatics*, **bbz075** (2019)

[11] Tsiamis V., Ienasescu H., Gabrielaitis D, Palmblad M, Schwämmle V. & Ison J. One Thousand and One Software for Proteomics: Tales of the Toolmakers of Science. *Journal of Proteome Research,* **18** (10), 3580-3585 (2019)

[12] Gray AJG et al. The HCLS Community Profile. https://www.w3.org/TR/hcls-dataset. Accessed 6 June 2020.

[13] Semantic Web Health Care and Life Sciences Interest Group. https://www.w3.org/2011/09/HCLSIGCharter. Accessed 6 June 2020.

[14] Dublin Core Metadata Initiative. http://dublincore.org/documents/2003/02/12/dcmi-type-vocabulary. Accessed 6 June 2020.

[15] Friend-of-a-Friend Vocabulary Specification. http://xmlns.com/foaf/spec. Accessed 6 June 2020.

[16] Missier P, Belhajjame K, & Cheney J. The W3C PROV family of specifications for modelling provenance metadata. In *Proceedings of the 16th International Conference on Extending Database Technology - EDBT '13* (2013)

[17] Michel F & The Bioschemas Community. Bioschemas & Schema.org: a Lightweight Semantic Layer for Life Sciences Websites. *Biodiversity Information Science and Standards*, **2:e25836** (2018).

[18] The CodeMeta project. https://codemeta.github.io. Accessed 6 June 2020.

[19] DataCite website. https://www.datacite.org. Accessed 6 June 2020.

[20] OpenAIRE website. https://www.openaire.eu/. Accessed 6 June 2020.

[21] Smith AM, Katz DS, Niemeyer KE, & FORCE11 Software Citation Working Group. Software Citation Principles. *PeerJ Computer Science,* **2:e86 (**2016).

[22] European E-Infrastructure Services Gateway, eInfraCentral. http://einfracentral.eu/. Accessed 6 June 2020.
[23] European Open Science Cloud (EOSC) portal. https://eosc-portal.eu/. Accessed 6 June 2020.
[24] DOE CODE initiative. https://www.osti.gov/doecode/. Accessed 6 June 2020.
[25] Google schema.org guidelines for software apps. https://developers.google.com/search/docs/data-types/software-app. Accessed 6 June 2020.
[26] Malone J. et al. The Software Ontology (SWO): a resource for reproducibility in biomedical data analysis, curation and digital preservation. *Journal of Biomedical Semantics,* **5**, 25 (2014).
[27] Yolanda G, Ratnakar V. and Garijo D. OntoSoft: Capturing Scientific Software Metadata. *Proceedings of the Eighth ACM International Conference on Knowledge Capture (K-CAP)*, Palisades, NY (2015).
[28] Palmblad M, Lamprecht AL, Ison J and Schwämmle V. Automated workflow composition in mass spectrometry-based proteomics. *Bioinformatics,* **35**(4):656-664 (2019).
[29] Ashburner M. et al. Gene ontology: tool for the unification of biology. The Gene Ontology Consortium. *Nature Genetics*, **25**(1), 25–29 (2000).
[30] Eilbeck K et al. The Sequence Ontology: a tool for the unification of genome annotations. *Genome Biology* **6** (R44) (2005).
[31] Federhen S. The NCBI Taxonomy database. *Nucleic Acids Research*, **40** (Database issue), D136–D143 (2012).
[32] Amstutz P. et al: Common Workflow Language, v1.0. Specification, *Common Workflow Language working group*. Preprint at https://doi.org10.6084/m9.figshare.3115156.v2 (2016)
[33] OpenAPI initiative. https://www.openapis.org. Accessed 6 June 2020.
[34] Tool Information Profiles. https://github.com/bio-tools/tool-Information-profiles. Accessed 6 June 2020.
[35] *bio.tools* Curators Guide. http://biotools.readthedocs.io/en/latest/curators_guide.html. Accessed 6 June 2020.
[36] Jiménez RC et al. Four simple recommendations to encourage best practices in research software. *F1000Research*, **6** (2017).
[37] Jagodnik KM et al. Developing a framework for digital objects in the Big Data to Knowledge (BD2K) commons: Report from the Commons Framework Pilots workshop. *Journal of Biomedical Informatics*, **71**, 49–57 (2017).
[38] Afgan E et al. The Galaxy platform for accessible, reproducible and collaborative biomedical analyses: 2016 update. *Nucleic Acids Research*, **44**(W1), W3–W10 (2016).

Table 2. Software attributes

**XML element/ JSON property**	**Description**	**Type**	**Cardinality**
(Summary group)			
`name`	Canonical software name assigned by the software developer or service provider.	string	1 only
`description`	Textual description of the software.	string	1 only
`homepage`	Homepage of the software, or some URL that best serves this purpose.	URL	1 only
`biotoolsID`	Unique ID (case insensitive) of the tool that is assigned upon registration of the software in *bio.tools*, normally identical to tool name.	bio.tools tool ID	0 or 1
`biotoolsCURIE`	*bio.tools* CURIE (compact URI) based on the *bio.tools* tool ID.	URI	0 or 1
`version`	Version information (typically a version number) of the software applicable to this *bio.tools* entry.	string	0 or more
`otherID`	*A unique identifier of the software, typically assigned by an ID-assignment authority other than bio.tools.*		*0 or more*
`otherID->value`	Value of tool identifier.	string	1 only
`otherID->type`	Type of tool identifier.	enum	0 or 1

`otherID->version`	Version information (typically a version number) of the software applicable to this identifier.	string	0 or 1
(Labels group)			
`toolType`	A type of application software: a discrete software entity can have more than one type.	enum	0 or more
`topic`	General scientific domain the software serves or other general category.	EDAM Topic	0 or more
`operatingSystem`	The operating system supported by a downloadable software.	enum	0 or more
`language`	Name of programming language, *e.g.* used for the software source code or compatible with an API.	enum	0 or more
`license`	Software or data usage license.	enum	0 or 1
`collectionID`	Tag for a collection that the software has been assigned to within *bio.tools*.	string	0 or more
`maturity`	How mature the software product is.	enum	0 or 1
`cost`	Monetary cost of acquiring the software.	enum	0 or 1
`accessibility`	Whether there are non-monetary restrictions on accessing an online service.	enum	0 or 1
`elixirPlatform`	ELIXIR platform credited for developing or providing the software.	enum	0 or more
`elixirNode`	ELIXIR node credited for developing or providing the software.	enum	0 or more
`elixirCommunity`	Name of relevant ELIXIR (or associated) community.	enum	0 or more
`function` *(0 or more)*			
`operation`	The basic operation(s) performed by this software function.	EDAM Operation	1 or more
`input|output`	*Details of primary input / output.*		*0 or more*
`input|output ->data`	Type of primary input or output data.	EDAM Data	1 only
`input|output ->format`	Allowed format(s) of the input or output data (EDAM Format).	EDAM Format	0 or more
`note`	Concise comment about this function, if not apparent from the software description and EDAM annotations.	string	0 or 1
`cmd`	Relevant command, command-line fragment or option for executing this function / running the tool in this mode.	string	0 or 1
`link` *(0 or more)*			
`url`	A link of some relevance to the software.	URL	1 only
`type`	The type of data, information or system that is obtained when the link is resolved.	enum	1 or more
`note`	Comment about the link.	string	0 or 1
`download` *(0 or more)*			
`url`	Link to download (or repository providing a download) for the software.	URL	1 only

type	The type of data, information or system that is obtained when the link is resolved.	enum	1 only
note	Comment about the download.	string	0 or 1
version	Version information (typically a version number) of the software applicable to this download.	string	0 or 1
documentation *(0 or more)*			
url	Link to documentation on the web for the tool.	URL	1 only
type	Type of documentation that is linked to.	enum	1 or more
note	Comment about the documentation.	string	0 or 1
relation *(0 or more)*			
biotoolsID	*bio.tools* ID of an existing bio.tools entry to which this software is related.	bio.tools tool ID	1 only
type	Type of relation between this and another registered software.	enum	1 only
publication *(0 or more)*			
doi*	Digital Object Identifier of a publication about the software (* at least one of doi, pmid or pmcid must be specified).	doi	0 or 1*
pmid*	PubMed Identifier.	pmid	0 or 1*
pmcid*	PubMed Central Identifier.	pmcid	0 or 1*
type	Type of publication.	enum	0 or more
version	Software version information (typically number) applicable to this publication.	string	0 or 1
note	Comment about the publication.	string	0 or 1
credit *(0 or more)*			
name*	Name of the entity that is credited (* at least one of name, email or url must be specified).	string	0 or 1*
email*	Email address.	email address	0 or 1*
url*	URL, e.g. homepage of an institute.	URL	0 or 1*
orcidid	Unique identifier (ORCID iD) of an entity that is credited.	ORCID iD	0 or 1
gridid	Unique identifier (GRID ID) of an organisation that is credited.	GRID ID	0 or 1
rorid	Unique identifier (ROR ID) of an organisation that is credited.	ROR ID	0 or 1
fundrefid	Unique identifier (FundRef ID or Funder ID) of a funding organisation that is credited.	FundRef ID	0 or 1
typeEntity	Type of entity that is credited.	enum	0 or 1
typeRole	Role performed by the entity that is credited.	enum	0 or more
note	A comment about the credit.	string	0 or 1

Table 5. Comparison of biotoolsSchema and other software metadata initiatives

bio.tools	**CodeMeta / schema.org**	**OpenAire 18/23**	**DataCite**	**Misc. RDF vocabs**	**HCLS**	**eInfraCentral**	**Force11 10/11**

	36/65[2]		11/19[3]		12/24[4]	23/24[5]	
summary							
name	name	Name	Title	rdfs:label, dct:title	Title	Service Name	Software name
description	description	Description	Description	rdfs:comment, dct:description	Description	Service Tagline, Service Description	Description
homepage	url	Landing page (datacite:alternateIdentifier)	-	foaf:page	HTML page	Service URL, Service Order	-
biotoolsID	identifier	Identifier	Identifier	dct:identifier		Service ID	Unique identifier
biotoolsCURIE	identifier	Identifier	Identifier	dct:identifier		(Service ID)	
version	softwareVersion	Version	Version	pav:version pav:hasCurrentVersion pav:previousVersion	Version identifier	Service Version	Version number
otherID	identifier	Alternate identifier	AlternateIdentifier, alternateIdentifierType	rdfs:seeAlso, dct:identifier	-	-	-
function							
operation	-	-	-	dcat:keyword	-	Service Tags	-
input|output ->data	-	-	-	dcat:keyword	-	-	-
input|output ->format	-	-	-	dcat:keyword	-	-	-
note	-	-	-	rdfs:comment	-	-	-
cmd	-	-	-	rdfs:comment	-	-	-
labels							
toolType	applicationSubCategory	Software Type	ResourceType	rdfs:comment		(Service Category, Service Subcategory)	
topic	keywords	Subject	Subject, valueURI	dcat:keywords	Keywords	Service Tags, Service Coverage	Keywords
operatingSystem	operatingSytem	-	-	dct:medium dct:mediator	-	-	-
language	programmingLanguage	Programming Language (datacite:format)	-	dct:language	Language	-	-
license	license	License Condition	Rights, rightsURI	dct:licence	License	-	Software license
collectionID	-	-	-	dct:identifier	-	-	-
maturity	-	-	-	-	-	Service Life Cycle	-

[2] 55 of which are schema.org properties and 10 of which are specific to Codemeta
[3] disregarding DataCite subproperties
[4] HCLS core metadata elements
[5] disregarding service level targets and performance information (out of biotoolsSchema scope)

						Status[6]	
`cost`	isAccessibleForFree[7]	-	-	-	-	Service Cost	-
link							
`any type`	relatedLink	-	-	-	-	-	-
`"Repository"`	codeRepository	Repository (datacite:publisher)	-	-	-	-	Location / repository
`"Helpdesk"`	-	-	-	-	-	Service Helpdesk, Service Feedback	-
`"Mailing list"`	-	-	-	-	-	Service Feedback	-
`"Issue tracker"`	codemeta:issueTracker	-	-	-	-	-	-
download							
`any type`	downloadUrl	-	-	dcat:downloadURL, prov:atLocation	-	-	-
`"Source code", "Software package" or "Binaries"`	-	Distribution location (datacite:alternateIdentifier)	-	dcat:mediaType	-	-	-
`"Icon"`	-	-	-	-	Logo	Service Symbol	-
`"Screenshot"`	-	-	-	-	-	Service Multimedia	-
documentation							
`"General"`	codemeta:readme	Documentation (datacite:alternateIdentifier)	-	dcat:landingPage	Documentation	-	-
`"User manual"`	softwareHelp	-	-	-	-	Service User Manual	-
`"Terms of use"`	-	Access Rights (datacite:rights)	Rights, rightsURI	dct:rights	Rights	Service Terms of Use	-
`"Training material"`	-	-	-	-	-	Service Training Information	-
Publication							
`doi|pmid|pmcid`	referencePublication	-	-	dct:references	References	-	Index citations
credit							
`name`	givenName, familyName, affiliation, name	-	creatorName, givenName, familyName, affiliation,	foaf:name	-	[8], [9]	-
`email`	email	-	-	foaf:mbox	-	-	-
`url`	url	-	-	foaf:page prov:atLocation	-	-	-
`orcidid`	identifier	-	nameIdentifier	dct:identifier	-	-	-

[6] eInfraCentral uses a different but compatible controlled vocabulary
[7] True where cost=="Free of charge"
[8] with `typeEntity`=="Institute" and `typeRole`=="Provider"
[9] where `typeEntity`=="Funding agency"

`typeEntity`	-	-	nameType	-	-	-	-
`typeRole`	-	-	-	foaf:providedBy pav:createdBy pav:authoredBy pav:curatedBy pav:contributed By	-	-	-
`typeEntity=="Funding agency"`	funder, codemetada:funding	Funding Reference	funderName, FundingReference	-	-	Service Funding	-
`typeRole=="Developer"`	author, creator	Author	Creator	dct:creator	Creators	-	Author(s)
`typeRole=="Contributor"`	contributor, editor	-	Contributor	-	-	-	Contributor role
`typeRole=="Provider"`	provider, producer	-	-	-	-	Service Provider Name	-
`typeRole=="Maintainer"`	codemeta:maintainer	-	-	-	-	-	-
`typeRole=="Primary contact"`	-	Contact Person , Contact Group	-	dct:contributor	Contributors	-	-

(1) These fields are compatible with EDAM vocabulary.

Figure 3 Click here to access/download;Figure;Figure3.png

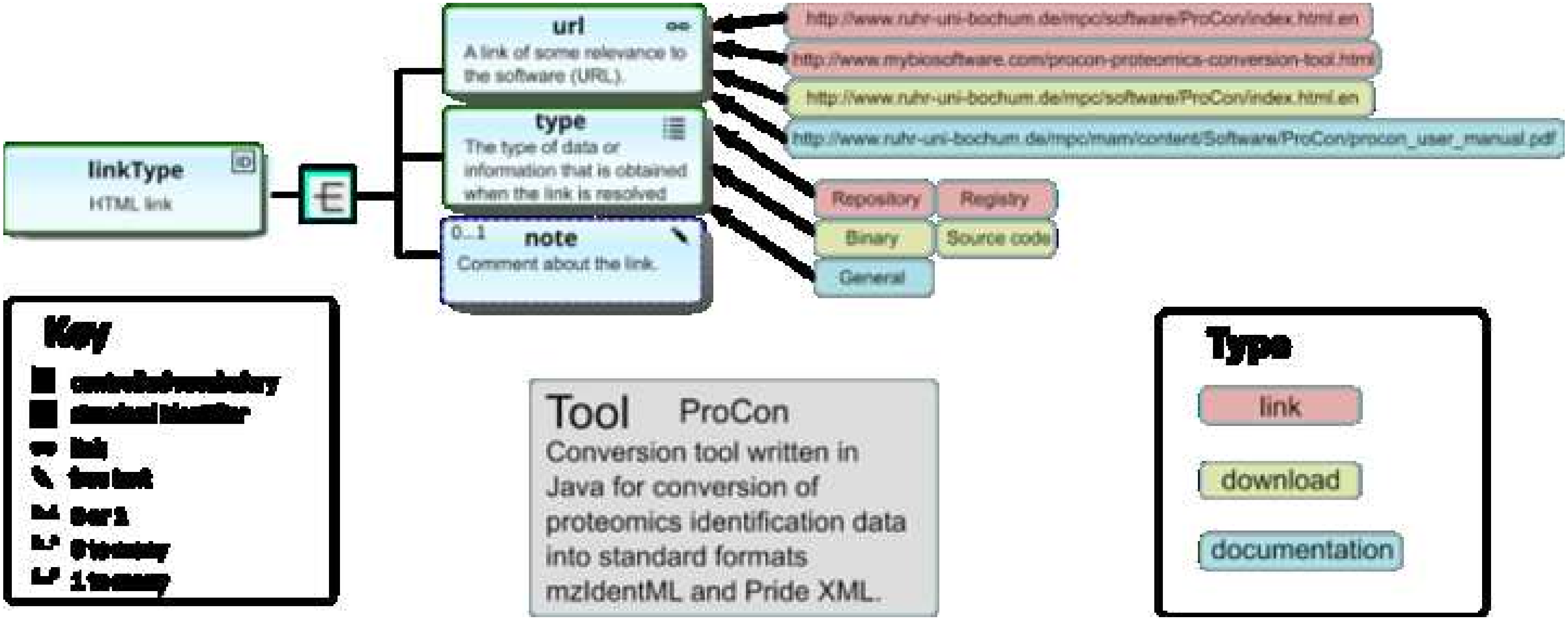

Figure 2

Click here to access/download;Figure;Figure2.png

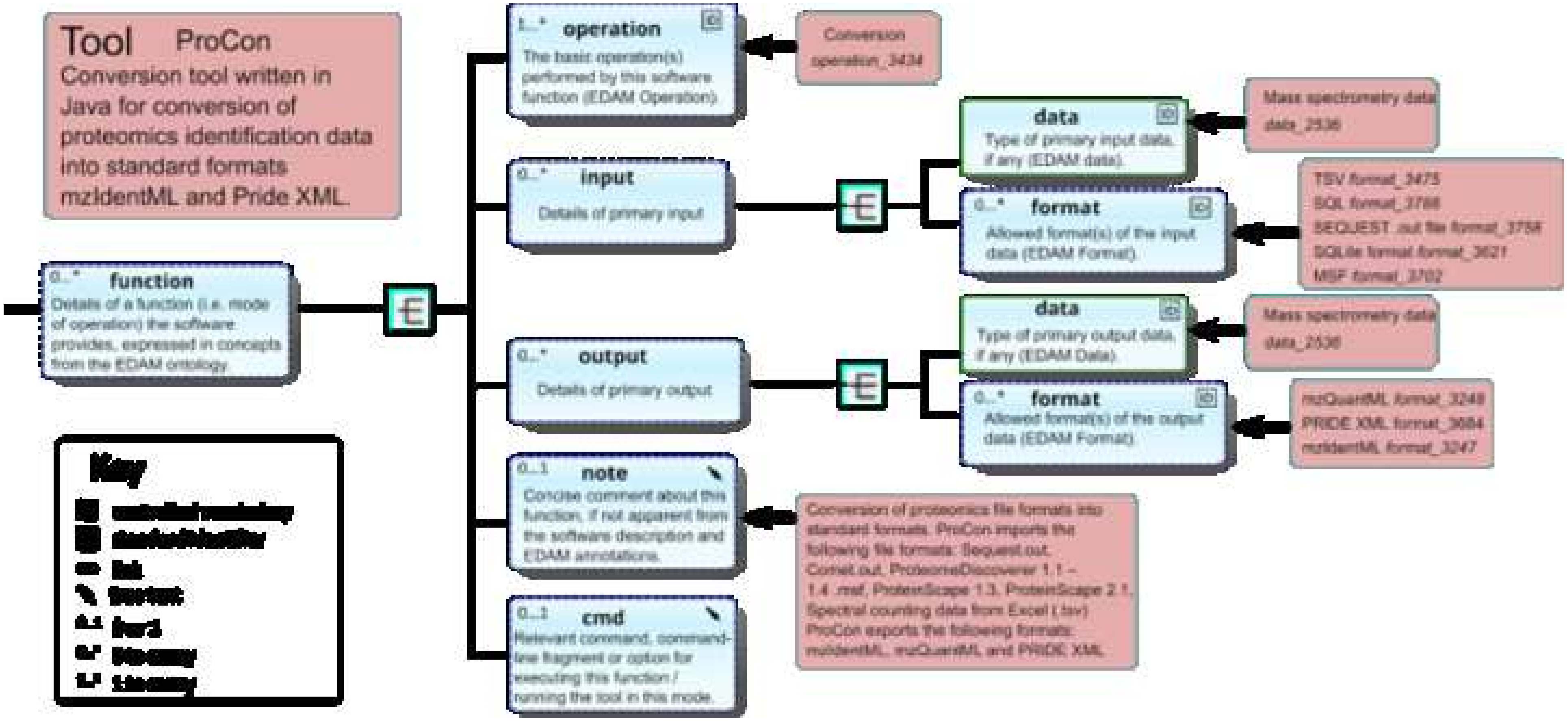

Figure 1

Click here to access/download;Figure;Figure1.png

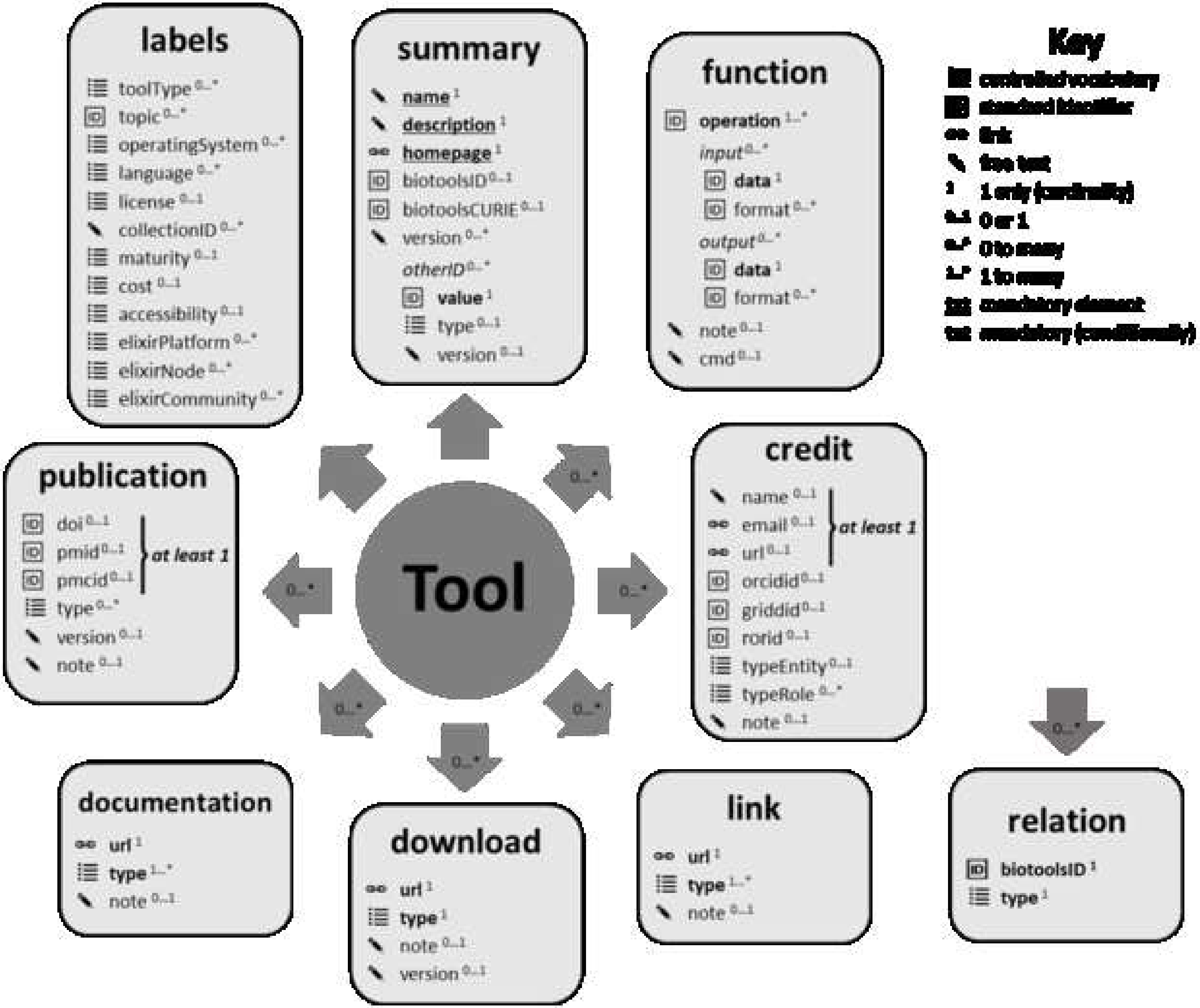

Cover letter Click here to access/download;Personal Cover;Cover letter Ison et al.pdf

RE: Submission of manuscript "biotoolsSchema : a formalised schema for bioinformatics software description" for consideration of publication in *GigaScience*

Submission Date: July 6th, 2020

Dear GigaScience Editors,

The use of software is ubiquitous in the biological and biomedical sciences, but the field has lacked a uniform way for developers to describe their tools and help researchers understand, compare and utilise all the diverse software that is available. Our manuscript summarises the results of an effort - spanning 8 years - in producing a comprehensive, general-purpose schema (biotoolsSchema) that enables a broad range of tools to be described in a concise, precise and consistent way. biotoolsSchema provides both a syntax and (by leveraging the EDAM ontology) the semantics for life science research software. It is used in (and has co-evolved with) the *bio.tools* registry for the description of 17K+ tools provided by 2.4K contributors. We hope that by publishing a technical description of the schema in GigaScience, we can bring the work to the attention of a bigger audience - to find new applications beyond *bio.tools*, to spare costly reinvention of the functions provided by biotoolsSchema, and more broadly, to promote the provision of high quality software metadata in general - to benefit the whole life sciences globally.

We would like to highlight that biotoolsSchema is fully compatible with, but serves quite a different purpose to the generic *schema.org*. The latter provides a set of fields for mark-up of web pages (see for example https://developers.google.com/search/docs/data-types/software-app), primarily to support "rich results" in web browsers such as Google. In contrast, biotoolsSchema provides a syntax (including regular expressions) and semantics (EDAM + 18 further controlled vocabularies) for over 50 key scientific, technical and administrative attributes. It is thus suited both as an exchange format and for applications in software discovery and interoperability. For example, the schema (or parts of it) can be used for software descriptions in repositories such as GitHub, and re-used downstream in various settings. To put biotoolsSchema in context and to help developers, we include in the article a technical comparison to related initiatives, including a metadata crosswalk that has been submitted to CodeMeta.

On behalf of the authors, I declare there are no competing interests, that all authors have approved the manuscript for submission, and that the content of the manuscript has not been published, or submitted for publication elsewhere. I was not certain which of the GigaScience article types is most appropriate - probably *Technical Note* - so I have prepared the text and sections in a manner which I think best conveys the narrative of the article.

I would like to suggest the following reviewers, who the authors do not have close ties with, but I think would give the article a fair hearing:

Brian O'Connor (boconnor@broadinstitute.org)
Egon Willighagen (egon.willighagen@gmail.com)
Tim Griffin (tgriffin@umn.edu)

With best regards

Jon Ison

jon.c.ison@gmail.com
