# Supplementary material for: biotoolsSchema: a formalized schema for bioinformatics software description
Source: Gigascience. 2021 Jan 27;10(1):giaa157. doi: 10.1093/gigascience/giaa157 (PMC7842104; doi:10.1093/gigascience/giaa157)
Supplement: giaa157_GIGA-D-20-00206_Revision_1 [file giaa157_giga-d-20-00206_revision_1.pdf]

GigaScience

biotoolsSchema : a formalised schema for bioinformatics software description

--Manuscript Draft--

**Manuscript Number:**	GIGA-D-20-00206R1
**Full Title:**	biotoolsSchema : a formalised schema for bioinformatics software description
**Article Type:**	Technical Note
**Funding Information:**	Danish Ministry of Higher Education and Science — Mr Piotr Chmura<br>H2020 European Research Council (676559) — Not applicable<br>Institut Français de Bioinformatique (IFB / ELIXIR France) — Dr Jon Ison
**Abstract:**	Background<br><br>Life scientists routinely face massive and heterogeneous data analysis tasks, and must find and access the most suitable databases or software in a jungle of web-accessible resources. The diversity of information used to describe life-scientific digital resources presents an obstacle to their utilisation. Although several standardisation efforts are emerging, no information schema has been sufficiently detailed to enable uniform semantic and syntactic description - and cataloguing - of bioinformatics resources.<br><br>Findings<br><br>Here we describe biotoolsSchema, a formalised information model which balances the needs of conciseness for rapid adoption, while still providing rich technical information and scientific context. biotoolsSchema results from a series of community-driven workshops, and is deployed in the bio.tools registry, providing the scientific community with more than 17,000 machine-readable and human-understandable descriptions of software and other digital life-science resources. We compare our approach to related initiatives and provide alignments to foster interoperability and reusability.<br><br>Conclusions<br><br>biotoolsSchema supports the formalised, rigorous and consistent specification of the syntax and semantics of bioinformatics resources, and enables cataloguing efforts such as bio.tools that help scientists to find, comprehend and compare resources. The use of biotoolsSchema in bio.tools promotes the FAIRness of research software; a key element of open and reproducible developments for data-intensive sciences.<br><br>Availability and implementation<br><br>https://github.com/bio-tools/biotoolsschema
**Corresponding Author:**	Jon Ison, PhD<br>French Institute of Bioinformatics (IFB-Core)<br>Évry, FRANCE
**Corresponding Author Secondary Information:**	
**Corresponding Author's Institution:**	French Institute of Bioinformatics (IFB-Core)
**Corresponding Author's Secondary Institution:**	
**First Author:**	Jon Ison, PhD
**First Author Secondary Information:**	
**Order of Authors:**	Jon Ison, PhD<br>Hans Ienasescu

Powered by Editorial Manager® and ProduXion Manager® from Aries Systems Corporation

	Emil Rydza
	Piotr Chmura
	Kristoffer Rapacki
	Alban Gaignard
	Veit Schwämmle
	Jacques van Helden
	Matúš Kalaš
	Hervé Ménager
**Order of Authors Secondary Information:**	
**Response to Reviewers:**	### Please see file "biotoolsSchema paper _ response to reviewers.docx" attached during submission. Included verbatim beow.<br><br>Dear Dr Zauner,<br><br>On behalf of the authors I would like to thank you, and the reviewers for their very thorough treatment of our manuscript. They raise many relevant points, and we address each of these in detail in our point-by-point response, indicating changes made to the manuscript. We have also structured the abstract into sections and included ORCIDs for authors for whom these are available.<br><br>As for the key points you highlight - evidence that biotoolsSchema supports FAIR principles, and issues around immutability, persistence and the minimal mandatory core of metadata, we address these rigorously in our responses (points 1.8, 1.9, 1.10 and 1.17 below). Immutability and persistence of software metadata is delivered by bio.tools, through its ID scheme and Tool Cards (point 1.10, Table 6, plus revised text in the section "Implementation of biotoolsSchema in bio.tools"). The justification for the minimal mandatory core of metadata - which is a practical necessity for building a large-scale registry such as bio.tools based on biotoolsSchema, is explained in points 1.8 and 1.9 (with revisions to the text in “Software attributes”). We have included in the manuscript a new Table 6 which summarises how bio.tools and biotoolsSchema support each of the FAIR principles (points 1.10 and 1.17) as enumerated in https://www.force11.org/group/fairgroup/fairprinciples, and subsequently mapped to the software space in https://content.iospress.com/articles/data-science/ds190026. We intend (in a future work) to go further, and produce a set of objective and transparent metrics of FAIRness, based on biotoolsSchema attributes, and calculate these for all bio.tools entries using a new Tool Information Profile system (https://github.com/bio-tools/Tool-Information-Profiles) that is being developed for this purpose.<br><br>With best regards<br><br>Jon<br><br>Dr Jon Ison<br>jon.c.ison@gmail.com<br><br>Response to reviewer #1<br><br>1.1 “What is the justification for 50 attributes?”<br>The 50 attributes in the schema are simply what we ended up with after many iterations of development and releases over the years of biotoolsSchema development - a major driver of this effort being community workshops and (crucially) engaging with and incorporating the requirements of bio.tools content providers and end-users, with respect to what information people find valuable and are prepared to provide.<br><br>1.2 “It is unclear if or how this list is extensible over time. If this standard can evolve, why state 50, and if it cannot, what assurances are there that it will continue to be an effective descriptive schema in the future?”

The list of attributes certainly is extensible over time and biotoolsSchema was designed openly, in a community-based process that was described in https://academic.oup.com/bib/article/21/5/1697/5560007 (current version is 3.3.0), and is licensed for this purpose; reuse and contributions are welcome. New official releases will incorporate end-user requirements, changes can be requested through collaboration with the authors, GitHub issues etc.
Version 3.3.0 is the ninth in a series of public releases, and in each of them the attributes list was revised, to reflect the needs of our users. This latest list of 50 attributes is not cast in stone, but rather is the current status of the schema, and it might (and will probably) evolve in the future as new requirements emerge. We have added a sentence to the section "Development process and status" clarifying the above.

1.3 "There are instances where this list already seems restrictive, such as "accessibility" in Table 3 which appears to support any of three options, though it is easy to imagine many more distinct types of access control, for instance."
Indeed, and should a compelling use-case arise, the "accessibility" options can easily be extended (all such controlled vocabularies are defined as simple enumerations of terms in the schema). In practice, one has to strike a balance between what attributes reasonably capture salient details, and what is realistic to curate and useful to end-users. The rigorous semantics and syntax of biotoolsSchema has advantages over approaches such as the use of folksonomies, which are very flexible, but can be less tractable in the context of registries such as bio.tools. Where possible, biotoolsSchema re-uses well established controlled vocabularies which are maintained independently, such as SPDX for software license.

1.4 "The relationship between CWL or other execution standards and biotoolsSchema is presently unclear. Can a CWL definition be included/referenced within a biotoolsSchema?"
biotoolsSchema is a metadata format that provides a description of software tools and services to address its findability, but not its execution, whereas CWL Tools, Galaxy Tools, and other execution formats allow the execution of tools in workflow environments but do not enable an exhaustive description of the resources. Acknowledging this difference, biotoolsSchema allows adding links to execution formats (see for instance the link to the CWL wrappers from the yara bio.tools entry https://bio.tools/yara), and reversely some links to bio.tools entries can be added to CWL wrappers (see for instance https://github.com/common-workflow-library/bio-cwl-tools/blob/release/qualimap/qualimap_rnaseq.cwl#L21) and Galaxy wrappers (see for instance https://github.com/galaxyproject/tools-iuc/blob/master/tools/circos/circos.xml#L5).

1.5 "Similarly, what is the line between an execution standard and what is included in biotoolsSchema? For instance, it appears as though inputs and outputs are defined here, which is a considerable portion of the execution standard. This apparent duplication of content between tool descriptions gives rise to the possibility of inconsistency between them. If an execution record is referenced, are there validators which could be used to ensure consistency of duplicated information across both records?"
The relationship and degree of overlap between registry-focused formats such as biotoolsSchema have been explored in previous work (cite "Using registries to integrate bioinformatics tools and services into workbench environments", doi:10.1007/s10009-015-0392-z), and used to help the generation of execution formats (cite "Using bio. tools to generate and annotate workbench tool descriptions", doi:10.12688/f1000research.12974.1). Future developments based on these works will probably be focused on improving such tooling to resolve inconsistencies between such formats and cross-validating the different descriptions. We expanded slightly the text of the "Discussion" section to cite and summarise this work.

1.6 "Does this schema/manuscript propose a mechanism for storage or access of these records aside from the bio.tools website?"
The biotoolsSchema format itself is not, in its essence, restricted to usage within bio.tools. One of the current efforts led by the ELIXIR Europe organization is the creation of a github-based platform to store and exchange openly software tool metadata between multiple resources within ELIXIR (e.g. bio.tools tools registry,

Powered by Editorial Manager® and ProduXion Manager® from Aries Systems Corporation

BioContainers containers registry, OpenEBench benchmarking and monitoring platform, usegalaxy.eu portal) and beyond it (BioConda, Debian Med, etc.). This platform, by allowing the different resources to push their data and pull other data, will facilitate the cross-linking of their records and cross-consolidation of their metadata. Eventually, we aim at allowing the maintenance of tool metadata as biotoolsSchema files in their source repositories which will be automatically synchronized with this platform. We plan to publish a description of this emerging platform once it is more mature.

1.7 "What assurance that this website and service will persist beyond a funding cycle, for instance? (i.e. Is it supported by a large public group organization? Could it rely upon such a service, e.g. Zenodo?). If this is not addressed, the records would not live up to the FAIR requirement of persistency and immutability."
bio.tools is supported by ELIXIR Europe, and is one of the commissioned services (https://elixir-europe.org/about-us/commissioned-services/registry-tools) of this organization. As such, not only do bio.tools and biotoolsSchema involve the work of multiple national groups (e.g., in Denmark, France and Norway), but they are funded and evaluated with the specific goals of ensuring their long term availability and sustainability. Reviewer 2 also raised a comment about the sustainability; we have added a short paragraph (in “Development process and status”) to describe how biotoolsSchema development is (through its anchoring within the ELIXIR infrastructure) sustainable.

1.8 “Please discuss the justification for making such a large majority of the fields optional. If the intent is to truly have richly described and queryable tools, the bar for flexibility appears to currently be set too low for this goal to be reached, also limiting the strength of the claim that the metadata is "high quality".”
The core of mandatory attributes is indeed intentionally small, having been whittled down during the evolution of the project, and was found to be a necessity for the curation of tools as such large scale. The primary reason was to encourage (by settling an easily achievable goal) new contributions, and also to facilitate contributions from institutes, projects etc. who wanted to deposit a large number of tools with basic descriptions in a first pass, and then subsequently improve those descriptions. Even a basic entry goes a long way to making a tool more FAIR, for reasons now summarised in Table 6. A secondary reason is that biotoolsSchema has a very broad scope in terms of the types of resources that it can be used to describe; not all attributes are applicable to all types of tool, furthermore, not all attributes are available from all contributors. The fact there are many very rich descriptions (see e.g. http://proteomics.bio.tools/) is evidence that a low bar for the number of mandatory attributes certainly does not, in itself, preclude high quality. Internally, we do track information richness using our “Tool Information Standards” system, which describes what attributes should be provided at various tiers of detail and quality. This system is summarised in https://academic.oup.com/bib/article/21/5/1697/5560007.

1.9 “The current flexibility may have serious consequences on the consumption of described tools, such as in the case where licensing information is not provided or known by consumers. While the schema supports the FAIR curation of tools when well implemented, the usefulness of this schema is severely limited if the minimum specification does not.”
We are well aware that the availability of data can be a serious issue. For exactly the reason pointed out by the reviewer, we have been developing the “Tool Information Standards” system (described in https://academic.oup.com/bib/article/21/5/1697/5560007) used internally in bio.tools into a more flexible, robust and independent service. Progress on this is available at https://github.com/bio-tools/Tool-Information-Profiles. Tool Information Profiles will, in due course, replace the current “Tool Information Standards” system. In short, a tool information profile specifies which tool attributes (defined in biotoolsSchema) must, should or may be specified for different types of tools within a set of tool descriptions. It thus augments (and ameliorates the limitations of) the small mandatory core attributes defined by biotoolsSchema, by allowing to adapt these requirements to project or community-specific requirements. A practical application will be to use such profiles for filtering, or targeted improvement of sets of tool descriptions, before consumption by other systems. We are hoping to publish this work in due course.

Powered by Editorial Manager® and ProduXion Manager® from Aries Systems Corporation

	1.10 "It would be valuable to query existing descriptions in bio.tools and see what portion of them meet the standard of being FAIR. This analysis could be included as a sample use-case showcasing the value of biotoolsSchema, as well, and provide further justification and clarification around its adoption."<br>We do agree on the value of evaluating FAIRness of software tools, however, in practice this is non-trivial owing to the numerous and complex indicators and metrics corresponding to FAIRness, that have been subject to much debate. To make a pragmatic start, we examined the criteria defined in “Towards FAIR principles for research software" (https://content.iospress.com/articles/data-science/ds190026), with respect to the features of bio.tools and biotoolsSchema, and specifically their impact on the FAIRness of a tool. The results of this comparison have been added to the manuscript as Table 6.<br>We would like, and intend to go further, but this has to be done with great care, given the obvious sensitivities of the implied assignation of some tools as FAIR, and some not, and especially because we would be evaluating FAIRness on the metadata we have in bio.tools (tool authors should have the possibility to improve their entries before we evaluate them). We envisage developing a Tool Information Profile (as previously mentioned) for FAIRness, and use it to provide an open, transparent and flexible framework to evaluate FAIRness of all tools in bio.tools, using biotoolsSchema data. This requires community agreement on an exact set of metrics (which should be objective and transparent) for its evaluation. While the indicators in “Towards FAIR principles for research software" are an excellent starting point, these are by no means the only set of metrics. We will therefore, in due course, run a community event to explore these metrics and advance this work. We hope that this is, for now, an adequate response.<br><br>1.11 “Much of the Comparison to related efforts section reads more like a list than flowing text. Please add supporting text to make this read more naturally, and situate biotoolsSchema explicitly relative to these efforts, emphasizing novel elements.”<br>We have revised this section extensively along the lines suggested. It would be possible to write an entire article which compares and contrasts the various approaches, historical and contemporary, which exist in this space, so we hope our revision will suffice. We include in the revision various new relevant developments around bio.tools and biotoolSchema.<br><br>1.12 “The mention of tasks at the beginning of the manuscript is not mentioned or referenced later on once the schema has been presented. The efficacy for this schema at accomplishing each task should be explicitly mentioned as its features or attributes are introduced and discussed.”<br>This is a good point, and an omission on our side. We have revised the Discussion accordingly, to refer back to the tasks mentioned in the Introduction.<br><br>1.13 “The explicit comparison of features or interfaces between tools is an excellent feature, and I think it should be more prominently mentioned.”<br>We added a sentence in the "Background" section that emphasizes this feature (provision of a model for the description of tool functions), and also modified the text in the Discussion to mention that advanced possibilities such as workflow composition or provenance tracking are mostly enabled by this original feature.<br><br>1.14 “Another description standard (specifically, an execution standard for tools much like CWL) which closely aligns itself with enabling the FAIR principles is Boutiques (https://boutiques.github.io); consider referencing this standard, and in particular, the tooling it provides to facilitate fair curation of records (more details here: https://figshare.com/articles/poster/fair-pipelines-poster_pdf/8143241).”<br>We thank the reviewer for pointing us to this relevant work we were not aware of. We have indeed added it , and its contribution to software FAIRness, to the related work we refer to in the paper.<br><br>1.15 “The design considerations section provides a list that could be of extreme value to tool and standard developers. Could this be provided as an independent resource or checklist that is made more widely available?”<br>We thank the reviewer for his interest in this content. Following his suggestion, we added it to the public documentation of biotoolsSchema, it can be found at https://biotoolsschema.readthedocs.io/en/latest/design_considerations.html.

Powered by Editorial Manager® and ProduXion Manager® from Aries Systems Corporation

1.16 “The scope for biotoolsSchema is unclear for the majority of the manuscript, and should be placed closer to the beginning. In particular, the relationship or relative objectives with this and execution standards such as CWL or bioinformatics ontologies such as EDAM.”
We have moved the complete "Scope" subsection to the "Findings" section, where we believe it helps get a better overview of biotoolsSchema. A detailed comparison of the relationship and relative objectives of "registry-focused" (e.g. biotoolsSchema) and "execution-focused" (e.g. CWL) tool descriptions was published in a previous paper (https://link.springer.com/article/10.1007/s10009-015-0392-z), which we now cite in this article.

1.17 “FAIR terms should be added to the table which compares ontologies (in particular, the 15 as enumerated here: https://www.force11.org/group/fairgroup/fairprinciples), possibly in the Force11 column.”
The FAIR principles listed at this URL were mapped and analysed with respect to software in the article “Towards FAIR principles for research software" (https://content.iospress.com/articles/data-science/ds190026). We have in turn added to the article (in new Table 6) a summary of how bio.tools and biotoolsSchema supports each of these principles. The detailed mapping of indicators of FAIRness to biotoolsSchema attributes, with respect to producing a set of objective and transparent metrics of FAIRness will be the subject of a future work using the Tool Information Profile system, as outlined at length in a previous point (see point 1.10).

1.18 “Text in the legends, as well as parts of the figure itself, for figures 1, 2, and 3 is unreadable.”
We have provided larger / higher resolution versions of Figures 1, 2 and 3 which are more readable.

Response to reviewer #2

2.1 "All schemas benefit greatly from being community driven, and the authors do note that extensive community consultation has been undertaken to arrive at a community consensus as to the content of the schema, but provide few details of the mechanism that was employed that has led to the consensus. I would recommend that inclusion of this information is critical to illustrate that the schema is indeed community agreed, and a summary describing who, what and how the community consensus was reached would be beneficial (e.g. details of workshops, working group membership etc), as any governance type arrangements that have led to each version being agreed / 'signed off'"
We thank Reviewer 2 for his interest in this important aspect of our work. As mentioned in other places in this letter, the overall work of community development of biotoolsSchema is part of a wider process also involving the development of the bio.tools registry, the EDAM ontology and other components of the ELIXIR Tools Platform, as outlined in a recent publication (cite https://academic.oup.com/bib/article/21/5/1697/5560007). The schema development has been in context of major European infrastructure projects (BioMedBridges, ELIXIR EXCELERATE) and ELIXIR national node infrastructures and has leveraged their governance structures, e.g. ELIXIR EXCELERATE WP1 partners. The current governance structure can be seen at https://biotoolsschema.readthedocs.io/en/latest/contributors.html. Over the years biotoolsSchema development has featured at many hackathons, meetings and workshops, using agile methods (e.g. feature poker, sprints etc.) with open participation (within and beyond ELIXIR). It would be too verbose to describe in detail all of this (which are summarised at https://biotools.readthedocs.io/en/latest/events.html), so we have added a short summary on the governance, and how we arrived at a community consensus to the section "Development process and status" of the manuscript, and hope this will suffice.

2.2 “The authors state that "future changes will be pragmatic, driven by community-use cases". The paper would benefit from inclusion of clear guidelines or instructions on how the wider community can provide feedback which may influence the future

	development of the schema - ie. how to provide feedback on v3.3.0 and how to get involved in influencing any future versions."<br>The mechanisms of community engagement around biotoolsSchema (and other technologies in its orbit) have been mentioned in https://academic.oup.com/bib/article/21/5/1697/5560007. We have updated the text (in section "Development process and status") to provide a short summary and refer to the paper mentioned. We have also added contribution guidelines to the online docs (https://biotoolsschema.readthedocs.io/en/latest/what_is_biotoolsschema.html#how-to-contribute-to-biotoolsschema) and link to these from a new CONTRIBUTING.md file (hhttps://github.com/bio-tools/biotoolsSchema/blob/master/CONTRIBUTING.md) in biotoolsSchema repo.<br><br>2.3 "Some commentary on the potential sustainability of the schema would be useful - is its use recommended, mandated or required by any groups? The authors briefly discuss the involvement of the ELIXIR consortium in its development, and I note that three controlled vocabularies exist for ELIXIR platform, community and node, so I am guessing that its use is at least recommended by ELIXIR. Some clarification around the use of the schema in ELIXIR and any other other efforts would be valuable to help illustrate how widespread adoption is / is likely to be moving forward."<br>Reviewer 1 also raised a comment (see point 1.7) about the sustainability; we have added a short paragraph (in the section "Development process and status") to describe how biotoolsSchema development is (through it's anchoring within the ELIXIR infrastructure) sustainable including a note about its current and likely future adoption. In short, bio.tools is supported by ELIXIR Europe, and is one of the commissioned services (https://elixir-europe.org/about-us/commissioned-services/registry-tools) of this organization. As such, not only do bio.tools and biotoolsSchema involve the work of multiple national groups (e.g., in Denmark, France and Norway), but they are funded and evaluated with the specific goals of ensuring their long term availability and sustainability.<br><br>2.4 "Table 3 states that there are 16 controlled vocabularies, however 18 are listed and 18 are included in the online documentation https://biotoolsschema.readthedocs.io/en/latest/controlled_vocabularies.html."<br>This error has been corrected.<br><br>2.5 "Figures 2 and 3 are quite small / low resolution - these would benefit from being larger / higher resolution."<br>We have included larger versions of Figures 2 and 3.<br><br>2.6 "Similar to Figure 2, Figure 3 should also include a note that the illustration example is for the ProCon tool."<br>We have included the note as suggested.
**Additional Information:**	
**Question**	**Response**
Are you submitting this manuscript to a special series or article collection?	No
**Experimental design and statistics**<br><br>Full details of the experimental design and statistical methods used should be given in the Methods section, as detailed in our Minimum Standards Reporting Checklist. Information essential to interpreting the data presented should be made available in the figure legends.	Yes

Have you included all the information requested in your manuscript?	
**Resources**<br><br>A description of all resources used, including antibodies, cell lines, animals and software tools, with enough information to allow them to be uniquely identified, should be included in the Methods section. Authors are strongly encouraged to cite Research Resource Identifiers (RRIDs) for antibodies, model organisms and tools, where possible.<br><br>Have you included the information requested as detailed in our Minimum Standards Reporting Checklist?	Yes
**Availability of data and materials**<br><br>All datasets and code on which the conclusions of the paper rely must be either included in your submission or deposited in publicly available repositories (where available and ethically appropriate), referencing such data using a unique identifier in the references and in the “Availability of Data and Materials” section of your manuscript.<br><br>Have you have met the above requirement as detailed in our Minimum Standards Reporting Checklist?	Yes

Manuscript Click here to access/download;Manuscript;biotoolsSchema paper_for_production.docx

biotoolsSchema : a formalised schema for bioinformatics software description

running head: biotoolsSchema : bioinformatics software schema

Jon Ison[1],* [1], Hans Ienasescu[1], [2], Emil Rydza [3], Piotr Chmura [3], Kristoffer Rapacki [4], Alban Gaignard [5], Veit Schwämmle [6], Jacques van Helden [7], Matúš Kalaš [8], Hervé Ménager [9]

(*) to whom correspondence should be addressed

[1] CNRS, UMS 3601, Institut Français de Bioinformatique, IFB-core, 2 rue Gaston Crémieux, F-91000, Evry, France
[2] National Life Science Supercomputing Center, Technical University of Denmark, Building 208, DK-2800 Kongens Lyngby, Denmark
[3] Novo Nordisk Foundation Center for Protein Research, Faculty of Health and Medical Sciences, University of Copenhagen, Blegdamsvej 3B, 2200 København, Denmark
[4] Department of Health Technology, Ørsteds Plads, Building 345C, DK-2800 Kongens, Lyngby, Denmark
[5] L'institut du Thorax, INSERM, CNRS, University of Nantes, 44007, Nantes, France
[6] Department of Biochemistry and Molecular Biology and VILLUM Center for Bioanalytical Sciences, University of Southern Denmark, Campusvej 55, 5230 Odense, Denmark
[7] Département de Biologie, Aix-Marseille Université (AMU), 3 place Victor Hugo - 13003 Marseille, France
[8] Computational Biology Unit, Department of Informatics, University of Bergen, N-5008 Bergen, Norway
[9] Hub de Bioinformatique et Biostatistique – C3BI, Institut Pasteur, USR 3756, CNRS, Paris (75015), France

Jon Ison https://orcid.org/0000-0001-6666-1520
Hans Ienasescu https://orcid.org/0000-0001-9727-2544
Piotr Chmura https://orcid.org/0000-0002-9371-6918
Alban Gaignard https://orcid.org/0000-0002-3597-8557
Veit Schwämmle https://orcid.org/0000-0002-9708-6722
Jacques Van Helden https://orcid.org/0000-0002-8799-8584
Matúš Kalaš https://orcid.org/0000-0002-1509-4981
Hervé Ménager https://orcid.org/0000-0002-7552-1009

[1] These authors contributed equally to this work.

Abstract

Background - Life scientists routinely face massive and heterogeneous data analysis tasks, and must find and access the most suitable databases or software in a jungle of web-accessible resources. The diversity of information used to describe life-scientific digital resources presents an obstacle to their utilisation. Although several standardisation efforts are emerging, no information schema has been sufficiently detailed to enable uniform semantic and syntactic description - and cataloguing - of bioinformatics resources.

Findings - Here we describe *biotoolsSchema*, a formalised information model which balances the needs of conciseness for rapid adoption, while still providing rich technical information and scientific context. *biotoolsSchema* results from a series of community-driven workshops, and is deployed in the *bio.tools* registry, providing the scientific community with more than 17,000 machine-readable and human-understandable descriptions of software and other digital life-science resources. We compare our approach to related initiatives and provide alignments to foster interoperability and reusability.

Conclusions - *biotoolsSchema* supports the formalised, rigorous and consistent specification of the syntax and semantics of bioinformatics resources, and enables cataloguing efforts such as *bio.tools* that help scientists to find, comprehend and compare resources. The use of *biotoolsSchema* in *bio.tools* promotes the FAIRness of research software; a key element of open and reproducible developments for data-intensive sciences.

Availability and implementation: https://github.com/bio-tools/biotoolsschema
Contact: jon.c.ison@gmail.com
Supplementary Information: http://biotoolsschema.readthedocs.io/

Background

Workers in the life sciences must routinely describe, organise, find, understand, compare, select, use and connect a large and diverse set of analytical tools and data resources. These tasks can benefit greatly from detailed and consistent resource descriptions which are, when available, human-readable and, ideally, machine-readable. Consider for example the following tasks:

> **T1:** A scientist surveying recently published tools in a general scientific area or for a specific computational task, highlighting those which are freely accessible.
>
> **T2:** A bioinformatician constructing a data analysis pipeline, and searching for tool alternatives which perform a given operation on a specific type of biological data available in a particular format.

T3: A web developer tasked with building a portal to catalogue and promote the software outputs of a scientific community or consortium.

T4: A project manager assessing the software contributions including scientific impact of a particular project, institution, individual or research grant.

T5: A software developer wishing to contribute to open-source software projects, or seeking to claim credit for and promote their own contributions and productions.

These tasks can be challenging due to a lack of community-agreed standards or best practices to describe life science software and data resources. Even if open source software developers document their code for better (re)usability, the provided information may address very different aspects, with very different granularity levels. For instance, T1 would require the tool publication date, as well as its usage license, to be available and machine-readable. In practice, a common strategy is to manually search and browse a large variety of web pages, ranging from software-oriented resources (*e.g.* GitHub) to scientific literature resources (*e.g.* PubMed), sometimes through specific form-based search engines. Survey tasks are time consuming and often require repeated, and sometimes complex searches. As for T2, searching for tool alternatives is also challenging. In the best cases, software developers/providers precisely describe their contributions. But it is often difficult to compare two tools for a similar data analysis task because of the heterogeneity of their description. This would require a tool catalogue (T3) allowing for instance to filter tools based on their application domain, or the type of the data processing they provide. Other issues arise when claiming credit for software contributions (T5) or more generally evaluating scientific impact (T4). Citation recommendations are often provided as a paragraph in a tool's documentation, or using the structured Citation File Format - CFF [1]. Automated retrieval of such citation recommendations would be particularly useful in the context of virtual research environments where life scientists combine bioinformatics tools into data-intensive workflows.

All of these tasks depend highly on the availability of a shared human-understandable and machine-processable controlled vocabulary and syntax to precisely describe bioinformatics software and data resources. We thus propose *biotoolsSchema*. Our objective is twofold: (1) provide a technical means to formalise and express rich bioinformatics resource metadata required to achieve at least tasks T1-5, and (2) provide incentives for bioinformatics resource providers to enrich their tool metadata for better human/machine accessibility, readability and reusability. *biotoolsSchema* is a formalised information model that puts the description of a broad range of bioinformatics resources on a rigorous and consistent syntactic and semantic basis. Our model is developed through a community effort and has evolved steadily since its origin in the BioMedBridges project (concluding in 2015) [2], and more recently during its

development for ELIXIR [3], resulting in the latest stable version 3.3.0. In *Comparison to related efforts* we introduce and compare *biotoolsSchema* to various relevant software metadata initiatives, in context of providing stable solutions to maintain FAIR principles [4] between software providers and consumers.

biotoolsSchema is broadly applicable, but optimised to describe bioinformatics *tools* - application software with well-defined data processing functions (inputs, outputs and operations). This includes simple tools with one or a few closely related functions, and complex, multimodal tools with many functions, available for immediate use as online services, or in a form which users can download, install, configure and run themselves.

biotoolsSchema defines 50 scientific, technical and administrative attributes. It concentrates upon salient common features, necessary and sufficient for the systematic cataloguing and use of tool information in a variety of contexts. Internally, the EDAM ontology [5] enables rigorous and consistent description of tool functionalities (see "Model of tool function"), such that tools can be readily found, comprehended and compared by typical software end-users.

biotoolsSchema is available as XML Schema (XSD) and JSON Schema variants, and can be used to validate corresponding tool descriptions in XML, JSON and YAML formats. We summarise the design, methods and implementation of *biotoolsSchema*, comparing it to complementary approaches. We also summarise its applications, including the description of a dataset of over 17,000 tools registered in the *bio.tools* registry (https://bio.tools) [6].

Findings

Scope

biotoolsSchema is applicable to a nearly complete range of application software, including command-line tools, scripts, libraries, workflows, web applications, database portals, web APIs, web services, SPARQL endpoints, desktop applications, plug-ins, workbenches and suites. These tool types and their definitions were settled following an analysis of *bio.tools* and are included as a controlled vocabulary within *biotoolsSchema* (see *Controlled vocabularies*). They are intended to provide a practical and intuitive designation. In principle, when describing a tool, one or more tool types may be assigned, reflecting the different facets of the software being described. *biotoolsSchema* includes general attributes such as tool description, publication and license. Execution-layer information, for example command-line tool options or web service endpoints, are out of scope. *biotoolsSchema* thus complements for example Galaxy [7], the Common Workflow Language (CWL) [8], or Boutiques [9] command-line tool descriptions, and OpenAPI descriptions of web services.

Software attributes

biotoolsSchema covers a total of 50 scientific, technical or administrative software attributes, organised for convenience into 9 logical groupings (Figure 1, Table 1). To support the broadest range of applications, only bare-bones metadata (name, short description and homepage) are mandated, the rest of the attributes (Table 2) being conditionally required or optional. The small mandatory core of elements was also a practical necessity for the curation of bio.tools at a very large scale, to facilitate the registration of new entries which can be subsequently improved by the author or the broader community. Element cardinality constraints (1 only, 1 to many, 0 or 1, 0 to many) were chosen to provide flexibility, where applicable. To enable concise information, standard identifiers are used where possible, *e.g.* DOIs for publications, Open Researcher and Contributor IDs (ORCIDs) for people [10], ontology concept IDs for specialised scientific aspects, and controlled vocabularies for other attributes (see *Controlled vocabularies*). Verbose information, for example, software documentation, terms of use or citation instructions, are referred to by URL. Regular expression patterns are defined on all applicable elements to support precise syntax validation.

Figure 1. *biotoolsSchema* overview
Software attributes are organised into 9 groups (in boxes), and include terms from controlled vocabularies defined internally within biotoolsSchema*, standard identifiers (including from the EDAM ontology), links or free text. Cardinality of the groups and attributes is shown in superscript and in the block arrows.*

Table 1. Software attribute groups

Group	XML element	Description
Summary	-	Basic information about the software.
Labels	-	Miscellaneous scientific, technical and administrative details of the software, expressed in terms from controlled vocabularies.
Functions	`function`	Details of the function(s) (*i.e.* modes of operation) the software provides, expressed in concepts from the EDAM ontology.
Links	`link`	Miscellaneous links for the software *e.g.* repository, issue tracker or mailing list.
Downloads	`download`	Links to downloads for the software, *e.g.* source code, virtual machine image or container.
Documentation	`documentation`	Links to documentation about the software *e.g.* user manual, API documentation or training material.
Relationships	`relation`	Details of a relationship this software has to other software registered in *bio.tools*.
Publications	`publication`	Publications about the software.
Credits	`credit`	Individuals or organisations that should be credited, or may be contacted about the software.

Software attributes are grouped within biotoolsSchema*. The groups correspond to XML elements with the exception of 'Summary' and Labels' groups.*

Table 2. Software attributes
<<< SEE END OF DOCUMENT >>>

biotoolsSchema *covers 50 general software attributes grouped for convenience. EDAM concepts may be specified by one or both of an URI or term. "enum" indicates a controlled vocabulary defined by* biotoolsSchema*. Attributes of type xs:token or URL include regular expressions for syntax validation, where applicable.*

Scientific concepts

The EDAM ontology [5] provides the core vocabulary for the scientific description of tools including types of data and data identifiers, data formats, operations and topics. EDAM organises these concepts into the EDAM Topic, Operation, Data and Format sub-ontologies. Concepts may be specified by one or both of an EDAM concept URI (*e.g.* http://edamontology.org/topic_0121) and/or a term (*e.g.* "Proteomics") - a preferred label or synonym of a concept from the appropriate EDAM sub-ontology. It is strongly recommended to specify at least the URI, as these persistently identify a concept (labels and synonyms can change).

Model of tool function

The model of tool functionality (Figure 2) is concise and simple. It supports a practical summary of a tool's essential functionality including primary inputs and outputs from the perspective of a typical biologist end-user. Each software entity may have one or more functions, each corresponding to a mode of operation that the software provides. In turn, each function performs one or more basic operations, and has zero or more primary input and/or output data. Each input or output is of a specified data type and has supported format(s). Operation (*e.g.* "Sequence alignment"), data type (*e.g.* "Sequences") and format (*e.g.* "FASTA") are EDAM concepts. An optional comment, and relevant command, command-line fragment or option for executing the function, may also be specified, mainly to facilitate function identification in tools which can perform multiple operations.

Figure 2. Model of tool function

biotoolsSchema *follows a simple model of tool function, where each function (mode of operation) performs one or more specific operations. Each function may have one or more primary inputs and outputs, each of a defined data type and listing supported format(s). Illustration is for the ProCon* (biotools:procon) *conversion utility.*

Auxiliary information

Miscellaneous links, downloads and documentation are modelled in a common way (Figure 3) including a URL, a type, and an optional comment. Specifying the types of documentation *etc.* via controlled vocabularies allows these to be extended in the future, in a way that is non-breaking to schema dependencies.

Figure 3. Model of links, downloads and documentation

Links, downloads and documentation are modelled in a common way; a URL which is annotated to indicate facets (such as issue tracker, code repository etc.*) and an optional comment. Additionally, downloads also allow for associated version information. Illustration is for miscellaneous links for the ProCon* (biotools:procon) *conversion utility.*

Publications
Relevant publications must be specified by (at least) one of a DOI (Digital Object Identifier), PMID (PubMed reference number) or PMCID (PubMed Central reference number), and may be optionally typed, *e.g.* “Review”. Use of DOIs - the most generic of these identifiers - is recommended.

Tool relationships
Relationships between tools that have been registered in *bio.tools* may be specified by biotoolsID and a term from a controlled vocabulary which is currently limited to *isNewVersion*/*hasNewVersion* (version relationships), *uses*/*usedBy* (general functional association) and *includes*/*includedIn* (primarily for associating collections such as software suites with their constituent tools). These relationship types will be extended in due course.

Credits and contact information
Credits and contacts for a tool are handled by a consolidated mechanism. Creditable or contactable entities of various types (“Person”, “Institute” *etc.*) and roles (“Developer”, “Support” *etc.*) must have at least one or more of a name, email and URL. ORCIDs provide a persistent reference to information on an individual person. Global Research Identifier Database Identifiers (GRID IDs) and Research Organization Registry Identifiers (ROR IDs) are used for organisations, and Crossref Funder Registry Identifiers (FundRef /Funder IDs) for funding organisations. Specification of these IDs, where available, is strongly recommended, as these enable sustainable maintenance and reuse of relevant metadata.

Controlled vocabularies

In addition to EDAM, a further 18 controlled vocabularies (Table 3) catering for technical aspects are defined internally within *biotoolsSchema* as *standardised enumerations of terms*. Notably the license controlled vocabulary uses identifiers from the industry standard SPDX list [11]. Comprehensive documentation (see *Documentation*) including definitions of terms in each vocabulary is available online (*https://biotoolsschema.readthedocs.io/en/latest/controlled_vocabularies.html*) and is embedded in the chema file (XSD variant only).

Table 3. Controlled vocabularies

**Controlled vocabulary (#terms)**	**Description**
identifier type (4)	The type of tool identifier, *e.g.* “doi”
tool type (15)	The type of application software, *e.g.* “Command-line tool”
operating system (3)	The operating system supported by a downloadable software package, *e.g.* “Linux”
programming language (57)	Name of programming language the software source code was written in, *e.g.* “C”

license (326)	Software or data usage license, *e.g.* "GPL-3.0"
maturity (3)	How mature the software product is, *e.g.* "Mature"
cost (3)	Monetary cost of acquiring the software, *e.g.* "Free of charge"
accessibility (3)	Whether there are non-monetary restrictions on accessing an online service, *e.g.* "Open access"
elixirPlatform (5)	ELIXIR research infrastructure technical platform, *e.g.* "Tools"
elixirNode (22)	ELIXIR research infrastructure national node, *e.g.* "France"
elixirCommunity (11)	Name of relevant ELIXIR (or other) community, *e.g.* "Galaxy"
link type (12)	The type of data, information or system that is obtained when the link is resolved, *e.g.* "Helpdesk"
download type (18)	Type of download that is linked to, *e.g.* "Source code"
documentation type (15)	Type of documentation that is linked to, *e.g.* "API documentation"
publication type (6)	Type of publication, *e.g.* "Review"
relation type (6)	Type of tool relationship, *e.g.* "uses"
credit entity type (6)	Types of entities that may be credited, *e.g.* "Person"
credit entity role (7)	Roles that may be assigned to creditable entities, *e.g.* "Developer"

biotoolsSchema defines 18 controlled vocabularies catering for technical aspects of software description.

Implementation of *biotoolsSchema* in *bio.tools*

bio.tools (https://bio.tools) provides the means - manually via graphical user interfaces and programmatically via a REST API - for a user to browse and search over *biotoolsSchema*-formatted data, and to add to, edit or download the registry content. Tool description data registered or downloaded via the REST API in a choice of serialisation formats (XML, JSON or YAML) are compatible with *biotoolsSchema*. *bio.tools* provides unique, persistent and immutable tool identifiers (*e.g.* "signalp", biotools:signalp). These identifiers are used in persistent *bio.tools* URLs (*e.g.* https://bio.tools/signalp), resolving to Tool Cards which summarise essential tool information. The *bio.tools* compact URIs (*e.g.* "biotools:signalp") are a convenient short form; simply the identifier in the "biotools" namespace. *biotoolsSchema* supports other types of identifier, and software version information may be attached to the entire tool description, or to a specific identifier, download or publication, and specified in a flexible way allowing for example a single version label, or a list or range of labels to be annotated. In case a single label annotation reflects a rigorous assignment of software version made by the tool developer, this can be used in conjunction with the *bio.tools* tool ID to uniquely identify a particular software artefact.

As of September 2020, *bio.tools* includes 17,370 entries and a total of 301,956 individual annotations, including 101,517 references to concepts from the EDAM ontology, as per attributes defined within *biotoolsSchema*. Individual tool

descriptions vary in richness, and are being progressively improved, through an initiative that engages the community with the curation process [12, 13]; for example producing a high-quality tools description corpus for proteomics data analysis [14]. The *bio.tools* content, user interfaces and API will be described in more details in a future publication.

Serialisation formats and transformations

bio.tools supports upload and download of *biotoolsSchema*-formatted data in a choice of serialisation formats (XML, JSON or YAML). XML support in *bio.tools* was developed using XSLT transformations to support two-way, lossless interconversions between *biotoolsSchema*-formatted XML files and the JSON, YAML and generic XML formats that are natively supported by the Django web framework used by *bio.tools*. This offers maximum flexibility to providers and consumers of *biotoolsSchema*-formatted data, allowing for rigorous validation (against the XSD) irrespective of favoured format. The transforms are freely available from https://github.com/bio-tools/biotoolsShim/. For illustration purposes, a sample JSON file for the SignalP command-line tool (biotools:signalp) (Figure 4) is shown. We also recently created conversion code to support the lightweight JSON-LD format of the Bioschemas [15] Tool profile, which is now available through the *bio.tools* API.

Figure 4. Sample JSON file for signalp tool


```
{
	"name": "SignalP",
	"description": "Prediction of the presence and location of signal peptide cleavage sites in amino acid sequences from different organisms.",
	"homepage": "http://cbs.dtu.dk/services/SignalP/",
	"biotoolsID": "signalp",
	"biotoolsCURIE": "biotools:signalp",
	"version":
	[
		"4.1"
	],
	"otherID": [
		{
			"value": "rrid:SCR_015644",
			"type": "rrid"
		}
	],
	"toolType":
	[
		"Command-line tool",
		"Web application"
	],
	"topic":
	[
		{
			"uri": "http://edamontology.org/topic_3510",
			"term": "Protein sites, features and motifs"
```

```
            }
        ],
        "operatingSystem":
        [
            "Linux",
            "Mac"
        ],
        "license": "Other",
        "collectionID":
        [
            "CBS"
        ],
        "maturity": "Mature",
        "cost": "Free of charge (with restrictions)",
        "function":
        [
            {
                "operation":
                [
                    {
                        "uri": "http://edamontology.org/operation_0418",
                        "term": "Protein signal peptide detection"
                    },
                    {
                        "uri": "http://edamontology.org/operation_0422",
                        "term": "Protein cleavage site prediction"
                    }
                ],
                "input":
                [
                    {
                        "data":
                        {
                            "uri": "http://edamontology.org/data_2044",
                            "term": "Sequence"
                        },
                        "format": [
                            {
                                "uri":
    "http://edamontology.org/format_1929",
                                "term": "FASTA"
                            }
                        ]
                    }
                ],
                "output":
                [
                    {
                        "data": {
                            "uri": "http://edamontology.org/data_1277",
                            "term": "Protein features"
                        },
                        "format":
                        [
                            {
```

```
                                        "uri":
"http://edamontology.org/format_2305",
                                        "term": "GFF"
                                    }
                                ]
                            },
                            {
                                "data":
                                {
                                    "uri": "http://edamontology.org/data_2955",
                                    "term": "Sequence report"
                                }
                            }
                        ],
                        "note": "predicts the presence and location of signal peptide
cleavage sites in amino acid sequences from different organisms"
                    }
                ],
                "link":
                [
                    {
                        "url": "http://www.cbs.dtu.dk/cgi-bin/sw_request?signalp",
                        "type":
                        [
                            "Repository"
                        ]
                    }
                ],
                "download":
                [
                    {
                        "url": "http://www.cbs.dtu.dk/cgi-bin/sw_request?signalp",
                        "type": "Source code",
                        "note": null,
                        "version": null
                    },
                    {
                        "url": "http://www.cbs.dtu.dk/cgi-bin/sw_request?signalp",
                        "type": "Binaries",
                        "note": null,
                        "version": null
                    }
                ],
                "documentation":
                [
                    {
                        "url": "http://www.cbs.dtu.dk/services/SignalP",
                        "type":
                        [
                            "General"
                        ]
                    }
                ],
                "publication":
                [
                    {
```

```
                "doi": "10.1038/nmeth.1701",
                "pmid": "21959131",
                "type":
                [
                      "Primary"
                ]
          }
    ],
    "credit":
    [
          {
                "name": "TN Petersen",
                "typeEntity": "Person",
                "typeRole":
                [
                      "Developer"
                ]
          },
          {
                "name": "CBS",
                "typeEntity": "Institute",
                "typeRole":
                [
                      "Provider"
                ]
          },
          {
                "name": "Henrik Nielsen",
                "email": "hnielsen@cbs.dtu.dk",
                "orcidid": "http://orcid.org/0000-0002-9412-9643",
                "typeRole":
                [
                      "Developer"
                ]
          },
          {
                "name": "Henrik Nielsen",
                "email": "hnielsen@cbs.dtu.dk",
                "orcidid": "http://orcid.org/0000-0002-9412-9643",
                "typeEntity": "Person",
                "typeRole":
                [
                      "Primary contact"
                ]
          }
    ]
}
```


Comparison to related efforts

Various research infrastructure or community-led initiatives (Table 4) have defined, or are in the process of defining, sets of information fields to describe bioinformatics software application metadata. The *HCLS Community Profile* [16]

was an early effort of the Semantic Web Health Care and Life Sciences Interest Group [17]. It specifies dataset descriptions using the Resource Description Framework (RDF), using 24 core metadata elements and recommends re-use of various well established, general-purpose RDF controlled vocabularies including Dublin Core [18], Friend-of-a-Friend [19] and PROV [20]. We will describe in a future publication *biotoolsRDF* (https://github.com/bio-tools/biotoolsRdf), an application ontology that defines the OWL2 Web Ontology Language encoding of *biotoolsSchema*. Like the HCLS Community Profile, it re-uses other well established vocabularies wherever possible, but provides a richer set of attributes and is specifically geared towards application software metadata rather than datasets in general. Tangential to these is the *Schema.org vocabulary*, founded by the major web search engine providers. It is an exhaustive controlled vocabulary used to annotate contents of web pages, and is organised into a hierarchy of a very broad range of conceptual classes. It includes concepts relevant to software such as *SoftwareApplication* and *CreativeWork,* and is well suited for general purpose, lightweight mark-up of web pages for discovery purposes. This is in contrast to *biotoolSchema* which is tailored specifically to detailed descriptions of application software especially, and mandates stricter syntax and semantics. A *Tool Profile*[2] currently under development for the Bioschemas project [15] will provide guidelines on the consistent adoption of Schema.org markup for the description of software tools in life sciences, including for example recommending the use of EDAM ontology for scientific aspects. The emerging Tool Profile is fully compatible with *biotoolsSchema*, and we have implemented it in *bio.tools*, which now serves via the API a Bioschemas serialisation of the *bio.tools* content.

Parallel to initiatives depending upon *Semantic Web* technologies, are efforts reflecting existing practice or the requirements of various research infrastructures. The Citation File Format, CFF [1] is a YAML format for general-purpose software annotations. Its focus is to support all citation-specific use cases for the citation of software, and thus promote attribution and credit of research software. It provides more detail in this area than *biotoolsSchema*, but lacks attributes for example around tool functionality which are a focus of *biotoolsSchema*. The *DataCite Metadata Schema* from DataCite [21] - a non-profit organisation that provides persistent identifiers (DOIs) for research data - includes core metadata properties primarily for resource identification, citation and retrieval, encapsulated in an XML schema with usage guidelines. The *Application Profile* included in the *Guidelines for Software Repository Managers* from OpenAIRE [22] - a European project supporting Open Science - is based on DataCite and covers 23 software attributes, primarily to make software products citable. Similarly, the *Software Citation Principles* produced by FORCE11 community initiative

[2] https://github.com/BioSchemas/specifications/tree/master/Tool

[23], define 11 basic metadata requirements for software citation. The SciCrunch registry [24] shares a similar purpose to *bio.tools*, but uses an RDF-based data model. Their scope is similar, but *biotoolsSchema* enables, through its use of EDAM, the end-user to drill down to fine-grained aspects of tool functionality such as specific inputs, outputs and operations. SciCrunch uses persistent Research Resource Identifiers (RRIDs) [25] which in case of computational tools are equivalent to *bio.tools* tool IDs. The *eInfraCentral Service Description Template* produced by the European E-Infrastructure Services Gateway, eInfraCentral [26], is (as a work in progress) defining the information requirement for a common E-Infrastructures service catalogue, that will describe and offer services to end-users in a harmonised way, through the European Open Science Cloud (EOSC) portal [27]. ELIXIR is involved in the Tools Collaboratory for the EOSC-Life project (https://www.eosc-life.eu/), which will drive the development of an environment enabling the cloud deployment of workflows for the analysis and integration of life science data. We anticipate *biotoolsSchema*-formatted descriptions of tool functionality will contribute to this environment, especially to workflow composition and the production of applicable registries.

The CodeMeta specification [28] is a more generalised approach, and was developed as a lightweight format to describe scientific software, based on an extension of Schema.org using JSON-LD. A major component is the *CodeMeta Metadata Crosswalk,* produced by the CodeMeta community project [29], which is a table reflecting a comparison of software metadata used across multiple code repositories and systems. The crosswalk (a work in progress) yields an exhaustive set of 65 software metadata concepts (mostly mapped to Schema.org concepts), and can inform efforts to produce a minimal concept vocabulary for software reflecting a consensus in the mappings. *biotoolsSchema* has been submitted as a CodeMeta crosswalk (see below), and covers many of the common concepts, despite these not always being explicitly mapped owing to technical limitations of the how the crosswalk is currently represented.

Table 4. Software metadata initiatives

Initiative	Description
HCLS Community Profile	Specification for dataset description using RDF. https://www.w3.org/TR/hcls-dataset/
eInfraCentral Service Description Template	Information model for European eInfrastructure services (including software services). https://jnp.gitbooks.io/service-description-template-v1-12/basic_service_information/
DataCite Metadata Schema	XML schema and guidelines of core metadata properties for resource identification, citation and retrieval. https://schema.datacite.org/
OpenAIRE Application Profile	Guidelines for software repository managers. https://software-guidelines.readthedocs.io/en/latest/application_profile.html

CodeMeta Metadata Crosswalk	Vocabulary for software metadata concepts and crosswalk between software metadata projects. https://github.com/codemeta/codemeta/blob/master/crosswalk.csv
Schema.org Vocabulary	Controlled vocabulary for marking up web pages. https://schema.org
Bioschemas Tool Profile	Schema.org specification for tools in the Life Sciences. https://bioschemas.org/devSpecs/Tool/
FORCE11 Software Citation Principles	Basic metadata requirements for software citation. https://www.force11.org/software-citation-principles

Various initiatives for software metadata of relevance to biotoolsSchema *are shown.*

This summary of initiatives is not exhaustive. Others include the DOE CODE initiative [30] for code archiving by the U.S. Department of Energy (DOE), and guidelines [31] for rich search results for software from Google, and specialised ontologies for software including SWO [32] and OntoSoft [33], each serving a different use case. We have thus a plethora of different recommendations and ways to annotate and share software metadata. The diversity reflects a wide range of perspectives, use cases and contexts, but brings the challenge of curating software metadata and sharing it between systems, whilst avoiding inconsistencies and duplication of efforts. We ameliorate this interoperability issue, at least so far as sharing and re-using *bio.tools* metadata, through an exhaustive crosswalk (Table 5) between *biotoolsSchema* elements and key software metadata initiatives including CodeMeta, Schema.org, OpenAire, DataCite, HCLS, eInfraCentral, FORCE11 and miscellaneous RDF vocabularies. Each element in *biotoolsSchema* was mapped, where possible, to the corresponding field used by these initiatives, and the mappings aggregated, discussed and reviewed, resulting in a consolidated crosswalk (Table 5) for *biotoolsSchema*, that has been submitted to the CodeMeta. The crosswalk thus provides a framework useful to any engineer integrating software metadata provided in these contexts.

Table 5. Comparison of *biotoolsSchema* and other software metadata initiatives

<<< SEE END OF DOCUMENT >>>

Elements in biotoolsSchema *are mapped to equivalent elements from various software metadata initiatives. Only those elements which could be mapped are shown.*

Discussion

The efficiency of workers utilising scientific software across the spectrum of the life sciences depends, in a large part, upon high quality and convenient bioinformatics software metadata. *biotoolsSchema*, in combination with EDAM, provides a formalised, rigorous and consistent specification of the syntax and semantics for these metadata. This enables software developers and service providers to define their productions in a consistent way (tasks T4, T5),

cataloguers to communicate clearly what is available (tasks T1, T3), and software end-users to more efficiently use these resources (task T2). For example, a recent study [34] demonstrated the usefulness of *biotoolsSchema*-formatted data for automated workflow composition in mass spectrometry-based proteomics data analysis. Here, *biotoolsSchema*, through its use of the EDAM ontology, enabled the precise annotation of tool inputs, outputs and operations that was critical for workflow synthesis. *biotoolsSchema* thus encompasses diverse use cases, from provenance through to query and discovery, and can help to standardize the curation and exchange of metadata across software projects, repositories, initiatives and organizations.

In *biotoolsSchema*, a great complexity of information - including tool functionalities, fields of use, interfaces, deployments, distributions, documentation and so on - is reduced to a manageable and practical level. The model is applicable to nearly all technical types of tool, and supports the uniform description of key scientific, technical and administrative attributes. Specifically, in line with tasks T1 and T3 addressing tools surveys and community-oriented registries, it allows for a presentation and comparison of tool information, which often cannot conveniently be obtained from a Google search or cursory inspection of a provider’s website. With progressive development, *biotoolsSchema* applications such as *bio.tools* will help to make complex tool functions more easily understood, and render tools more accessible, usable and interoperable, *i.e.* more FAIR [4]. For example, a study [35] showed how formalized tool descriptions could be reused and bridged to workflow provenance to provide user and machine-oriented data summaries. This complement efforts such as Boutiques [9] which have been applied to the neuroimaging analysis domain. We plan to evaluate generically the FAIRness of tools registered in *bio.tools*, once there is broad community agreement on a suitable set of metrics. For a start, we examined the criteria defined in “Towards FAIR principles for research software" [36] with respect to the features of *bio.tools* and *biotoolsSchema*. This comparison (Table 6) shows how in practice the use of *biotoolsSchema* through *bio.tools* already helps to improve the FAIRness of tools. The table also informs possible FAIR metrics, which can be encapsulated using our emerging Tool Information Profile system [37] and used to provide an objective, transparent and flexible framework to evaluate tool FAIRness.

Table 6. Role of bio.tools and biotoolsSchema in promoting software FAIRness

<<< SEE END OF DOCUMENT >>>

For each FAIRness criteria for software (in column 1) as proposed in [36], the role of bio.tools *(column 2) and* biotoolsSchema *(column 3) are summarised. Some of these criteria, such as the assignment of an identifier, are satisfied by a* bio.tools *registration, while other criteria such as the license depend upon the curation of a* biotoolsSchema *attribute within* bio.tools*.*

Our standalone schema allows for community development of the model to be loosely coupled to applications such as *bio.tools*, and provides a means for an end-user to validate content external to any system, ensuring correct syntax, structure and completeness (T3). *biotoolsSchema* must evolve to keep pace with developments in the field and support new applications and integration scenarios. This may include richer modelling of the complex relationships between resources, and support for specialised biological ontologies such as Gene Ontology [38] for molecular function, cellular component and biological process, Sequence Ontology [39] for genomic elements, and NCBI Taxonomy [40] for taxa. *biotoolsSchema* will thus provide a means to relate a large set of tools such as in *bio.tools* to a broader and flourishing ecosystem of workflows, databases and ontologies.

Future changes will be pragmatic, driven by community use cases, and in light of practical experience of what data is useful and readily available. No single model, registry or initiative can hope to cover all bases. *biotoolsSchema* can be augmented by (and will not duplicate) the functionalities of related well-maintained models provided by more specialised initiatives, *e.g.* execution-layer information about command-line tools provided by CWL, or information about service endpoints supported by OpenAPI [41]. More specifically, we plan to build upon previous work [42-45] to improve the cross-linking and cross-enrichment of execution-oriented tool descriptions with *biotoolsSchema* data. Volatile attributes, or attributes which must be frequently recalculated, such as metrics of usage, technical performance data, links to similar tools, software dependencies, hardware requirements, and so on, will remain out of scope.

Different software metadata use cases have different information requirements. *biotoolsSchema* supports very minimal or much more comprehensive information specifications, according to needs, without imposing a high curation burden and thus a barrier to adoption. It provides the basis for, but cannot in itself specify, a flexible information requirement suited to diverse purposes and contexts, *e.g.* curation of registries such as *bio.tools*, required information for service delivery plans, publication of software articles, or metrics for software metadata or project quality. For such purposes, a framework [46] for tool information requirements is under development ("Tool Information Profiles"), which is based on *biotoolsSchema*, but goes beyond the syntactic / semantic constraints that can conveniently be defined in XML schema. Human-readable guidelines for curation of software metadata are also being developed as part of an emerging Curators Guide [47].

biotoolsSchema, with progressive development and adoption, can benefit the whole bioinformatics community. It can help to support best practices promoted by various research infrastructures which emphasize the value of bioinformatics software registries for findability [48, 49], and bridge the gap between technology-oriented developers and service-oriented research infrastructures and organisations. The field of software metadata management is socially and technically very complex, and includes many more stakeholders and perspectives than are summarised here, with

multiple projects serving different but overlapping needs, use cases, and contexts. We encourage all such efforts and warmly welcome collaborations for the continued development of *biotoolsSchema*, its applications and integration into the broader bioinformatics ecosystem.

Methods

Design considerations

Requirements were established during a series of community-led workshops resulting in ten founding principles and design considerations, now implemented as characteristics of *biotoolsSchema*:

- ***Practical*** - focus on salient attributes of practical value in everyday use; especially to support the discovery, use and practical interoperability of software; superfluous details are excluded.
- ***General*** - generally applicable, *i.e.* to all manner of bioinformatics resources (see *Scope*).
- ***Consistent*** - use ontologies and standardised enumerations of terms (see *Controlled Vocabularies*) where possible, to support precise searches over *biotoolsSchema*-formatted data and return of consistent and therefore comparable information.
- ***Concise*** - mandate URLs or standard identifiers where possible, helping to ensure the sustainable upkeep of *biotoolsSchema*-formatted data and support future integrations, applications and cross-linking with other resources.
- ***Simple*** - *biotoolsSchema* is as flat (unstructured) as is practicable, ensuring ease of use, whilst preserving essential structure, *e.g.* a meaningful model of tool function.
- ***Compatible*** - it is inevitable that tool providers, integrators, and cataloguers will continue to use a variety of models, methods and formats for tool descriptions; *biotoolsSchema* is broadly compatible (see *Comparison to related efforts*) to support future integration scenarios.
- ***Extensible*** - to cater for emerging requirements, and adaptable by others for their own purposes (see *Development process and status*).
- ***Stable*** - the maintenance of software dependencies on mutating schema is expensive. Backwards incompatible changes are only made if absolutely required (see *Development process and status*).
- ***Free and open source*** - to encourage reuse and new applications.
- ***Community-driven development*** - to ensure end-user needs are satisfied.

Development process and status

The model is an evolution of an early prototype developed for BioMedBridges, that began in 2012. Its evolution has been tightly linked with that of EDAM and *bio.tools*, as these projects together are the key components of an ecosystem [13] that enables communities, projects and individuals to describe and share their own bioinformatics resources. *biotoolsSchema* had multiple successive development iterations, most of which were based on community events such as workshops and hackathons[3]. Such events typically involved the gathering of developers from the ecosystem described, together with members from domain (*e.g.* Proteomics) or project-specific (*e.g.* Debian Med) communities. The events allowed us to combine contributions such as the addition of contents by domain experts, with the collection of feedback and requirements on EDAM, *biotoolsSchema* and *bio.tools*. Whenever possible, we also included downstream activities, including requests management using agile techniques such as priority poker, and development and debugging activities.

In parallel, development was informed by growth in *bio.tools*: major content providers and other end-users have helped to validate the model, with the registry itself providing a valuable dataset for this purpose. Thus, we consider the stable version (3.3.0) to satisfy major community requirements, and provide a solid foundation upon which the content, functions, integration and applications of portals such as *bio.tools* can be built. The model must be subject to future improvements and the schema is extensible; both the number and type of attributes can evolve, according to end-user requirements. In order to provide stability for developers and software dependencies, major changes are restricted to approximately yearly cycles in released stable versions. Future versions will not depart fundamentally from the attributes or structure described in this article. From version 3.0.0, version numbers follow the SemVer 2.0.0 scheme (https://semver.org/). All developments are tracked at GitHub (https://github.com/bio-tools/biotoolsschema); feedback, contributions and collaboration are welcome.

biotoolsSchema is a community-driven project governed under the leadership of the French ELIXIR Node (Jacques van Helden, Joint Head of Node) in collaboration with partners within and beyond ELIXIR, ensuring its sustainability. For further information see https://biotoolsschema.readthedocs.io/en/latest/contributors.html.

Documentation

The schema is comprehensively and consistently documented:

- textual (human-readable) description of each schema element

---

[3] The online documentation of *bio.tools* includes a non-exhaustive list of such events: https://biotools.readthedocs.io/en/latest/events.html.

- additional, highly concise element descriptions, suitable for example as tips in user interfaces
- definition of terms in all controlled vocabularies
- mapping of schema elements including controlled vocabularies to other relevant models and vocabularies
- usage information including technical details (such as syntax and use of *bio.tools* API) and curation guidelines (good practice on using *biotoolsSchema* to describe tools)
- information about the project and community

The documentation is, where possible, encoded within the XSD and JSON schemas, but also made available online in a more user-friendly form:

- https://biotoolsschema.readthedocs.io/ (project docs)
- http://bio-tools.github.io/biotoolsSchema/ (technical docs)
- https://bio-tools.github.io/biotoolsSchemaJ/ (technical docs for the JSON schema variant)
- https://biotools.readthedocs.io/en/latest/api_usage_guide.html (*bio.tools* API user guide)
- http://biotools.readthedocs.io/en/latest/curators_guide.html (*bio.tools* curation guide)

Availability and requirements

biotoolsSchema is licensed under a Creative Commons Attribution-ShareAlike 4.0 International License (CC BY-SA 4.0):

- https://github.com/bio-tools/biotoolsschema

The *bio.tools* content is freely available to all under the Creative Commons Attribution licence (CC BY 4.0) and can be downloaded from *bio.tools*:

- https://bio.tools

An archival copy of supporting data and documentation for biotools schema is also available via the Gigascience repository GigaDB [50].

Funding

This work was supported by funding from the Institut Français de Bioinformatique (IFB / ELIXIR France), the Danish Ministry of Higher Education and Science (ELIXIR Denmark) and from the European Union's Horizon 2020 research and innovation programme (grant agreement no 676559, ELIXIR-EXCELERATE).

Acknowledgements

Jon Ison warmly acknowledges the support of the Institut Français de Bioinformatique. Thanks to Dmitry Repchevsky, Wojtek Dabrowski, and other attendees of ELIXIR workshops for their suggestions.

Author contributions

JI led the development of the schema and article with contributions from all authors. HI, ER and PC implemented the schema in *bio.tools*.

Competing interests

The authors declare no competing interests.

References

[1] Druskat S, Gruenpeter M, Chue Hong N, Silva R, Bast, R; Crusoe M. Citation File Format - CFF. https://zenodo.org/record/1117789#.XtVWBhbRbJU (2017).
[2] Suhr S. et al. REPORT: BioMedBridges workshop on e-Infrastructure support for the life sciences – Preparing for the data deluge. Preprint at http://doi.org/10.5281/zenodo.13942 (2015).
[3] Crosswell LC & Thornton JM. ELIXIR: a distributed infrastructure for European biological data. *Trends in Biotechnology*, **30**(5), 241–242 (2012).
[4] Wilkinson MD et al. The FAIR Guiding Principles for scientific data management and stewardship. *Scientific Data*, **3**, 160018 (2016).
[5] Ison J. et al. EDAM: an ontology of bioinformatics operations, types of data and identifiers, topics and formats. *Bioinformatics*, **29**(10), 1325–1332 (2013).
[6] Ison J. et al. The bio.tools registry of software tools and data resources for the life sciences. Genome Biology **20**, 164 (2019)
[7] Afgan E et al. The Galaxy platform for accessible, reproducible and collaborative biomedical analyses: 2016 update. *Nucleic Acids Research*, **44**(W1), W3–W10 (2016).
[8] Amstutz P. et al: Common Workflow Language, v1.0. Specification, *Common Workflow Language working group*. Preprint at https://doi.org10.6084/m9.figshare.3115156.v2 (2016)
[9] Glatard T, Kiar G, Aumetado Armstrong T, et al. Boutiques: a flexible framework for automated application integration in computing platforms. arXiv preprint arXiv:1711.09713, 2017.
[10] ORCiD website. https://orcid.org/. Accessed 6 June 2020.
[11] Lovejoy J, Odence P, & Lamons S. Advancing the Software Package Data Exchange: An update on SPDX. *International Free and Open Source Software Law Review*, **5**(2), 145–152 (2013).
[12] Ison J. et al. Tools and data services registry: a community effort to document bioinformatics resources. *Nucleic Acids Research*, **44**(D1), D38–D47 (2016).
[13 was10] Ison J. et al. Community curation of bioinformatics software and data resources. *Briefings in Bioinformatics*, **bbz075** (2019)
[14] Tsiamis V., Ienasescu H., Gabrielaitis D, Palmblad M, Schwämmle V. & Ison J. One Thousand and One Software for Proteomics: Tales of the Toolmakers of Science. *Journal of Proteome Research,* **18** (10), 3580-3585 (2019)
[15] Michel F & The Bioschemas Community. Bioschemas & Schema.org: a Lightweight Semantic Layer for Life Sciences Websites. *Biodiversity Information Science and Standards*, **2:e25836** (2018).

[16] Gray AJG et al. The HCLS Community Profile. https://www.w3.org/TR/hcls-dataset. Accessed 6 June 2020.
[17] Semantic Web Health Care and Life Sciences Interest Group. https://www.w3.org/2011/09/HCLSIGCharter. Accessed 6 June 2020.
[18] Dublin Core Metadata Initiative. http://dublincore.org/documents/2003/02/12/dcmi-type-vocabulary. Accessed 6 June 2020.
[19] Friend-of-a-Friend Vocabulary Specification. http://xmlns.com/foaf/spec. Accessed 6 June 2020.
[20] Missier P, Belhajjame K, & Cheney J. The W3C PROV family of specifications for modelling provenance metadata. In *Proceedings of the 16th International Conference on Extending Database Technology - EDBT '13* (2013)
[21] DataCite website. https://www.datacite.org. Accessed 6 June 2020.
[22] OpenAIRE website. https://www.openaire.eu/. Accessed 6 June 2020.
[23] Smith AM, Katz DS, Niemeyer KE, & FORCE11 Software Citation Working Group. Software Citation Principles. *PeerJ Computer Science,* **2:e86 (**2016).
[24] Grethe, J. S., et al. "SciCrunch: A cooperative and collaborative data and resource discovery platform for scientific communities." *Front. Neuroinform. Conference Abstract: Neuroinformatics*. 2014.
[25] Bandrowski, Anita E., and Maryann E. Martone. "RRIDs: a simple step toward improving reproducibility through rigor and transparency of experimental methods." *Neuron* 90.3 (2016): 434-436.
[26] European E-Infrastructure Services Gateway, eInfraCentral. http://einfracentral.eu/. Accessed 6 June 2020.
[27] European Open Science Cloud (EOSC) portal. https://eosc-portal.eu/. Accessed 6 June 2020.
[28] Jones MB et al. CodeMeta: an exchange schema for software metadata. Version 2.0. KNB Data Repository. doi:10.5063/schema/codemeta-2.0.
[29] The CodeMeta project. https://codemeta.github.io. Accessed 6 June 2020.
[30] DOE CODE initiative. https://www.osti.gov/doecode/. Accessed 6 June 2020.
[31] Google Schema.org guidelines for software apps. https://developers.google.com/search/docs/data-types/software-app. Accessed 6 June 2020.
[32] Malone J. et al. The Software Ontology (SWO): a resource for reproducibility in biomedical data analysis, curation and digital preservation. *Journal of Biomedical Semantics,* **5**, 25 (2014).
[33] Yolanda G, Ratnakar V. and Garijo D. OntoSoft: Capturing Scientific Software Metadata. *Proceedings of the Eighth ACM International Conference on Knowledge Capture (K-CAP)*, Palisades, NY (2015).
[34] Palmblad M, Lamprecht AL, Ison J and Schwämmle V. Automated workflow composition in mass spectrometry-based proteomics. *Bioinformatics,* **35**(4):656-664 (2019).
[35] Gaignard A, Skaf-Molli H & Belhajjame K. Findable and reusable workflow data products: A genomic workflow case study. *Semantic Web*, **11** (5), 751-763 (2020)
[36] Lamprecht AL et al. Towards FAIR principles for research software. *Data Science* **3**, 37–59 (2020).
[37] Tool Information Profiles website. https://github.com/bio-tools/Tool-Information-Profiles. Accessed 6 June 2020.
[38] Ashburner M. et al. Gene ontology: tool for the unification of biology. The Gene Ontology Consortium. *Nature Genetics*, **25**(1), 25–29 (2000).
[39] Eilbeck K et al. The Sequence Ontology: a tool for the unification of genome annotations. *Genome Bio*logy **6** (R44) (2005).
[40] Federhen S. The NCBI Taxonomy database. *Nucleic Acids Research*, **40** (Database issue), D136–D143 (2012).
[41] OpenAPI initiative. https://www.openapis.org. Accessed 6 June 2020.
[42] Ménager H, Kalaš M, Rapacki K & Ison J. Using registries to integrate bioinformatics tools and services into workbench environments. *International Journal on Software Tools for Technology Transfer,* **18***,* 581–586 (2016)
[43] Hillion KH, Kuzmin I, Khodak A et al. Using bio.tools to generate and annotate workbench tool descriptions [version 1; peer review: 4 approved]. *F1000Research*, **6** (ELIXIR):2074 (2017)
[44] Willighagen E & Mélius J. Automatic OpenAPI to Bio.tools Conversion. Preprint at https://doi.org/10.1101/170274 (2017)
[45] Doppelt-Azeroual O, Mareuil F, Deveaud E, et al. ReGaTE: Registration of Galaxy Tools in Elixir. *Gigascience*, **6**, 1-4 (2017)
[46] Tool Information Profiles. https://github.com/bio-tools/tool-Information-profiles. Accessed 6 June 2020.
[47] *bio.tools* Curators Guide. http://biotools.readthedocs.io/en/latest/curators_guide.html. Accessed 6 June 2020.
[48] Jiménez RC et al. Four simple recommendations to encourage best practices in research software. *F1000Research*, **6** (2017).

[49] Jagodnik KM et al. Developing a framework for digital objects in the Big Data to Knowledge (BD2K) commons: Report from the Commons Framework Pilots workshop. *Journal of Biomedical Informatics*, **71**, 49–57 (2017).
[50] Ison J, Ienasescu H, Rydza E, Chmura P, Rapacki K, Gaignard A, et al. Supporting data for "biotoolsSchema : a formalised schema for bioinformatics software description" GigaScience Database. 2020. http://dx.doi.org/10.5524/100840

Table 2. Software attributes

XML element/ JSON property	Description	Type	Cardinality
(Summary group)			
`name`	Canonical software name assigned by the software developer or service provider.	string	1 only
`description`	Textual description of the software.	string	1 only
`homepage`	Homepage of the software, or some URL that best serves this purpose.	URL	1 only
`biotoolsID`	Unique ID (case insensitive) of the tool that is assigned upon registration of the software in *bio.tools*, normally identical to tool name.	bio.tools tool ID	0 or 1
`biotoolsCURIE`	*bio.tools* CURIE (compact URI) based on the *bio.tools* tool ID.	URI	0 or 1
`version`	Version information (typically a version number) of the software applicable to this *bio.tools* entry.	string	0 or more
`otherID`	*A unique identifier of the software, typically assigned by an ID-assignment authority other than bio.tools.*		*0 or more*
`otherID->value`	Value of tool identifier.	string	1 only
`otherID->type`	Type of tool identifier.	enum	0 or 1
`otherID->version`	Version information (typically a version number) of the software applicable to this identifier.	string	0 or 1
(Labels group)			
`toolType`	A type of application software: a discrete software entity can have more than one type.	enum	0 or more
`topic`	General scientific domain the software serves or other general category.	EDAM Topic	0 or more
`operatingSystem`	The operating system that is supported by a downloadable software.	enum	0 or more
`language`	Name of programming language, *e.g.* used for the software source code or compatible with an API.	enum	0 or more
`license`	Software or data usage license.	enum	0 or 1
`collectionID`	Tag for a collection that the software has been assigned to within *bio.tools*.	string	0 or more
`maturity`	How mature the software product is.	enum	0 or 1
`cost`	Monetary cost of acquiring the software.	enum	0 or 1

accessibility	Whether there are non-monetary restrictions on accessing an online service.	enum	0 or 1
elixirPlatform	ELIXIR platform that is credited for developing or providing the software.	enum	0 or more
elixirNode	ELIXIR node that is credited for developing or providing the software.	enum	0 or more
elixirCommunity	Name of relevant ELIXIR (or associated) community.	enum	0 or more
function *(0 or more)*			
operation	The basic operation(s) performed by this software function.	EDAM Operation	1 or more
input|output	*Details of primary input / output.*		*0 or more*
input|output ->data	Type of primary input or output data.	EDAM Data	1 only
input|output ->format	Allowed format(s) of the input or output data (EDAM Format).	EDAM Format	0 or more
note	Concise comment about this function, if not apparent from the software description and EDAM annotations.	string	0 or 1
cmd	Relevant command, command-line fragment or option for executing this function / running the tool in this mode.	string	0 or 1
link *(0 or more)*			
url	A link of some relevance to the software.	URL	1 only
type	The type of data, information or system that is obtained when the link is resolved.	enum	1 or more
note	Comment about the link.	string	0 or 1
download *(0 or more)*			
url	Link to download (or repository providing a download) for the software.	URL	1 only
type	The type of data, information or system that is obtained when the link is resolved.	enum	1 only
note	Comment about the download.	string	0 or 1
version	Version information (typically a version number) of the software applicable to this download.	string	0 or 1
documentation *(0 or more)*			
url	Link to documentation on the web for the tool.	URL	1 only
type	Type of documentation that is linked to.	enum	1 or more
note	Comment about the documentation.	string	0 or 1
relation *(0 or more)*			
biotoolsID	*bio.tools* ID of an existing bio.tools entry to which this software is related.	bio.tools tool ID	1 only
type	Type of relation between this and another registered software.	enum	1 only
publication *(0 or more)*			
doi*	Digital Object Identifier of a publication about the software (* at least one of doi, pmid or pmcid must be specified).	doi	0 or 1*

`pmid*`	PubMed Identifier.	pmid	0 or 1*
`pmcid*`	PubMed Central Identifier.	pmcid	0 or 1*
`type`	Type of publication.	enum	0 or more
`version`	Software version information (typically number) applicable to this publication.	string	0 or 1
`note`	Comment about the publication.	string	0 or 1
`credit` *(0 or more)*			
`name*`	Name of the entity that is credited (* at least one of name, email or url must be specified).	string	0 or 1*
`email*`	Email address.	email address	0 or 1*
`url*`	URL, *e.g*. homepage of an institute.	URL	0 or 1*
`orcidid`	Unique identifier (ORCID iD) of an entity that is credited.	ORCID iD	0 or 1
`gridid`	Unique identifier (GRID ID) of an organisation that is credited.	GRID ID	0 or 1
`rorid`	Unique identifier (ROR ID) of an organisation that is credited.	ROR ID	0 or 1
`fundrefid`	Unique identifier (FundRef ID or Funder ID) of a funding organisation that is credited.	FundRef ID	0 or 1
`typeEntity`	Type of entity that is credited.	enum	0 or 1
`typeRole`	Role performed by the entity that is credited.	enum	0 or more
`note`	A comment about the credit.	string	0 or 1

Table 5. Comparison of *biotoolsSchema* and other software metadata initiatives

bio.tools	**CodeMeta / Schema.org 36/65**[4]	**OpenAIRE 18/23**	**DataCite 11/19**[5]	**Misc. RDF vocabs**	**HCLS 12/24**[6]	**eInfraCentral 23/24**[7]	**FORCE11 10/11**
`summary`							
`name`	name	Name	Title	rdfs:label, dct:title	Title	Service Name	Software name
`description`	description	Description	Description	rdfs:comment, dct:description	Description	Service Tagline, Service Description	Description
`homepage`	url	Landing page (datacite:alternateIdentifier)	-	foaf:page	HTML page	Service URL, Service Order	-
`biotoolsID`	identifier	Identifier	Identifier	dct:identifier		Service ID	Unique identifier
`biotoolsCURIE`	identifier	Identifier	Identifier	dct:identifier		(Service ID)	
`version`	softwareVersion	Version	Version	pav:version pav:hasCurrentVersion pav:previousVersion	Version identifier	Service Version	Version number

4 55 of which are Schema.org properties and 10 of which are specific to CodeMeta
5 disregarding DataCite subproperties
6 HCLS core metadata elements
7 disregarding service level targets and performance information (out of *biotoolsSchema* scope)

otherID	identifier	Alternate identifier	AlternateIdentifier, alternateIdentifierType	rdfs:seeAlso, dct:identifier	-	-	-
function							
operation	-	-	-	dcat:keyword	-	Service Tags	-
input|output ->data	-	-	-	dcat:keyword	-	-	-
input|output ->format	-	-	-	dcat:keyword	-	-	-
note	-	-	-	rdfs:comment	-	-	-
cmd	-	-	-	rdfs:comment	-	-	-
labels							
toolType	applicationSubCategory	Software Type	ResourceType	rdfs:comment		(Service Category, Service Subcategory)	
topic	keywords	Subject	Subject, valueURI	dcat:keywords	Keywords	Service Tags, Service Coverage	Keywords
operatingSystem	operatingSystem	-	-	dct:medium dct:mediator	-	-	-
language	programmingLanguage	Programming Language (datacite:format)	-	dct:language	Language	-	-
license	license	License Condition	Rights, rightsURI	dct:licence	License	-	Software license
collectionID	-	-	-	dct:identifier	-	-	-
maturity	-	-	-	-	-	Service Life Cycle Status[8]	-
cost	isAccessibleForFree[9]	-	-	-	-	Service Cost	-
link							
any type	relatedLink	-	-	-	-	-	-
”Repository”	codeRepository	Repository (datacite:publisher)	-	-	-	-	Location / repository
”Helpdesk”	-	-	-	-	-	Service Helpdesk, Service Feedback	-
”Mailing list”	-	-	-	-	-	Service Feedback	-
”Issue tracker”	codemeta:issueTracker	-	-	-	-	-	-
download							
any type	downloadUrl	-	-	dcat:downloadURL, prov:atLocation	-	-	-

[8] eInfraCentral uses a different but compatible controlled vocabulary
[9] True where cost==”Free of charge”

"Source code", "Software package" or "Binaries"	-	Distribution location (datacite:alternateIdentifier)	-	dcat:mediaType	-	-	-
"Icon"	-	-	-	-	Logo	Service Symbol	-
"Screenshot"	-	-	-	-	-	Service Multimedia	-
documentation							
"General"	codemeta:readme	Documentation (datacite:alternateIdentifier)	-	dcat:landingPage	Documentation	-	-
"User manual"	softwareHelp	-	-	-	-	Service User Manual	-
"Terms of use"	-	Access Rights (datacite:rights)	Rights, rightsURI	dct:rights	Rights	Service Terms of Use	-
"Training material"	-	-	-	-	-	Service Training Information	-
Publication							
doi|pmid|pmcid	referencePublication	-	-	dct:references	References	-	Index citations
credit							
name	givenName, familyName, affiliation, name	-	creatorName, givenName, familyName, affiliation,	foaf:name	-	[10],[11]	-
email	email	-	-	foaf:mbox	-	-	-
url	url	-	-	foaf:page prov:atLocation	-	-	-
orcidid	identifier	-	nameIdentifier	dct:identifier	-	-	-
typeEntity	-	-	nameType	-	-	-	-
typeRole	-	-	-	foaf:providedBy pav:createdBy pav:authoredBy pav:curatedBy pav:contributedBy	-	-	-
typeEntity=="Funding agency"	funder, codemetada:funding	Funding Reference	funderName, FundingReference	-	-	Service Funding	-
typeRole=="Developer"	author, creator	Author	Creator	dct:creator	Creators	-	Author(s)
typeRole=="Contributor"	contributor, editor	-	Contributor	-	-	-	Contributor role
typeRole=="Provider"	provider, producer	-	-	-	-	Service Provider Name	-
typeRole=="Maintainer"	codemeta:maintainer	-	-	-	-	-	-
typeRole=="Primary contact"	-	Contact Person , Contact Group	-	dct:contributor	Contributors	-	-

(1) These fields are compatible with EDAM vocabulary.

[10] with typeEntity=="Institute" and typeRole=="Provider"

[11] where typeEntity=="Funding agency"

Table 6. Role of bio.tools and biotoolsSchema in the evaluation of software FAIRness

	FAIR principle for software	Provided by *bio.tools*	Provided by *biotoolsSchema*
F1	Software and its associated metadata have a global, unique and persistent identifier for each released version.	*bio.tools* assigns persistent and unique identifiers to registered software.	Attributes for *bio.tools*-specific software identifiers ("biotoolsID" and "biotoolsCURIE") and other identifiers ("otherID") including doi, rrid and cpe.
F2	Software is described with rich metadata.		*biotoolsSchema* defines over 50 important scientific, technical and administrative attributes that support cataloguing, discovery, use and interoperability of software.
F3	Metadata clearly and explicitly include identifiers for all the versions of the software it describes.		*biotoolsSchema* supports the annotation of all versions of the software applicable to a bio.tools entry. Version information can also be attached to specific identifier, download or publication attributes.,
F4	Software and its associated metadata are included in a searchable software registry.	*bio.tools* information includes all metadata supported by *biotoolsSchema*.	biotoolSchema supports links to where software source code and binaries may be downloaded.
A1	Software and its associated metadata are accessible by their identifier using a standardized communications protocol.	Metadata can be retrieved from *bio.tools* using an API.	
A1.1	The protocol is open, free, and universally implementable.	bio.tools API is a fully documented REST API (see https://biotools.readthedocs.io/en/latest/api_reference.html).	
A1.2	The protocol allows for an authentication and authorization procedure, where necessary.	Authentication and authorizations management are handled by the REST API (see *e.g.* https://biotools.readthedocs.io/en/latest/api_reference.html#editing-permissions).	
A2	Software metadata are accessible, even when the software is no longer available.	Curation practice for *bio.tools* is to set the maturity to "legacy" instead of removing an entry; entries are never deleted.	*biotoolsSchema* supports annotation of software as "legacy" ("maturity" attribute).
I1	Software and its associated metadata use a formal, accessible, shared and broadly applicable language to facilitate machine readability and data exchange.	Schema.org semantic markup is available through the API.	*biotoolsSchema* is specified as both XSD and JSON Schema, and is compatible with Schema.org.
I2S.1	Software and its associated metadata are formally described using controlled		*biotoolsSchema* makes extensive use of controlled vocabularies, all of which are rendered FAIR through ontology portals

	vocabularies that follow the FAIR principles.		such as OLS, or are publicly available and documented (see Table 3).
I2S.2	Software use and produce data in types and formats that are formally described using controlled vocabularies that follow the FAIR principles.		*biotoolsSchema* supports the data consumed and produced by software to be described using the EDAM ontology.
I4S	Software dependencies are documented and mechanisms to access them exist.		*biotoolsSchema* provides a controlled vocabulary of documentation types (*e.g.* Installation instructions) which should describe software dependencies in detail.<br><br>It also provides a controlled vocabulary for describing dependencies between software resources as relationships between tools (using *e.g.* "uses" and "usedBy" of ”relation” attribute).
R1.1	Software and its associated metadata have independent, clear and accessible usage licenses compatible with the software dependencies.	*bio.tools* entries are available under CC-BY-4.0 license.	*biotoolsSchema* itself is licensed under CC-BY-SA 4.0.<br>Individual software licenses are documented by the "license" attribute.
R1.2	Software metadata include detailed provenance, detail level should be community agreed.		*biotoolsSchema* supports links to the software repository where provenance info such as version history, releases, contributors *etc.* should be hosted.<br><br>*biotoolsSchema* also includes a detailed credit model, which provides contact details for various types of contributing entities (*e.g.* Person, Institute) and roles (*e.g.* Developer, Maintainer).
R1.3	Software metadata and documentation meet domain-relevant community standards.		*biotoolsSchema* attribute "documentation" supports links to various documentation resources, and specification of documentation type (e.g. “Citation instructions”).

Figure 1

Click here to access/download
Supplementary Material
Figure1.svg

Figure 2

Click here to access/download

Supplementary Material

Figure2.svg

Figure 3

Click here to access/download
Supplementary Material
Figure3.svg

Cover letter
Click here to access/download;Personal Cover;Cover letter Ison et al.pdf

RE: Submission of manuscript "biotoolsSchema : a formalised schema for bioinformatics software description" for consideration of publication in *GigaScience*

Submission Date: July 6th, 2020

Dear GigaScience Editors,

The use of software is ubiquitous in the biological and biomedical sciences, but the field has lacked a uniform way for developers to describe their tools and help researchers understand, compare and utilise all the diverse software that is available. Our manuscript summarises the results of an effort - spanning 8 years - in producing a comprehensive, general-purpose schema (biotoolsSchema) that enables a broad range of tools to be described in a concise, precise and consistent way. biotoolsSchema provides both a syntax and (by leveraging the EDAM ontology) the semantics for life science research software. It is used in (and has co-evolved with) the *bio.tools* registry for the description of 17K+ tools provided by 2.4K contributors. We hope that by publishing a technical description of the schema in GigaScience, we can bring the work to the attention of a bigger audience - to find new applications beyond *bio.tools*, to spare costly reinvention of the functions provided by biotoolsSchema, and more broadly, to promote the provision of high quality software metadata in general - to benefit the whole life sciences globally.

We would like to highlight that biotoolsSchema is fully compatible with, but serves quite a different purpose to the generic *schema.org*. The latter provides a set of fields for mark-up of web pages (see for example https://developers.google.com/search/docs/data-types/software-app), primarily to support "rich results" in web browsers such as Google. In contrast, biotoolsSchema provides a syntax (including regular expressions) and semantics (EDAM + 18 further controlled vocabularies) for over 50 key scientific, technical and administrative attributes. It is thus suited both as an exchange format and for applications in software discovery and interoperability. For example, the schema (or parts of it) can be used for software descriptions in repositories such as GitHub, and re-used downstream in various settings. To put biotoolsSchema in context and to help developers, we include in the article a technical comparison to related initiatives, including a metadata crosswalk that has been submitted to CodeMeta.

On behalf of the authors, I declare there are no competing interests, that all authors have approved the manuscript for submission, and that the content of the manuscript has not been published, or submitted for publication elsewhere. I was not certain which of the GigaScience article types is most appropriate - probably *Technical Note* - so I have prepared the text and sections in a manner which I think best conveys the narrative of the article.

I would like to suggest the following reviewers, who the authors do not have close ties with, but I think would give the article a fair hearing:

Brian O'Connor (boconnor@broadinstitute.org)
Egon Willighagen (egon.willighagen@gmail.com)
Tim Griffin (tgriffin@umn.edu)

With best regards

Jon Ison

jon.c.ison@gmail.com

Reponse to Editor & Reviewers
Click here to access/download;Personal Cover;biotoolsSchema paper _ response to reviewers.docx

Dear Dr Zauner,

On behalf of the authors I would like to thank you, and the reviewers for their very thorough treatment of our manuscript. They raise many relevant points, and we address each of these in detail in our point-by-point response, indicating changes made to the manuscript. We have also structured the abstract into sections and included ORCIDs for authors for whom these are available.

As for the key points you highlight - evidence that biotoolsSchema supports FAIR principles, and issues around immutability, persistence and the minimal mandatory core of metadata, we address these rigorously in our responses (points 1.8, 1.9, 1.10 and 1.17 below). Immutability and persistence of software metadata is delivered by bio.tools, through its ID scheme and Tool Cards (point 1.10, Table 6, plus revised text in the section "Implementation of biotoolsSchema in bio.tools"). The justification for the minimal mandatory core of metadata - which is a practical necessity for building a large-scale registry such as bio.tools based on biotoolsSchema, is explained in points 1.8 and 1.9 (with revisions to the text in “Software attributes”). We have included in the manuscript a new Table 6 which summarises how bio.tools and biotoolsSchema support each of the FAIR principles (points 1.10 and 1.17) as enumerated in https://www.force11.org/group/fairgroup/fairprinciples, and subsequently mapped to the software space in https://content.iospress.com/articles/data-science/ds190026. We intend (in a future work) to go further, and produce a set of objective and transparent metrics of FAIRness, based on biotoolsSchema attributes, and calculate these for all bio.tools entries using a new Tool Information Profile system (https://github.com/bio-tools/Tool-Information-Profiles) that is being developed for this purpose.

With best regards

Jon

Dr Jon Ison
jon.c.ison@gmail.com

Response to reviewer #1

1.1 "What is the justification for 50 attributes?"

The 50 attributes in the schema are simply what we ended up with after many iterations of development and releases over the years of biotoolsSchema development - a major driver of this effort being community workshops and (crucially) engaging with and incorporating the requirements of bio.tools content providers and end-users, with respect to what information people find valuable and are prepared to provide.

1.2 "It is unclear if or how this list is extensible over time. If this standard can evolve, why state 50, and if it cannot, what assurances are there that it will continue to be an effective descriptive schema in the future?"

The list of attributes certainly is extensible over time and biotoolsSchema was designed openly, in a community-based process that was described in https://academic.oup.com/bib/article/21/5/1697/5560007 (current version is 3.3.0), and is licensed for this purpose; reuse and contributions are welcome. New official releases will incorporate end-user requirements, changes can be requested through collaboration with the authors, GitHub issues etc.

Version 3.3.0 is the ninth in a series of public releases, and in each of them the attributes list was revised, to reflect the needs of our users. This latest list of 50 attributes is not cast in stone, but rather is the current status of the schema, and it might (and will probably) evolve in the future as new requirements emerge. We have added a sentence to the section "Development process and status" clarifying the above.

1.3 "There are instances where this list already seems restrictive, such as "accessibility" in Table 3 which appears to support any of three options, though it is easy to imagine many more distinct types of access control, for instance."

Indeed, and should a compelling use-case arise, the "accessibility" options can easily be extended (all such controlled vocabularies are defined as simple enumerations of terms in the schema). In practice, one has to strike a balance between what attributes reasonably capture salient details, and what is realistic to curate and useful to end-users. The rigorous semantics and syntax of biotoolsSchema has advantages over approaches such as the use of folksonomies, which are very flexible, but can be less tractable in the context of registries such as bio.tools. Where possible, biotoolsSchema re-uses well established controlled vocabularies which are maintained independently, such as SPDX for software license.

1.4 "The relationship between CWL or other execution standards and biotoolsSchema is presently unclear. Can a CWL definition be included/referenced within a biotoolsSchema?"

biotoolsSchema is a metadata format that provides a description of software tools and services to address its findability, but not its execution, whereas CWL Tools, Galaxy Tools, and other execution formats allow the execution of tools in workflow environments but do not enable an exhaustive description of the resources. Acknowledging this difference, biotoolsSchema allows adding links to execution formats (see for instance the link to the CWL wrappers from the yara bio.tools entry https://bio.tools/yara), and reversely some links to bio.tools entries can be added to CWL wrappers (see for instance https://github.com/common-workflow-library/bio-cwl-tools/blob/release/qualimap/qualimap_rnaseq.cwl#L21) and Galaxy wrappers (see for instance https://github.com/galaxyproject/tools-iuc/blob/master/tools/circos/circos.xml#L5).

1.5 "Similarly, what is the line between an execution standard and what is included in biotoolsSchema? For instance, it appears as though inputs and outputs are defined here, which is a considerable portion of the execution standard. This apparent duplication of content between tool descriptions gives rise to the possibility of inconsistency between them. If an execution record is referenced, are there validators which could be used to ensure consistency of duplicated information across both records?"

The relationship and degree of overlap between registry-focused formats such as biotoolsSchema have been explored in previous work (cite "Using registries to integrate bioinformatics tools and services into workbench environments", doi:10.1007/s10009-015-0392-z), and used to help the generation of execution formats (cite "Using bio. tools to generate and annotate workbench tool descriptions", doi:10.12688/f1000research.12974.1). Future developments based on these works will probably be focused on improving such tooling to resolve inconsistencies between such formats and cross-validating the different descriptions. We expanded slightly the text of the "Discussion" section to cite and summarise this work.

1.6 "Does this schema/manuscript propose a mechanism for storage or access of these records aside from the bio.tools website?"

The biotoolsSchema format itself is not, in its essence, restricted to usage within bio.tools. One of the current efforts led by the ELIXIR Europe organization is the creation of a github-based platform to store and exchange openly software tool metadata between multiple resources within ELIXIR (e.g. bio.tools tools registry, BioContainers containers registry, OpenEBench benchmarking and monitoring platform, usegalaxy.eu portal) and beyond it (BioConda, Debian Med, etc.). This platform, by allowing the different resources to push their data and pull other data, will facilitate the cross-linking of their records and cross-consolidation of their metadata. Eventually, we aim at allowing the maintenance of tool metadata as biotoolsSchema files in their source repositories which

will be automatically synchronized with this platform. We plan to publish a description of this emerging platform once it is more mature.

1.7 "What assurance that this website and service will persist beyond a funding cycle, for instance? (i.e. Is it supported by a large public group organization? Could it rely upon such a service, e.g. Zenodo?). If this is not addressed, the records would not live up to the FAIR requirement of persistency and immutability."
bio.tools is supported by ELIXIR Europe, and is one of the commissioned services (https://elixir-europe.org/about-us/commissioned-services/registry-tools) of this organization. As such, not only do bio.tools and biotoolsSchema involve the work of multiple national groups (e.g., in Denmark, France and Norway), but they are funded and evaluated with the specific goals of ensuring their long term availability and sustainability. Reviewer 2 also raised a comment about the sustainability; we have added a short paragraph (in “Development process and status”) to describe how biotoolsSchema development is (through its anchoring within the ELIXIR infrastructure) sustainable.

1.8 “Please discuss the justification for making such a large majority of the fields optional. If the intent is to truly have richly described and queryable tools, the bar for flexibility appears to currently be set too low for this goal to be reached, also limiting the strength of the claim that the metadata is "high quality".”
The core of mandatory attributes is indeed intentionally small, having been whittled down during the evolution of the project, and was found to be a necessity for the curation of tools as such large scale. The primary reason was to encourage (by settling an easily achievable goal) new contributions, and also to facilitate contributions from institutes, projects etc. who wanted to deposit a large number of tools with basic descriptions in a first pass, and then subsequently improve those descriptions. Even a basic entry goes a long way to making a tool more FAIR, for reasons now summarised in Table 6. A secondary reason is that biotoolsSchema has a very broad scope in terms of the types of resources that it can be used to describe; not all attributes are applicable to all types of tool, furthermore, not all attributes are available from all contributors. The fact there are many very rich descriptions (see e.g. http://proteomics.bio.tools/) is evidence that a low bar for the number of mandatory attributes certainly does not, in itself, preclude high quality. Internally, we do track information richness using our “Tool Information Standards” system, which describes what attributes should be provided at various tiers of detail and quality. This system is summarised in https://academic.oup.com/bib/article/21/5/1697/5560007.

1.9 “The current flexibility may have serious consequences on the consumption of described tools, such as in the case where licensing information is not provided or known by consumers. While the schema supports the FAIR curation of tools when well implemented, the usefulness of this schema is severely limited if the minimum specification does not.”
We are well aware that the availability of data can be a serious issue. For exactly the reason pointed out by the reviewer, we have been developing the “Tool Information Standards” system (described in https://academic.oup.com/bib/article/21/5/1697/5560007) used internally in bio.tools into a more flexible, robust and independent service. Progress on this is available at https://github.com/bio-tools/Tool-Information-Profiles. Tool Information Profiles will, in due course, replace the current “Tool Information Standards” system. In short, a tool information profile specifies which tool attributes (defined in biotoolsSchema) must, should or may be specified for different types of tools within a set of tool descriptions. It thus augments (and ameliorates the limitations of) the small mandatory core attributes defined by biotoolsSchema, by allowing to adapt these requirements to project or community-specific requirements. A practical application will be to use such profiles for filtering, or targeted improvement of sets of tool descriptions, before consumption by other systems. We are hoping to publish this work in due course.

1.10 "It would be valuable to query existing descriptions in bio.tools and see what portion of them meet the standard of being FAIR. This analysis could be included as a sample use-case showcasing the value of biotoolsSchema, as well, and provide further justification and clarification around its adoption."
We do agree on the value of evaluating FAIRness of software tools, however, in practice this is non-trivial owing to the numerous and complex indicators and metrics corresponding to FAIRness, that have been subject to much debate. To make a pragmatic start, we examined the criteria defined in “Towards FAIR principles for research software" (https://content.iospress.com/articles/data-science/ds190026), with respect to the features of bio.tools and biotoolsSchema, and specifically their impact on the FAIRness of a tool. The results of this comparison have been added to the manuscript as Table 6.
We would like, and intend to go further, but this has to be done with great care, given the obvious sensitivities of the implied assignation of some tools as FAIR, and some not, and especially because we would be evaluating FAIRness on the metadata we have in bio.tools (tool authors should have the possibility to improve their entries before we evaluate them). We envisage developing a Tool Information Profile (as previously mentioned) for FAIRness, and use it to provide an open, transparent and flexible framework to evaluate FAIRness of all tools in bio.tools, using biotoolsSchema data. This requires community agreement on an exact set of metrics (which should be objective and transparent) for its evaluation. While the indicators in “Towards FAIR principles for research software" are an excellent starting point, these are by no means the only set of metrics. We will therefore, in due course, run a community event to explore these metrics and advance this work. We hope that this is, for now, an adequate response.

1.11 "Much of the Comparison to related efforts section reads more like a list than flowing text. Please add supporting text to make this read more naturally, and situate biotoolsSchema explicitly relative to these efforts, emphasizing novel elements."
We have revised this section extensively along the lines suggested. It would be possible to write an entire article which compares and contrasts the various approaches, historical and contemporary, which exist in this space, so we hope our revision will suffice. We include in the revision various new relevant developments around bio.tools and biotoolSchema.

1.12 "The mention of tasks at the beginning of the manuscript is not mentioned or referenced later on once the schema has been presented. The efficacy for this schema at accomplishing each task should be explicitly mentioned as its features or attributes are introduced and discussed."
This is a good point, and an omission on our side. We have revised the Discussion accordingly, to refer back to the tasks mentioned in the Introduction.

1.13 "The explicit comparison of features or interfaces between tools is an excellent feature, and I think it should be more prominently mentioned."
We added a sentence in the "Background" section that emphasizes this feature (provision of a model for the description of tool functions), and also modified the text in the Discussion to mention that advanced possibilities such as workflow composition or provenance tracking are mostly enabled by this original feature.

1.14 "Another description standard (specifically, an execution standard for tools much like CWL) which closely aligns itself with enabling the FAIR principles is Boutiques (https://boutiques.github.io); consider referencing this standard, and in particular, the tooling it provides to facilitate fair curation of records (more details here: https://figshare.com/articles/poster/fair-pipelines-poster_pdf/8143241)."
We thank the reviewer for pointing us to this relevant work we were not aware of. We have indeed added it , and its contribution to software FAIRness, to the related work we refer to in the paper.

1.15 "The design considerations section provides a list that could be of extreme value to tool and standard developers. Could this be provided as an independent resource or checklist that is made more widely available?"
We thank the reviewer for his interest in this content. Following his suggestion, we added it to the public documentation of biotoolsSchema, it can be found at https://biotoolsschema.readthedocs.io/en/latest/design_considerations.html.

1.16 "The scope for biotoolsSchema is unclear for the majority of the manuscript, and should be placed closer to the beginning. In particular, the relationship or relative objectives with this and execution standards such as CWL or bioinformatics ontologies such as EDAM."
We have moved the complete "Scope" subsection to the "Findings" section, where we believe it helps get a better overview of biotoolsSchema. A detailed comparison of the relationship and relative objectives of "registry-focused" (e.g. biotoolsSchema) and "execution-focused" (e.g. CWL) tool descriptions was published in a previous paper (https://link.springer.com/article/10.1007/s10009-015-0392-z), which we now cite in this article.

1.17 "FAIR terms should be added to the table which compares ontologies (in particular, the 15 as enumerated here: https://www.force11.org/group/fairgroup/fairprinciples), possibly in the Force11 column."
The FAIR principles listed at this URL were mapped and analysed with respect to software in the article "Towards FAIR principles for research software" (https://content.iospress.com/articles/data-science/ds190026). We have in turn added to the article (in new Table 6) a summary of how bio.tools and biotoolsSchema supports each of these principles. The detailed mapping of indicators of FAIRness to biotoolsSchema attributes, with respect to producing a set of objective and transparent metrics of FAIRness will be the subject of a future work using the Tool Information Profile system, as outlined at length in a previous point (see point 1.10).

1.18 "Text in the legends, as well as parts of the figure itself, for figures 1, 2, and 3 is unreadable."
We have provided larger / higher resolution versions of Figures 1, 2 and 3 which are more readable.

Response to reviewer #2

2.1 "All schemas benefit greatly from being community driven, and the authors do note that extensive community consultation has been undertaken to arrive at a community consensus as to the content of the schema, but provide few details of the mechanism that was employed that has led to the consensus. I would recommend that inclusion of this information is critical to illustrate that the schema is indeed community agreed, and a summary describing who, what and how the community consensus was reached would be beneficial (e.g. details of workshops, working group membership etc), as any governance type arrangements that have led to each version being agreed / 'signed off'"

We thank Reviewer 2 for his interest in this important aspect of our work. As mentioned in other places in this letter, the overall work of community development of biotoolsSchema is part of a wider process also involving the development of the bio.tools registry, the EDAM ontology and other components of the ELIXIR Tools Platform, as outlined in a recent publication (cite https://academic.oup.com/bib/article/21/5/1697/5560007). The schema development has been in context of major European infrastructure projects (BioMedBridges, ELIXIR EXCELERATE) and ELIXIR national node infrastructures and has leveraged their governance structures, e.g. ELIXIR EXCELERATE WP1 partners. The current governance structure can be seen at https://biotoolsschema.readthedocs.io/en/latest/contributors.html. Over the years biotoolsSchema development has featured at many hackathons, meetings and workshops, using agile methods (e.g. feature poker, sprints etc.) with open participation (within and beyond ELIXIR). It would be too verbose to describe in detail all of this (which are summarised at https://biotools.readthedocs.io/en/latest/events.html), so we have added a short summary on the governance, and how we arrived at a community consensus to the section "Development process and status" of the manuscript, and hope this will suffice.

2.2 "The authors state that "future changes will be pragmatic, driven by community-use cases". The paper would benefit from inclusion of clear guidelines or instructions on how the wider community can provide feedback which may influence the future development of the schema - ie. how to provide feedback on v3.3.0 and how to get involved in influencing any future versions."

The mechanisms of community engagement around biotoolsSchema (and other technologies in its orbit) have been mentioned in https://academic.oup.com/bib/article/21/5/1697/5560007. We have updated the text (in section "Development process and status") to provide a short summary and refer to the paper mentioned. We have also added contribution guidelines to the online docs (https://biotoolsschema.readthedocs.io/en/latest/what_is_biotoolsschema.html#how-to-contribute-to-biotoolsschema) and link to these from a new CONTRIBUTING.md file (hhttps://github.com/bio-tools/biotoolsSchema/blob/master/CONTRIBUTING.md) in biotoolsSchema repo.

2.3 "Some commentary on the potential sustainability of the schema would be useful - is its use recommended, mandated or required by any groups? The authors briefly discuss the involvement of the ELIXIR consortium in its development, and I note that three controlled vocabularies exist for ELIXIR platform, community and node, so I am guessing that its use is at least recommended by ELIXIR. Some clarification around the use of the schema in ELIXIR and any other other efforts would be valuable to help illustrate how widespread adoption is / is likely to be moving forward."

Reviewer 1 also raised a comment (see point 1.7) about the sustainability; we have added a short paragraph (in the section "Development process and status") to describe how biotoolsSchema development is (through it's anchoring within the ELIXIR infrastructure) sustainable including a note about its current and likely future adoption. In short, bio.tools is supported by ELIXIR Europe, and is one of the commissioned services (https://elixir-europe.org/about-us/commissioned-services/registry-tools) of this organization. As such, not only do bio.tools and biotoolsSchema involve the work of multiple national groups (e.g., in Denmark, France and Norway), but they are funded and evaluated with the specific goals of ensuring their long term availability and sustainability.

2.4 "Table 3 states that there are 16 controlled vocabularies, however 18 are listed and 18 are included in the online documentation https://biotoolsschema.readthedocs.io/en/latest/controlled_vocabularies.html."

This error has been corrected.

2.5 "Figures 2 and 3 are quite small / low resolution - these would benefit from being larger / higher resolution."

We have included larger versions of Figures 2 and 3.

2.6 "Similar to Figure 2, Figure 3 should also include a note that the illustration example is for the ProCon tool."

We have included the note as suggested.
