# Supplementary material for: biotoolsSchema: a formalized schema for bioinformatics software description
Source: Gigascience. 2021 Jan 27;10(1):giaa157. doi: 10.1093/gigascience/giaa157 (PMC7842104; doi:10.1093/gigascience/giaa157)
Supplement: giaa157_Response_to_Reviewer_Comments_Original_Submission [file giaa157_response_to_reviewer_comments_original_submission.pdf]

Author's Response To Reviewer Comments

Close

Please see file "biotoolsSchema paper _ response to reviewers.docx" attached during submission. Included verbatim beow.

Dear Dr Zauner,

On behalf of the authors I would like to thank you, and the reviewers for their very thorough treatment of our manuscript. They raise many relevant points, and we address each of these in detail in our point-by-point response, indicating changes made to the manuscript. We have also structured the abstract into sections and included ORCIDs for authors for whom these are available.

As for the key points you highlight - evidence that biotoolsSchema supports FAIR principles, and issues around immutability, persistence and the minimal mandatory core of metadata, we address these rigorously in our responses (points 1.8, 1.9, 1.10 and 1.17 below). Immutability and persistence of software metadata is delivered by bio.tools, through its ID scheme and Tool Cards (point 1.10, Table 6, plus revised text in the section "Implementation of biotoolsSchema in bio.tools"). The justification for the minimal mandatory core of metadata - which is a practical necessity for building a large-scale registry such as bio.tools based on biotoolsSchema, is explained in points 1.8 and 1.9 (with revisions to the text in “Software attributes”). We have included in the manuscript a new Table 6 which summarises how bio.tools and biotoolsSchema support each of the FAIR principles (points 1.10 and 1.17) as enumerated in https://www.force11.org/group/fairgroup/fairprinciples, and subsequently mapped to the software space in https://content.iospress.com/articles/data-science/ds190026. We intend (in a future work) to go further, and produce a set of objective and transparent metrics of FAIRness, based on biotoolsSchema attributes, and calculate these for all bio.tools entries using a new Tool Information Profile system (https://github.com/bio-tools/Tool-Information-Profiles) that is being developed for this purpose.

With best regards

Jon

Dr Jon Ison
jon.c.ison@gmail.com

Response to reviewer #1

1.1 “What is the justification for 50 attributes?”
The 50 attributes in the schema are simply what we ended up with after many iterations of development and releases over the years of biotoolsSchema development - a major driver of this effort being community workshops and (crucially) engaging with and incorporating the requirements of bio.tools content providers and end-users, with respect to what information people find valuable and are prepared to provide.

1.2 “It is unclear if or how this list is extensible over time. If this standard can evolve, why state 50, and if it cannot, what assurances are there that it will continue to be an effective descriptive schema in the future?”
The list of attributes certainly is extensible over time and biotoolsSchema was designed openly, in a community-based process that was described in https://academic.oup.com/bib/article/21/5/1697/5560007 (current version is 3.3.0), and is licensed for this purpose; reuse and contributions are welcome. New official releases will incorporate end-user requirements, changes can be requested through collaboration with the authors, GitHub issues etc. Version 3.3.0 is the ninth in a series of public releases, and in each of them the attributes list was

revised, to reflect the needs of our users. This latest list of 50 attributes is not cast in stone, but rather is the current status of the schema, and it might (and will probably) evolve in the future as new requirements emerge. We have added a sentence to the section “Development process and status“ clarifying the above.

1.3 “There are instances where this list already seems restrictive, such as "accessibility" in Table 3 which appears to support any of three options, though it is easy to imagine many more distinct types of access control, for instance.”
Indeed, and should a compelling use-case arise, the “accessibility” options can easily be extended (all such controlled vocabularies are defined as simple enumerations of terms in the schema). In practice, one has to strike a balance between what attributes reasonably capture salient details, and what is realistic to curate and useful to end-users. The rigorous semantics and syntax of biotoolsSchema has advantages over approaches such as the use of folksonomies, which are very flexible, but can be less tractable in the context of registries such as bio.tools. Where possible, biotoolsSchema re-uses well established controlled vocabularies which are maintained independently, such as SPDX for software license.

1.4 “The relationship between CWL or other execution standards and biotoolsSchema is presently unclear. Can a CWL definition be included/referenced within a biotoolsSchema?”
biotoolsSchema is a metadata format that provides a description of software tools and services to address its findability, but not its execution, whereas CWL Tools, Galaxy Tools, and other execution formats allow the execution of tools in workflow environments but do not enable an exhaustive description of the resources. Acknowledging this difference, biotoolsSchema allows adding links to execution formats (see for instance the link to the CWL wrappers from the yara bio.tools entry https://bio.tools/yara), and reversely some links to bio.tools entries can be added to CWL wrappers (see for instance https://github.com/common-workflow-library/bio-cwl-tools/blob/release/qualimap/qualimap_rnaseq.cwl#L21) and Galaxy wrappers (see for instance https://github.com/galaxyproject/tools-iuc/blob/master/tools/circos/circos.xml#L5).

1.5 “Similarly, what is the line between an execution standard and what is included in biotoolsSchema? For instance, it appears as though inputs and outputs are defined here, which is a considerable portion of the execution standard. This apparent duplication of content between tool descriptions gives rise to the possibility of inconsistency between them. If an execution record is referenced, are there validators which could be used to ensure consistency of duplicated information across both records?”
The relationship and degree of overlap between registry-focused formats such as biotoolsSchema have been explored in previous work (cite "Using registries to integrate bioinformatics tools and services into workbench environments", doi:10.1007/s10009-015-0392-z), and used to help the generation of execution formats (cite "Using bio. tools to generate and annotate workbench tool descriptions", doi:10.12688/f1000research.12974.1). Future developments based on these works will probably be focused on improving such tooling to resolve inconsistencies between such formats and cross-validating the different descriptions. We expanded slightly the text of the "Discussion" section to cite and summarise this work.

1.6 "Does this schema/manuscript propose a mechanism for storage or access of these records aside from the bio.tools website?"
The biotoolsSchema format itself is not, in its essence, restricted to usage within bio.tools. One of the current efforts led by the ELIXIR Europe organization is the creation of a github-based platform to store and exchange openly software tool metadata between multiple resources within ELIXIR (e.g. bio.tools tools registry, BioContainers containers registry, OpenEBench benchmarking and monitoring platform, usegalaxy.eu portal) and beyond it (BioConda, Debian Med, etc.). This platform, by allowing the different resources to push their data and pull other data, will facilitate the cross-linking of their records and cross-consolidation of their metadata. Eventually, we aim at allowing the maintenance of tool metadata as biotoolsSchema files in their source repositories which will be automatically synchronized with this platform. We plan to publish a description of this emerging platform once it is more mature.

1.7 "What assurance that this website and service will persist beyond a funding cycle, for instance? (i.e. Is it supported by a large public group organization? Could it rely upon such a service, e.g. Zenodo?). If this is not addressed, the records would not live up to the FAIR requirement of persistency and immutability."
bio.tools is supported by ELIXIR Europe, and is one of the commissioned services (https://elixir-europe.org/about-us/commissioned-services/registry-tools) of this organization. As such, not only do bio.tools and biotoolsSchema involve the work of multiple national groups (e.g., in Denmark, France and

Norway), but they are funded and evaluated with the specific goals of ensuring their long term availability and sustainability. Reviewer 2 also raised a comment about the sustainability; we have added a short paragraph (in "Development process and status") to describe how biotoolsSchema development is (through its anchoring within the ELIXIR infrastructure) sustainable.

1.8 "Please discuss the justification for making such a large majority of the fields optional. If the intent is to truly have richly described and queryable tools, the bar for flexibility appears to currently be set too low for this goal to be reached, also limiting the strength of the claim that the metadata is "high quality"."
The core of mandatory attributes is indeed intentionally small, having been whittled down during the evolution of the project, and was found to be a necessity for the curation of tools as such large scale. The primary reason was to encourage (by settling an easily achievable goal) new contributions, and also to facilitate contributions from institutes, projects etc. who wanted to deposit a large number of tools with basic descriptions in a first pass, and then subsequently improve those descriptions. Even a basic entry goes a long way to making a tool more FAIR, for reasons now summarised in Table 6. A secondary reason is that biotoolsSchema has a very broad scope in terms of the types of resources that it can be used to describe; not all attributes are applicable to all types of tool, furthermore, not all attributes are available from all contributors. The fact there are many very rich descriptions (see e.g. http://proteomics.bio.tools/) is evidence that a low bar for the number of mandatory attributes certainly does not, in itself, preclude high quality. Internally, we do track information richness using our "Tool Information Standards" system, which describes what attributes should be provided at various tiers of detail and quality. This system is summarised in https://academic.oup.com/bib/article/21/5/1697/5560007.

1.9 "The current flexibility may have serious consequences on the consumption of described tools, such as in the case where licensing information is not provided or known by consumers. While the schema supports the FAIR curation of tools when well implemented, the usefulness of this schema is severely limited if the minimum specification does not."
We are well aware that the availability of data can be a serious issue. For exactly the reason pointed out by the reviewer, we have been developing the "Tool Information Standards" system (described in https://academic.oup.com/bib/article/21/5/1697/5560007) used internally in bio.tools into a more flexible, robust and independent service. Progress on this is available at https://github.com/bio-tools/Tool-Information-Profiles. Tool Information Profiles will, in due course, replace the current "Tool Information Standards" system. In short, a tool information profile specifies which tool attributes (defined in biotoolsSchema) must, should or may be specified for different types of tools within a set of tool descriptions. It thus augments (and ameliorates the limitations of) the small mandatory core attributes defined by biotoolsSchema, by allowing to adapt these requirements to project or community-specific requirements. A practical application will be to use such profiles for filtering, or targeted improvement of sets of tool descriptions, before consumption by other systems. We are hoping to publish this work in due course.

1.10 "It would be valuable to query existing descriptions in bio.tools and see what portion of them meet the standard of being FAIR. This analysis could be included as a sample use-case showcasing the value of biotoolsSchema, as well, and provide further justification and clarification around its adoption."
We do agree on the value of evaluating FAIRness of software tools, however, in practice this is non-trivial owing to the numerous and complex indicators and metrics corresponding to FAIRness, that have been subject to much debate. To make a pragmatic start, we examined the criteria defined in "Towards FAIR principles for research software" (https://content.iospress.com/articles/data-science/ds190026), with respect to the features of bio.tools and biotoolsSchema, and specifically their impact on the FAIRness of a tool. The results of this comparison have been added to the manuscript as Table 6.
We would like, and intend to go further, but this has to be done with great care, given the obvious sensitivities of the implied assignation of some tools as FAIR, and some not, and especially because we would be evaluating FAIRness on the metadata we have in bio.tools (tool authors should have the possibility to improve their entries before we evaluate them). We envisage developing a Tool Information Profile (as previously mentioned) for FAIRness, and use it to provide an open, transparent and flexible framework to evaluate FAIRness of all tools in bio.tools, using biotoolsSchema data. This requires community agreement on an exact set of metrics (which should be objective and transparent) for its evaluation. While the indicators in "Towards FAIR principles for research software" are an excellent starting point, these are by no means the only set of metrics. We will therefore, in due course, run a community event to explore these metrics and advance this work. We hope that this is, for now, an adequate response.

1.11 “Much of the Comparison to related efforts section reads more like a list than flowing text. Please add supporting text to make this read more naturally, and situate biotoolsSchema explicitly relative to these efforts, emphasizing novel elements.”
We have revised this section extensively along the lines suggested. It would be possible to write an entire article which compares and contrasts the various approaches, historical and contemporary, which exist in this space, so we hope our revision will suffice. We include in the revision various new relevant developments around bio.tools and biotoolSchema.

1.12 “The mention of tasks at the beginning of the manuscript is not mentioned or referenced later on once the schema has been presented. The efficacy for this schema at accomplishing each task should be explicitly mentioned as its features or attributes are introduced and discussed.”
This is a good point, and an omission on our side. We have revised the Discussion accordingly, to refer back to the tasks mentioned in the Introduction.

1.13 “The explicit comparison of features or interfaces between tools is an excellent feature, and I think it should be more prominently mentioned.”
We added a sentence in the "Background" section that emphasizes this feature (provision of a model for the description of tool functions), and also modified the text in the Discussion to mention that advanced possibilities such as workflow composition or provenance tracking are mostly enabled by this original feature.

1.14 “Another description standard (specifically, an execution standard for tools much like CWL) which closely aligns itself with enabling the FAIR principles is Boutiques (https://boutiques.github.io); consider referencing this standard, and in particular, the tooling it provides to facilitate fair curation of records (more details here: https://figshare.com/articles/poster/fair-pipelines-poster_pdf/8143241).”
We thank the reviewer for pointing us to this relevant work we were not aware of. We have indeed added it , and its contribution to software FAIRness, to the related work we refer to in the paper.

1.15 “The design considerations section provides a list that could be of extreme value to tool and standard developers. Could this be provided as an independent resource or checklist that is made more widely available?”
We thank the reviewer for his interest in this content. Following his suggestion, we added it to the public documentation of biotoolsSchema, it can be found at
https://biotoolsschema.readthedocs.io/en/latest/design_considerations.html.

1.16 “The scope for biotoolsSchema is unclear for the majority of the manuscript, and should be placed closer to the beginning. In particular, the relationship or relative objectives with this and execution standards such as CWL or bioinformatics ontologies such as EDAM.”
We have moved the complete "Scope" subsection to the "Findings" section, where we believe it helps get a better overview of biotoolsSchema. A detailed comparison of the relationship and relative objectives of "registry-focused" (e.g. biotoolsSchema) and "execution-focused" (e.g. CWL) tool descriptions was published in a previous paper (https://link.springer.com/article/10.1007/s10009-015-0392-z), which we now cite in this article.

1.17 “FAIR terms should be added to the table which compares ontologies (in particular, the 15 as enumerated here: https://www.force11.org/group/fairgroup/fairprinciples), possibly in the Force11 column.”
The FAIR principles listed at this URL were mapped and analysed with respect to software in the article “Towards FAIR principles for research software" (https://content.iospress.com/articles/data-science/ds190026). We have in turn added to the article (in new Table 6) a summary of how bio.tools and biotoolsSchema supports each of these principles. The detailed mapping of indicators of FAIRness to biotoolsSchema attributes, with respect to producing a set of objective and transparent metrics of FAIRness will be the subject of a future work using the Tool Information Profile system, as outlined at length in a previous point (see point 1.10).

1.18 “Text in the legends, as well as parts of the figure itself, for figures 1, 2, and 3 is unreadable.”
We have provided larger / higher resolution versions of Figures 1, 2 and 3 which are more readable.

Response to reviewer #2

2.1 "All schemas benefit greatly from being community driven, and the authors do note that extensive

community consultation has been undertaken to arrive at a community consensus as to the content of the schema, but provide few details of the mechanism that was employed that has led to the consensus. I would recommend that inclusion of this information is critical to illustrate that the schema is indeed community agreed, and a summary describing who, what and how the community consensus was reached would be beneficial (e.g. details of workshops, working group membership etc), as any governance type arrangements that have led to each version being agreed / 'signed off'"
We thank Reviewer 2 for his interest in this important aspect of our work. As mentioned in other places in this letter, the overall work of community development of biotoolsSchema is part of a wider process also involving the development of the bio.tools registry, the EDAM ontology and other components of the ELIXIR Tools Platform, as outlined in a recent publication (cite https://academic.oup.com/bib/article/21/5/1697/5560007). The schema development has been in context of major European infrastructure projects (BioMedBridges, ELIXIR EXCELERATE) and ELIXIR national node infrastructures and has leveraged their governance structures, e.g. ELIXIR EXCELERATE WP1 partners. The current governance structure can be seen at https://biotoolsschema.readthedocs.io/en/latest/contributors.html. Over the years biotoolsSchema development has featured at many hackathons, meetings and workshops, using agile methods (e.g. feature poker, sprints etc.) with open participation (within and beyond ELIXIR). It would be too verbose to describe in detail all of this (which are summarised at https://biotools.readthedocs.io/en/latest/events.html), so we have added a short summary on the governance, and how we arrived at a community consensus to the section "Development process and status" of the manuscript, and hope this will suffice.

2.2 “The authors state that "future changes will be pragmatic, driven by community-use cases". The paper would benefit from inclusion of clear guidelines or instructions on how the wider community can provide feedback which may influence the future development of the schema - ie. how to provide feedback on v3.3.0 and how to get involved in influencing any future versions.”
The mechanisms of community engagement around biotoolsSchema (and other technologies in its orbit) have been mentioned in https://academic.oup.com/bib/article/21/5/1697/5560007. We have updated the text (in section "Development process and status") to provide a short summary and refer to the paper mentioned. We have also added contribution guidelines to the online docs (https://biotoolsschema.readthedocs.io/en/latest/what_is_biotoolsschema.html#how-to-contribute-to-biotoolsschema) and link to these from a new CONTRIBUTING.md file (hhttps://github.com/bio-tools/biotoolsSchema/blob/master/CONTRIBUTING.md) in biotoolsSchema repo.

2.3 “Some commentary on the potential sustainability of the schema would be useful - is its use recommended, mandated or required by any groups? The authors briefly discuss the involvement of the ELIXIR consortium in its development, and I note that three controlled vocabularies exist for ELIXIR platform, community and node, so I am guessing that its use is at least recommended by ELIXIR. Some clarification around the use of the schema in ELIXIR and any other other efforts would be valuable to help illustrate how widespread adoption is / is likely to be moving forward.”
Reviewer 1 also raised a comment (see point 1.7) about the sustainability; we have added a short paragraph (in the section "Development process and status") to describe how biotoolsSchema development is (through it’s anchoring within the ELIXIR infrastructure) sustainable including a note about its current and likely future adoption. In short, bio.tools is supported by ELIXIR Europe, and is one of the commissioned services (https://elixir-europe.org/about-us/commissioned-services/registry-tools) of this organization. As such, not only do bio.tools and biotoolsSchema involve the work of multiple national groups (e.g., in Denmark, France and Norway), but they are funded and evaluated with the specific goals of ensuring their long term availability and sustainability.

2.4 “Table 3 states that there are 16 controlled vocabularies, however 18 are listed and 18 are included in the online documentation https://biotoolsschema.readthedocs.io/en/latest/controlled_vocabularies.html.”
This error has been corrected.

2.5 ”Figures 2 and 3 are quite small / low resolution - these would benefit from being larger / higher resolution.”
We have included larger versions of Figures 2 and 3.

2.6 “Similar to Figure 2, Figure 3 should also include a note that the illustration example is for the ProCon tool.”
We have included the note as suggested.

Close
