# Supplementary material for: biotoolsSchema: a formalized schema for bioinformatics software description
Source: Gigascience. 2021 Jan 27;10(1):giaa157. doi: 10.1093/gigascience/giaa157 (PMC7842104; doi:10.1093/gigascience/giaa157)
Supplement: giaa157_Reviewer_1_Report_Original_Submission — Gregory Kiar -- 8/6/2020 Reviewed [file giaa157_reviewer_1_report_original_submission.pdf]

Reviewer Report

Title: biotoolsSchema : a formalised schema for bioinformatics software description

Version: Original Submission **Date:** 8/6/2020

Reviewer name: Gregory Kiar

Reviewer Comments to Author:

This paper presents biotoolsSchema, a controlled metadata standard that emphasizes human- and machine-interpretability, with the hopes of enabling rich and precise descriptions for tools in bioinformatics. This schema is clearly a valuable contribution to bioinformaticians and their research, and has benefited from significant adoption since the inception of it and its predecessors in 2012. There are several points which should be clarified or addressed prior to the publication of this manuscript, at which point I believe it will be an excellent contribution to the field. The main issue at present is the lack of evidence that biotoolsSchema supports the FAIR principles; in particular, there is no reference to immutability or persistence, and the minimum required set of metadata is far too sparse to reach adequate description requirements to be considered FAIR.
-----/
/-----
Comments regarding the schema:
- What is the justification for 50 attributes? It is unclear if or how this list extensible over time. If this standard can evolve, why state 50, and if it cannot, what assurances are there that it will continue to be an effective descriptive schema in the future? There are instances where this list already seems restrictive, such as "accessibility" in Table 3 which appears to support any of three options, though it is easy to imagine many more distinct types of access control, for instance.
- The relationship between CWL or other execution standards and biotoolsSchema is presently unclear. Can a CWL definition be included/referenced within a biotoolsSchema? Similarly, what is the line between an execution standard and what is included in biotoolsSchema? For instance, it appears as though inputs and outputs are defined here, which is a considerable portion of the execution standard. This apparent duplication of content between tool descriptions gives rise to the possibility of inconsistency between them. If an execution record is referenced, are there validators which could be used to ensure consistency of duplicated information across both records?
- Does this schema/manuscript propose a mechanism for storage or access of these records aside from the bio.tools website? What assurance that this website and service will persist beyond a funding cycle, for instance? (i.e. Is it supported by a large public group organization? Could it rely upon such a service, e.g. Zenodo?). If this it not addressed, the records would not live up to the FAIR requirement of persistency and immutability.
- Please discuss the justification for making such a large majority of the fields optional. If the intent is to truly have richly described and queryable tools, the bar for flexibility appears to currently be set too low for this goal to be reached, also limiting the strength of the claim that the metadata is "high quality". The current flexibility may have serious consequences on the consumption of described tools, such as in

the case where licensing information is not provided or known by consumers. While the schema supports the FAIR curation of tools when well implemented, the usefulness of this schema is severely limited if the minimum specification does not.
- It would be valuable to query existing descriptions in bio.tools and see what portion of them meet the standard of being FAIR. This analysis could be included as a sample use-case showcasing the value of biotoolsSchema, as well, and provide further justification and clarification around its adoption.
-----/
/-----
Comments regarding the manuscript:
- Much of the Comparison to related efforts section reads more like a list than flowing text. Please add supporting text to make this read more naturally, and situate biotoolsSchema explicitly relative to these efforts, emphasizing novel elements.
- The mention of tasks at the beginning of the manuscript it not mentioned or referenced later on once the schema has been presented. The efficacy for this schema at accomplishing each task should be explicitly mentioned as its features or attributes are introduced and discussed.
- The explicit comparison of features or interfaces between tools is an excellent feature, and I think it should be more prominently mentioned.
- Another description standard (specifically, an execution standard for tools much like CWL) which closely aligns itself with enabling the FAIR principles is Boutiques (https://boutiques.github.io); consider referencing this standard, and in particular, the tooling it provides to facilitate fair curation of records (more details here: https://figshare.com/articles/poster/fair-pipelines-poster_pdf/8143241).
- The design considerations section provides a list that could be of extreme value to tool and standard developers. Could this be provided as an independent resources or checklist that is made more widely available?
- The scope for biotoolsSchema is unclear for the majority of the manuscript, and should be placed closer to the beginning. In particular, the relationship or relative objectives with this and execution standards such as CWL or bioinformatics ontologies such as EDAM.
- FAIR terms should be added to the table which compares ontologies (in particular, the 15 as enumerated here: https://www.force11.org/group/fairgroup/fairprinciples), possibly in the Force11 column.
- Text in the legends, as well as parts of the figure itself, for figures 1, 2, and 3 is unreadable.
-----/

Level of Interest

Please indicate how interesting you found the manuscript: Choose an item.

Quality of Written English

Please indicate the quality of language in the manuscript: Choose an item.

Declaration of Competing Interests

Please complete a declaration of competing interests, considering the following questions:

- Have you in the past five years received reimbursements, fees, funding, or salary from an organisation that may in any way gain or lose financially from the publication of this manuscript, either now or in the future?
- Do you hold any stocks or shares in an organisation that may in any way gain or lose financially from the publication of this manuscript, either now or in the future?
- Do you hold or are you currently applying for any patents relating to the content of the manuscript?
- Have you received reimbursements, fees, funding, or salary from an organization that holds or has applied for patents relating to the content of the manuscript?
- Do you have any other financial competing interests?
- Do you have any non-financial competing interests in relation to this paper?

If you can answer no to all of the above, write 'I declare that I have no competing interests' below. If your reply is yes to any, please give details below.

I declare that I have no competing interests.

I agree to the open peer review policy of the journal. I understand that my name will be included on my report to the authors and, if the manuscript is accepted for publication, my named report including any attachments I upload will be posted on the website along with the authors' responses. I agree for my report to be made available under an Open Access Creative Commons CC-BY license (http://creativecommons.org/licenses/by/4.0/). I understand that any comments which I do not wish to be included in my named report can be included as confidential comments to the editors, which will not be published.

Choose an item.

To further support our reviewers, we have joined with Publons, where you can gain additional credit to further highlight your hard work (see: https://publons.com/journal/530/gigascience). On publication of this paper, your review will be automatically added to Publons, you can then choose whether or not to claim your Publons credit. I understand this statement.

Yes Choose an item.
