# Supplementary material for: biotoolsSchema: a formalized schema for bioinformatics software description
Source: Gigascience. 2021 Jan 27;10(1):giaa157. doi: 10.1093/gigascience/giaa157 (PMC7842104; doi:10.1093/gigascience/giaa157)
Supplement: giaa157_Reviewer_2_Report_Original_Submission — Jeff Christiansen -- 8/6/2020 Reviewed [file giaa157_reviewer_2_report_original_submission.pdf]

Reviewer Report

Title: biotoolsSchema : a formalised schema for bioinformatics software description

Version: Original Submission **Date:** 8/6/2020

Reviewer name: Jeff Christiansen

Reviewer Comments to Author:

The manuscript submitted by Ison et al entitled "biotoolsSchema : a formalised schema for bioinformatics software description" outlines as the title suggests, a schema that enables formalised and standardised descriptions of bioinformatics software and services.
The biotoolsSchema sits behind the bio.tools Bioinformatics Tools and Services Discovery Portal which has been the subject of a recent paper that outlines the general features of that system (Ison et al, 2019 Genome Biol. 20:164. doi: 10.1186/s13059-019-1772-6), and describes how the use of lodging descriptions of bioinformatics software and services in registries such as bio.tools can aid in researchers being able to find, understand, compare and use bioinformatics tools for a variety of uses and under various circumstances. This task would otherwise be almost impossible by trawling through vast numbers of items describing such tools on platforms such as github and the like - usually with no observance of standardised language to describe features of those software.
The current manuscript complements the previous work by drilling down and providing a thorough description of the biotoolsSchema that sits behind and underpins the bio.tools registry. It is this schema that enables the critically important formalised and standardised descriptions of software and services to be crafted and allow these standardised descriptions to be utilised through both human-readable and machine-readable mechanisms.
The manuscript initially describes the challenges faced without such formalised and standardised descriptors, and discusses how the authors have built the biotoolsSchema through conducting extensive community consultation over several years to arrive at a community consensus.
The authors go on to describe the schema in detail (it defines 50 attributes ranging across technical, scientific and administrative, that are grouped into 9 logical groups (summary, functions, labels, links, downloads, documentation, relationships, publications and credits)). Also included is a description of controlled vocabularies that are applied to enable these attributes to be described using standardised terminology. For scientific concepts, the biotoolsSchema requires the use of the established EDAM controlled vocabulary, and the authors have developed a further 18 controlled vocabularies to enable technical features of the software (ranging across features such as tool type, operating system, license, etc) to be described using standard descriptors.
The authors then compare the biotoolsSchema with other schemas that can be used to describe softwares. As illustrated in Table 5, none of the schemas produced from those efforts have the breadth and richness of attributes or descriptors offered by bio.tools for bioinformatics tools and services.
Overall the paper is a straightforward, thorough and clear description of the motivation and implementation of the schema. Thankyou for your work.
Feedback:

1. All schemas benefit greatly from being community driven, and the authors do note that extensive community consultation has been undertaken to arrive at a community consensus as to the content of the schema, but provide few details of the mechanism that was employed that has led to the consensus. I would recommend that inclusion of this information is critical to illustrate that the schema is indeed community agreed, and a summary describing who, what and how the community consensus was reached would be beneficial (e.g. details of workshops, working group membership etc), as any governance type arrangements that have led to each version being agreed / 'signed off'.
2. The authors state that "future changes will be pragmatic, driven by community-use cases". The paper would benefit from inclusion of clear guidelines or instructions on how the wider community can provide feedback which may influence the future development of the schema - ie. how to provide feedback on v3.3.0 and how to get involved in influencing any future versions.
3. Some commentary on the potential sustainability of the schema would be useful - is its use recommended, mandated or required by any groups? The authors briefly discuss the involvement of the ELIXIR consortium in its development, and I note that three controlled vocabularies exist for ELIXIR platform, community and node, so I am guessing that its use is at least recommended by ELIXIR. Some clarification around the use of the schema in ELIXIR and any other other efforts would be valuable to help illustrate how widespread adoption is / is likely to be moving forward.
4. Table 3 states that there are 16 controlled vocabularies, however 18 are listed and 18 are included in the online documentation https://biotoolsschema.readthedocs.io/en/latest/controlled_vocabularies.html.
5. Figures 2 and 3 are quite small / low resolution - these would benefit from being being larger / higher resolution.
6. Similar to Figure 2, Figure 3 should also include a note that the illustration example is for the ProCon tool.

Level of Interest

Please indicate how interesting you found the manuscript: Choose an item.

Quality of Written English

Please indicate the quality of language in the manuscript: Choose an item.

Declaration of Competing Interests

Please complete a declaration of competing interests, considering the following questions:

- Have you in the past five years received reimbursements, fees, funding, or salary from an organisation that may in any way gain or lose financially from the publication of this manuscript, either now or in the future?

- Do you hold any stocks or shares in an organisation that may in any way gain or lose financially from the publication of this manuscript, either now or in the future?
- Do you hold or are you currently applying for any patents relating to the content of the manuscript?
- Have you received reimbursements, fees, funding, or salary from an organization that holds or has applied for patents relating to the content of the manuscript?
- Do you have any other financial competing interests?
- Do you have any non-financial competing interests in relation to this paper?

If you can answer no to all of the above, write 'I declare that I have no competing interests' below. If your reply is yes to any, please give details below.

I declare that I have no competing interests

I agree to the open peer review policy of the journal. I understand that my name will be included on my report to the authors and, if the manuscript is accepted for publication, my named report including any attachments I upload will be posted on the website along with the authors' responses. I agree for my report to be made available under an Open Access Creative Commons CC-BY license (http://creativecommons.org/licenses/by/4.0/). I understand that any comments which I do not wish to be included in my named report can be included as confidential comments to the editors, which will not be published.

Choose an item.

To further support our reviewers, we have joined with Publons, where you can gain additional credit to further highlight your hard work (see: https://publons.com/journal/530/gigascience). On publication of this paper, your review will be automatically added to Publons, you can then choose whether or not to claim your Publons credit. I understand this statement.

Yes Choose an item.
